# Sparfloxacin reveals a host–virus dual-target antiviral scaffold against respiratory syncytial virus

**DOI:** 10.1038/s42003-026-10744-5

**Published:** 2026-08-08

**Authors:** Yuka Takumi-Tanimukai, Soh Yamamoto, Yurie Yoshida, Tatsuki Takahashi, Akira Takasawa, Akira Katsuyama, Takumi Nishimaki, Haruka Maeda, Katsumi Mizuta, Masashi Idogawa, Yukari Mitsuhashi, Yume Takayanagi, Sithumini M. W. Lokupathirage, Keitaro Yoshida, Keisuke Yamamoto, Takuya Kakuki, Sumito Jitsukawa, Toyotaka Sato, Hirofumi Sawa, Yasuko Orba, Akihiko Sato, Takashi Kojima, Wataru Kamitani, Satoshi Ichikawa, Tetsuya Nosaka, Kenichi Takano, Shin-ichi Yokota, Noriko Ogasawara

**Affiliations:** 1 Division of Microbiology, Department of Infectious Diseases, Sapporo Medical University School of Medicine, Sapporo, Japan; 2 Department of Otolaryngology-Head and Neck Surgery, Sapporo Medical University School of Medicine, Sapporo, Japan; 3 Department of Infectious Diseases and Host Defense, Graduate School of Medicine, Gunma University, Maebashi, Japan; 4 Division of Tumor Pathology, Department of Pathology, Asahikawa Medical University, Asahikawa, Japan; 5 Faculty of Pharmaceutical Sciences, Hokkaido University, Sapporo, Japan; 6 Center for Research and Education on Drug Discovery, Faculty of Pharmaceutical Sciences, Hokkaido University, Sapporo, Japan; 7 Global Station for Biosurfaces and Drug Discovery, Global Institution for Collaborative Research and Education (GI-CoRE), Hokkaido University, Sapporo, Japan; 8 Division of Respiratory Medicine and Allergology, Department of Internal Medicine, Sapporo Medical University School of Medicine, Sapporo, Japan; 9 International Institute for Zoonosis Control, Hokkaido University, Sapporo, Japan; 10 Laboratory for Drug Discovery & Disease Research, Shionogi & Co. Ltd, Osaka, Japan; 11 Yamagata Prefectural Institute of Public Health, Yamagata, Japan; 12 Division of Medical Genome Sciences, Department of Genomic and Preventive Medicine, Sapporo Medical University School of Medicine, Sapporo, Japan; 13 Laboratory of Veterinary Hygiene, Faculty of Veterinary Medicine, Hokkaido University, Sapporo, Japan; 14 Graduate School of Infectious Diseases, Hokkaido University, Sapporo, Japan; 15 One Health Research Center, Hokkaido University, Sapporo, Japan; 16 Veterinary Research Unit, International Institute for Zoonosis Control, Hokkaido University, Sapporo, Japan; 17 Institute for Vaccine Research and Development, Hokkaido University, Sapporo, Japan; 18 Department of Cell Science, Institute of Cancer Research, Sapporo Medical University, Sapporo, Japan; 19 Department of Microbiology and Molecular Genetics, Mie University Graduate School of Medicine, Tsu, Japan; 20 Research Centre for Development of Recombinant VLP Vaccines, Research Institutes of Excellence, Mie University, Tsu, Japan

**Keywords:** Viral host response, Antivirals

## Abstract

Respiratory syncytial virus (RSV) is a major cause of severe respiratory illness across the lifespan, particularly in infants and older adults. Nonetheless, effective antiviral therapies remain limited. Here, we performed a clinically guided, medium-throughput screening of approved drugs used in pediatric populations and identified sparfloxacin (SPFX), a fluoroquinolone antibiotic, as a mechanistically informative antiviral scaffold. SPFX showed antiviral activity against genetically distinct RSV clinical isolates in vitro, and suppressed RSV infection in a mechanistic mouse model in vivo. Mechanistically, our findings support a host–virus dual-target framework in which SPFX suppresses RSV replication through activity consistent with modulation of viral RNA polymerase function together with a pro-viral HSP70-associated host pathway. Using primary human pediatric airway epithelial cells, we identified SPFX-responsive host factors and observed suppression of HSP70 expression independent of infection. Together, these findings establish SPFX as a mechanistically informative antiviral scaffold and support a host–virus dual-target framework for future RSV therapeutic development.

## Abstract (web summary)

Sparfloxacin inhibits respiratory syncytial virus through dual targeting of the viral RNA polymerase and the host HSP70 pathway, providing a promising antiviral scaffold for future RSV therapeutic development.

## Introduction

Respiratory syncytial virus (RSV) is a highly contagious pathogen that imposes a substantial disease burden at both ends of the age spectrum. Nearly all individuals are infected at least once by the age of 2 to 3 years^1,2^, and RSV accounts for approximately 54% of lower respiratory tract infection-related deaths in children under 5 years of age, contributing to over 100,000 pediatric deaths globally each year^2^. Meanwhile, RSV has emerged as a major cause of severe respiratory disease in older adults; in the United States, RSV-associated in-hospital mortality approaches 6% across all age groups^3^. Epidemiological studies have shown that RSV infection in adults aged ≥65 years is associated with pneumonia, exacerbation of chronic cardiopulmonary diseases, hospitalization, and mortality rates comparable to those of seasonal influenza.

Despite this substantial burden, effective treatments and preventive strategies for RSV remain limited. Recent advances in stabilizing the metastable pre-fusion (pre-F) form of the F protein have accelerated vaccine development^4^. Currently, approved options include the anti-F monoclonal antibody palivizumab and the broad-spectrum antiviral ribavirin. However, these drugs show limited efficacy once infection has progressed to the lower respiratory tract^5^, while the clinical use of ribavirin is constrained by toxicity, high cost, and low specificity for RSV^6,7^. Collectively, these limitations underscore the urgent need for new antiviral approaches with improved efficacy and durability.

A comprehensive understanding of the RSV life cycle and its interaction with the host immune system is crucial for identifying new therapeutic targets. RSV is a single-stranded, negative-sense RNA virus encoding 11 proteins, including structural (F, G, SH, and M) and nonstructural (NS1 and NS2) proteins, as well as components of the viral ribonucleoprotein complex (N, P, L, M2-1, and M2-2). Most current antiviral strategies focus on targeting viral proteins, particularly the F protein involved in viral entry^4^ and the nucleocapsid complex^8–11^. While direct-acting antivirals (DAAs) targeting structural proteins face challenges posed by escape mutants, targeting the viral replication machinery, along with essential host cofactors remains a relatively underutilized strategy for achieving durable clinical efficacy.

Targeting host factors, such as molecular chaperones essential for viral protein folding and assembly, represents an attractive complementary or alternative therapeutic approach^12–14^. However, host-directed antiviral strategies raise legitimate concerns regarding safety and off-target effects, particularly in vulnerable populations such as young children. These concerns may be mitigated by leveraging clinically characterized compound classes with established safety profiles, thereby enabling selective modulation of host–virus interactions without broadly suppressing host immunity. Previously, we identified diverse innate immune defenses in upper airway epithelial cells, which are the primary site of RSV entry and replication^15^. Thus, primary pediatric airway epithelial cells provide a physiologically relevant platform for evaluating antiviral scaffolds in a pediatric context.

High-throughput screening of therapeutic candidate compounds using reporter viruses expressing green fluorescent protein (GFP) or luciferase has been widely implemented^16^. However, few identified compounds have progressed to clinical trials, and most of these trials were terminated early due to failure to meet clinical endpoints. These limitations highlight the need for clinically tractable antiviral scaffolds with improved durability and mechanistic versatility.

We hypothesized that clinically used drugs could serve as a source of lead compounds capable of simultaneously modulating viral replication and host cellular pathways, thereby overcoming key limitations of virus-centric antiviral strategies, including resistance and limited durability. Thus, we aimed to streamline anti-RSV drug discovery by integrating primary pediatric airway epithelial models with clinically guided screening of approved drugs, leading to the identification of sparfloxacin (SPFX), a fluoroquinolone antibiotic, as an antiviral scaffold that suppresses RSV replication in vitro and in vivo.

Mechanistically, our findings support a host–virus dual-target framework in which SPFX suppresses RSV replication through activity consistent with modulation of viral RNA-dependent RNA polymerase (RdRp) and a pro-viral HSP70-associated host pathway. Together, these findings identify SPFX as a mechanistically informative antiviral scaffold for RSV therapeutic development.

## Results

### Anti-RSV drug seed screening from pediatric commonly used drugs

**Fig. 1:**
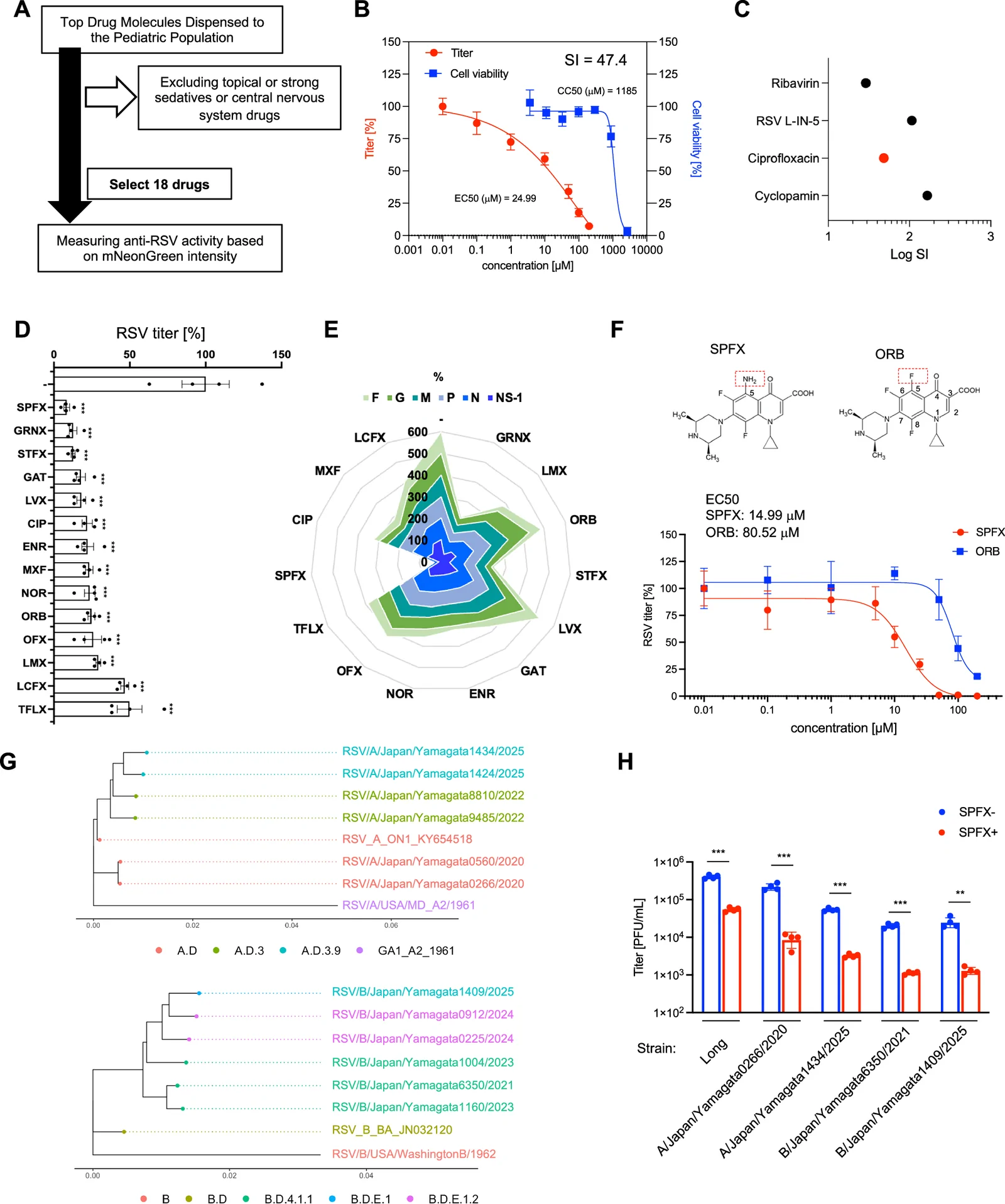
Fluoroquinolones exhibit anti-RSV activity. **A** Schematic overview of the anti-RSV drug screening strategy used in this study. **B** RSV titers in Vero cells at 72 h post-infection (h.p.i.) following treatment with ciprofloxacin (0–200 μM; *n* = 4 biologically independent experiments). Data are shown as mean ± SD. EC₅₀ and CC₅₀ values are indicated. **C** Relationship between EC50 against RSV and CC50 for tested compounds. Selectivity index (SI) was defined as CC50/EC50. Red symbols indicate compounds evaluated in this study. Black symbols indicate reference values for RSV L-IN-5, ribavirin, and cyclopamine from previous reports. **D** RSV titers in A549 cells treated with fluoroquinolones at 24 h. p.i. (MOI = 1). All compounds were tested at a final concentration of 200 μM. Titers in untreated controls were set to 100% (*n* = 4 biologically independent experiments). Data are shown as mean ± SD. ****p < 0.0001 versus untreated controls; ordinary one-way ANOVA with Dunnett’s multiple-comparisons test. Abbreviations are defined in Table 1. **E** Quantification of RSV protein expression levels (radar chart) in the presence of fluoroquinolones, based on data shown in Supplementary Figs. 1D and 2. Data represent mean values from *n* = 4 biologically independent experiments. Protein levels were normalized to β-actin and then to untreated controls (set to 100%). **F** Top, chemical structures of sparfloxacin (SPFX) and orbifloxacin (ORB). The dashed box highlights the structural difference between SPFX and ORB. Bottom, RSV titers in Vero cells at 72 h.p.i. following treatment with the indicated concentrations (0.01, 0.1, 1, 5, 10, 50, 100, and 200 μM; *n* = 4 biologically independent experiments). ata are shown as mean ± SD. EC₅₀ values are indicated. Final solvent concentrations were matched across all conditions. **G** Maximum-likelihood phylogenetic trees of RSV-A (top) and RSV-B (bottom) clinical isolates collected in Yamagata, Japan between 2020 and 2025. Reference strains RSV A2 and RSV B Washington were included for comparison. Clades were assigned using Nextclade annotation. Trees were reconstructed using FastTree and visualized in R using “ape”, “treeio”, and “ggtree”. **H** Effect of SPFX on replication of clinical RSV isolates. Two isolates each of RSV-A and RSV-B were selected based on the phylogenetic analysis shown in (**G**). A549 cells were infected at an MOI of 0.01 and treated with SPFX. Viral titers in culture supernatants were quantified by plaque assay at 48 h.p.i. Data are shown as mean ± SD from *n* = 4 biologically independent experiments. Statistical significance was determined using an unpaired two-tailed Student’s *t*-test versus untreated controls. **p < 0.01, ***p < 0.001.

We began by screening the 30 most frequently prescribed drugs for patients under 17 years of age in the United States^17^ to evaluate their potential anti-RSV activity. We prioritized commonly prescribed pediatric medications to identify clinically tractable compounds with established safety profiles. Central nervous system agonists, strong sedatives, steroids, and topical preparations such as ointments were excluded from further analysis (Fig. 1A and Supplementary Fig. 1A). Next, the remaining 18 drugs were evaluated using a recombinant RSV reporter virus expressing mNeonGreen (RSV-mNeon), which enables direct quantification of viral replication through multi-cycle infection dynamics (Supplementary Fig. 1B–E and Supplementary Table 1).

Among them, ciprofloxacin (CIP), promethazine, lansoprazole, and montelukast showed a reduction in mNeon intensity, and measurable 50% effective concentrations (EC_50_) against RSV were determined to be at 24.99, 28.56, 24.17, and 25.53 μM, respectively. However, except for CIP, strong cytotoxicity was observed at concentrations above 50 μM (Supplementary Fig. 1E). Thus, we focused on CIP and calculated the 50% cytotoxicity concentration (CC_50_), which was 1185 μM (Fig. 1B). The selectivity index (SI) of CIP was 47.4, which was intermediate between that of cyclopamine (CPM), an RSV replication inhibitor that interacts with the M2-1 protein^9,11^, and those of ribavirin and RSV L-IN-5, both RdRp activity inhibitors^18^ (Fig. 1C). Because CIP belongs to a fluoroquinolone class with numerous clinically used structural analogs, we next investigated whether antiviral activity could be enhanced through structure-related optimization.

Subsequently, given the availability of structurally related fluoroquinolones (Supplementary Fig. 2A), we evaluated the anti-RSV activity of fluoroquinolones (Fig. 1D and Supplementary Fig. 2B). Quantification of intracellular RSV protein levels (Fig. 1E and Supplementary Figs. 2B and 3) revealed that SPFX exerted the most potent inhibitory effect, accompanied by a marked reduction in viral titer to 7.8% relative to that of the untreated RSV-infected control (set as 100%). Conversely, orbifloxacin (ORB) exhibited only modest antiviral activity and limited suppression of RSV proteins. Although SPFX and ORB differ structurally by only a single functional group at the 5-position (Fig. 1F), SPFX had significantly superior EC_50_ values and protein-suppressive efficacy (Fig. 1D–F).

To exclude the possibility that the observed reduction in viral protein levels resulted from nonspecific inhibition of cellular protein synthesis or global metabolic suppression, we evaluated the effect of SPFX on ectopic protein expression in transfected cells. SPFX did not reduce GFP expression or RSV structural protein expression in transfected cells, indicating that SPFX does not broadly impair general cellular protein synthesis (Supplementary Fig. 4A–E). To assess antiviral specificity, SPFX was further evaluated against unrelated positive-sense RNA viruses, including Japanese encephalitis (JEV), Zika (ZIKV), and Yellow fever (YFV). At concentrations that effectively inhibited RSV replication, SPFX did not significantly suppress replication of these viruses (Supplementary Fig. 5).

To further evaluate whether the antiviral activity of SPFX extends beyond laboratory RSV strains, we tested its activity against multiple recent clinical RSV isolates. Phylogenetic analysis confirmed that these isolates represented genetically distinct RSV lineages circulating during recent epidemics (Fig. 1G)^19^. Two representative isolates from each RSV A and RSV B lineage were selected for antiviral testing. Treatment with SPFX significantly reduced viral titers across all tested isolates compared with untreated controls (Fig. 1H), indicating that the antiviral activity of SPFX is conserved across genetically diverse RSV strains. These findings are consistent with SPFX acting through a functionally conserved component of RSV replication.

### SPFX suppresses RSV infection in vivo

**Fig. 2:**
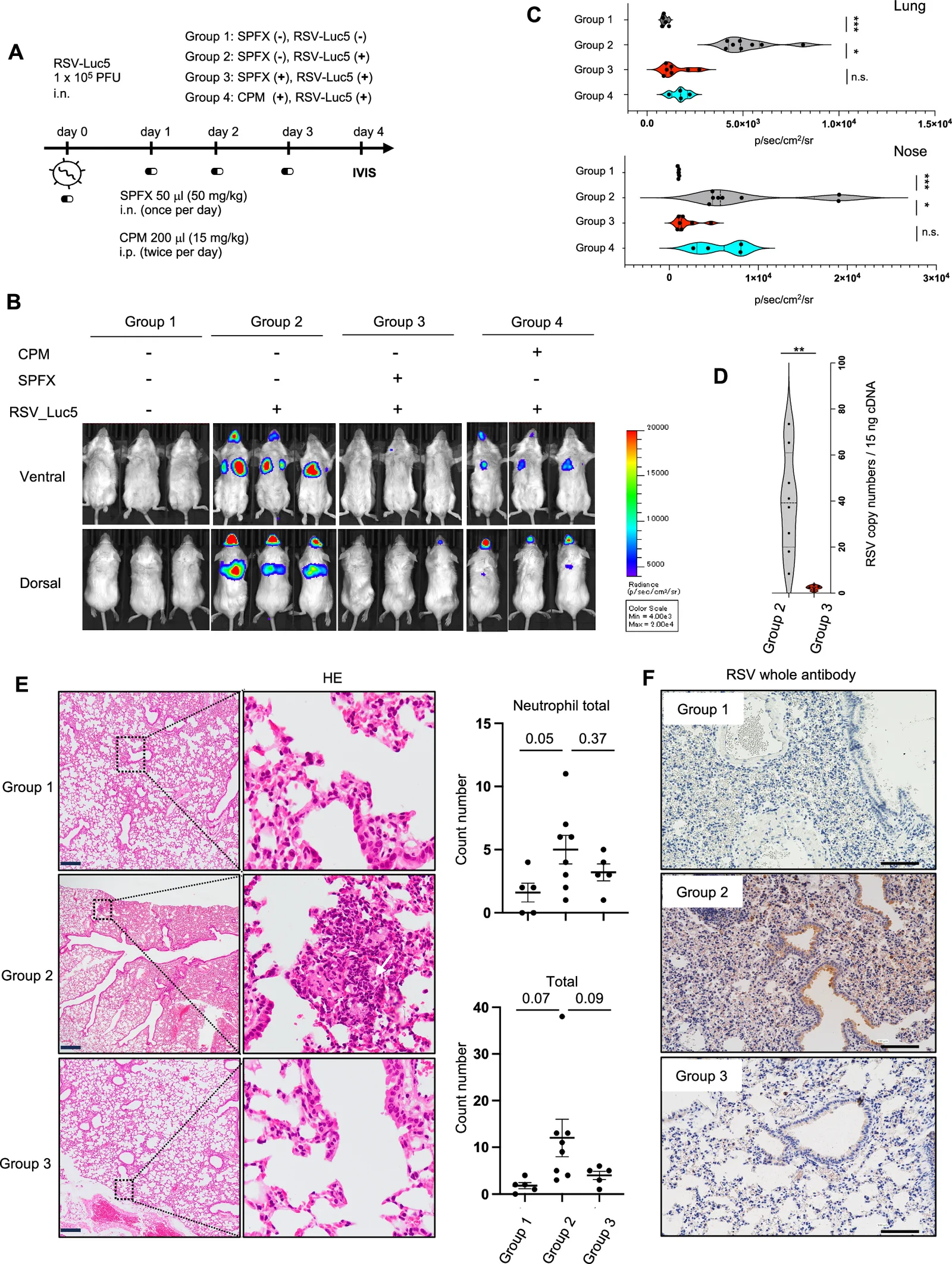
Sparfloxacin (SPFX) effectively suppresses RSV amplification in a mouse in vivo model. **A** Schematic illustration of the experimental design. Recombinant RSV expressing firefly luciferase (RSV-Luc5; 1 × 10⁵ PFU) and SPFX (50 mg/kg) were administered intranasally on day 0. SPFX was subsequently administered once daily. On day 4, bioluminescence signals were monitored using an in vivo imaging system (IVIS). Experimental mice were divided into four groups as indicated in the upper panel: Group 1, uninfected control; Group 2, RSV only; Group 3, RSV + SPFX; Group 4, RSV + cyclopamine (CPM). **B** Representative bioluminescence images from ventral (top) and dorsal (bottom) views. **C** Quantification of luciferase intensity in lung and nasal regions based on ventral and dorsal images, respectively. Data are presented as photons/s/cm²/sr and shown as mean ± SD (*n* = 5–8 animals per group). *p < 0.05, **p < 0.01, ***p < 0.001 versus infected untreated controls (Group 2); Kruskal–Wallis test followed by Dunn’s multiple-comparisons test. Individual-level data are shown in Supplementary Figs. 7 and 8B, C. Each dot represents one individual animal. **D** Quantification of RSV N gene copy number in total RNA extracted from lung tissue. Data are shown as mean ± SD. **p < 0.01; Student’s *t*-test. **E** Hematoxylin and eosin staining of lung tissue. Left and right images show low- and high-magnification views, respectively. White arrows indicate neutrophil infiltration. Right panels show quantification of neutrophils (top) and combined neutrophils plus lymphocytes (bottom). Scale bar, 200 μm. **F** Immunohistochemical staining of lung tissue using goat anti-RSV antibody. Scale bar, 100 μm.

To determine whether the antiviral activity of SPFX observed in vitro translated to measurable antiviral effects in vivo, we evaluated SPFX using an firefly luciferase-expressing RSV (RSV-Luc5) mouse infection model. Female BALB/c mice (8 weeks old) were intranasally inoculated with RSV-Luc5. Longitudinal  in vivo imaging confirmed progressive luciferase signal accumulation in the nasal cavity and lungs over 4 days, consistent with previous reports^20^ (Supplementary Fig. 6). Accordingly, mice were inoculated with RSV-Luc5 on day 0, followed by daily intranasal administration of SPFX (50 mg/kg) (Fig. 2A). As a positive control, CPM was administered twice daily according to Ballester et al.‘s protocols^9^. After 4 days, the RSV replication rate was quantified using an in vivo imaging system (IVIS) (Fig. 2B and Supplementary Fig. 6 and 7). No substantial weight loss was observed in RSV-infected mice, whereas modest body weight reductions (~10%) were observed in the CPM- and SPFX-treated groups (Supplementary Fig. 8A).

In the RSV-infected group (Group 2), luciferase signals were detected in the nasal cavity and lungs (Fig. 2B, C and Supplementary Figs. 6–8). Conversely, luciferase levels were reduced in both the SPFX-treated and RSV-infected group (Group 3) and the CPM-treated and RSV-infected group (Group 4) compared to those of untreated RSV-infected mice (Group 2). SPFX treatment reduced luciferase signals to levels comparable to, or numerically lower than, those observed with CPM treatment.

Quantitative polymerase chain reaction (qPCR) analysis further demonstrated a significant reduction in RSV N gene copy number in the lungs of SPFX-treated mice compared to that in the untreated RSV-infected group (Fig. 2D). Additionally, histopathological evaluation showed that RSV infection induced pronounced inflammatory-cell infiltration, whereas SPFX treatment was associated with reduced inflammatory-cell accumulation and a more limited distribution of inflammatory lesions (Fig. 2E and Supplementary Fig. 8D). Consistently, immunohistochemical staining using anti-RSV antibodies demonstrated widespread RSV antigen distribution in the lungs of infected mice, whereas SPFX treatment markedly reduced viral antigen staining, accompanied by lower viral RNA levels (Fig. 2F). Together, these complementary virological, histopathological, and imaging analyses support antiviral activity of SPFX in vivo in this mechanistic RSV infection model.

Because SPFX showed substantially stronger antiviral activity than the structurally related analog ORB both in vitro and in vivo, we next investigated molecular mechanisms underlying this differential antiviral activity.

### SPFX inhibits the RdRp activity of RSV L protein

**Fig. 3:**
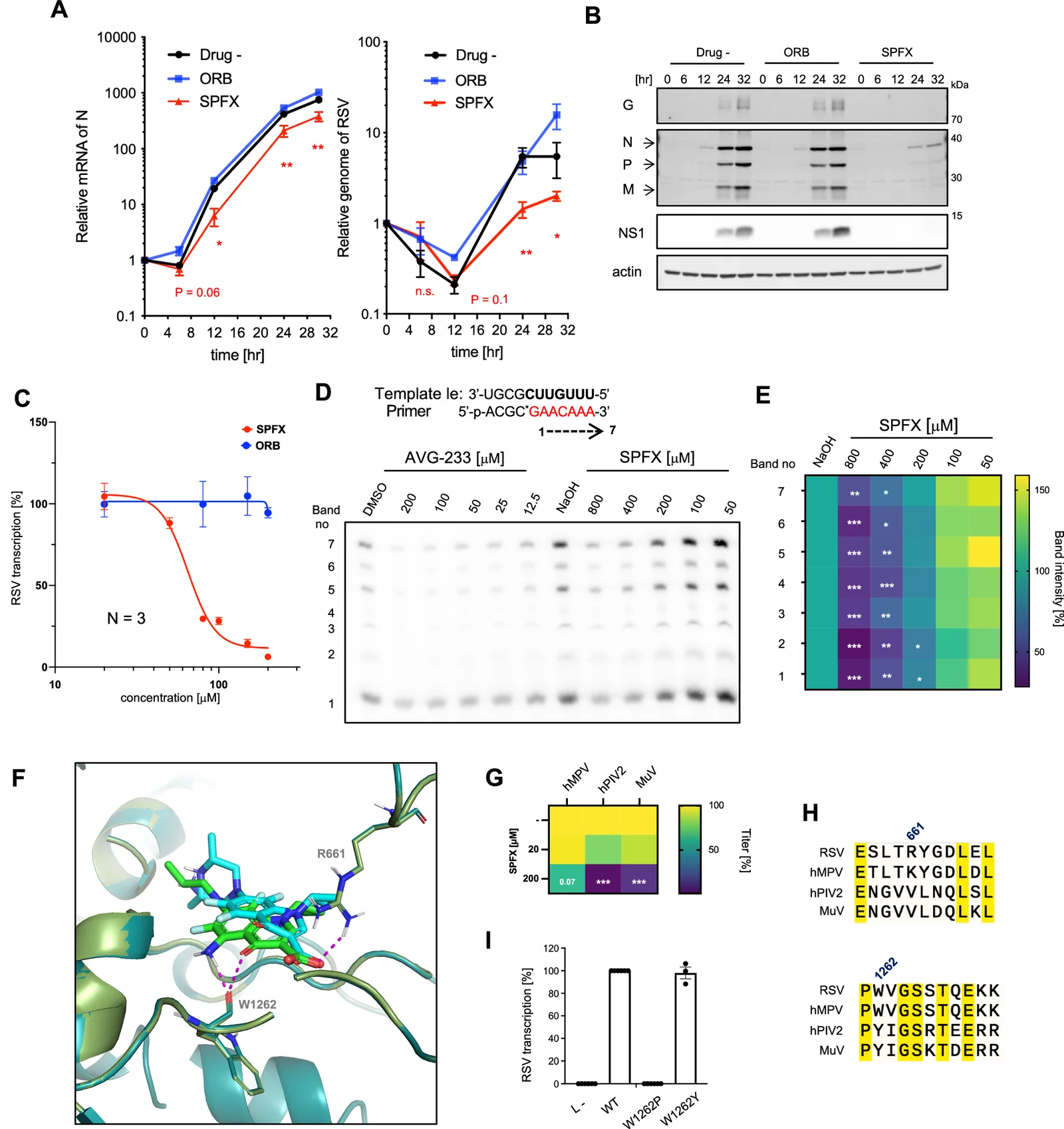
Sparfloxacin (SPFX) inhibits RSV L protein polymerase activity. **A** Time-course analysis of RSV N mRNA (left) and genomic RNA (right) levels in the absence of drug or in the presence of SPFX (200 μM) or ORB (200 μM). Data are shown as mean ± SEM from six independent experiments. *p < 0.05 versus ORB at each time point; Student’s *t*-test. n.s., not significant. **B** Western blot analysis of RSV proteins at the indicated time points following drug treatment. **C** RSV minigenome assay. Luciferase activity in no-drug samples was set to 100%. Data are shown as mean ± SD from three independent experiments. **D** In vitro primer extension assay using purified RSV L and P proteins in the presence of AVG-233 or SPFX. Template and primer sequences are shown above. Red characters indicate nucleotides incorporated by RdRp-mediated elongation. Incorporation of [α-³²P]GTP was visualized by autoradiography following separation on a 22.5% denaturing sequencing gel. Numbers on the left indicate extension products. **E** Heatmap of band intensities from (**D**) (*n* = 3). Values represent mean intensities from three independent experiments. Intensities in the no-drug condition were normalized to 100%. *p < 0.05, **p < 0.01, ***p < 0.001 versus no-drug control; Student’s *t*-test. **F** Structural mapping of predicted SPFX-binding residues on the RSV L protein. Green and cyan indicate SPFX and ORB, respectively. **G** Heatmap of viral titers at 24 h.p.i. following SPFX treatment (*n* = 4). ***p < 0.0001 versus no-drug control; ordinary one-way ANOVA with Dunnett’s multiple-comparisons test. **H** Amino acid sequence alignment of L proteins from human metapneumovirus (hMPV), human parainfluenza virus type 2 (hPIV2), and mumps virus (MuV). Highly conserved residues are highlighted in yellow. **I** RSV minigenome assays using L protein variants at residue W1262. In (**I**), luciferase activity of wild-type L protein (WT) without SPFX was set to 100%. Data are shown as mean ± SD from *n* = 3–6 independent experiments.

To determine whether SPFX interferes with RSV replication machinery, we compared its antiviral effects with those of the structurally related analog ORB in A549 cells. SPFX treatment delayed the expression of RSV N mRNA and the amplification of genomic RNA compared to the untreated control and ORB-treated samples (Fig. 3A). Consistently, SPFX treatment also suppressed the expression of RSV proteins, including the non-structural protein NS1 (Fig. 3B). Additionally, a minigenome assay revealed that SPFX reduced luciferase activity—a surrogate marker of RSV RNA replication and mRNA transcription—more effectively than ORB (Fig. 3C). Northern blot analysis further supported transcriptional suppression (Supplementary Fig. 9). Collectively, these results suggest that SPFX interferes with processes associated with RdRp activity of the RSV L protein. To directly assess this hypothesis, we conducted in vitro polymerase assays using purified RSV L and P proteins (Supplementary Fig. 10A). SPFX and AVG-233^8^ (a known RSV RdRp inhibitor) inhibited RNA elongation from a template in a dose-dependent manner in a primer extension assay (Fig. 3D, E). SPFX also inhibited de novo RNA synthesis, although higher concentrations were required than those of AVG-233 (Supplementary Fig. 10B).

To further probe the interaction between SPFX and the RSV polymerase complex, we serially passaged RSV in the presence of increasing concentrations of SPFX (20–80 μM) over 11 passages, named RSV^SPFX11^ at the final passage (Supplementary Fig. 11A). RSV^SPFX11^ showed partial adaptation at approximately the EC50 concentration (20 μM; Fig. 1F), whereas viral replication remained strongly suppressed at higher SPFX concentrations, indicating incomplete resistance under the tested conditions (Supplementary Fig. 11B, C).

Next-generation sequencing (NGS) of viral genomes from each passage group revealed distinct mutation profiles. After excluding synonymous substitutions and variants detected in the vehicle control group, RSV^SPFX11^ harbored several L gene variants, including L720Q (29.6%), S910G (25.98%), and F1131L (12.39%) (Supplementary Fig. 11D). However, limiting-dilution cloning failed to isolate genetically homogeneous viruses carrying these substitutions, suggesting that these variants remained minority populations and were not directly associated with stable SPFX resistance (Supplementary Fig. 11E). Time-course analyses of several monoclonal isolates further revealed increased replication rates and shorter doubling times during the exponential phase of infection (12–28 h post infection) compared with those of the parental virus (Supplementary Fig. 11F–G). This altered replication kinetics likely explains the increased viral titers in the passaged population at 20 μM (Supplementary Fig. 11B).

We then performed molecular docking analysis using SwissDock to predict potential SPFX-binding sites in the RSV L protein. Candidate binding pockets—designated ai, fu, and gu—were identified (Supplementary Fig. 12A–C). The gu pocket exhibited the most favorable predicted binding energy and stable molecular dynamics behavior and was therefore prioritized for further analysis. Docking analysis suggested interactions between SPFX and residue W1262 within the priming loop region of the RSV L protein (Fig. 3F and Supplementary Fig. 12D, E). In contrast to SPFX, ORB rapidly dissociated from this predicted binding pocket during simulation (Supplementary Fig. 12F).

To functionally evaluate candidate residues identified by docking simulation, we conducted a minigenome assay using L protein mutants. Because docking analysis suggested selective interaction of SPFX—but not ORB—with W1262, we focused on this residue for mutational analysis (Fig. 3F–H). We generated alanine, tyrosine, phenylalanine, methionine, glycine, glutamine, and proline substitution mutants at W1262 and evaluated their effects using a minigenome assay (Fig. 3I and Supplementary Fig. 12G, H). Except for W1262Y and W1262F, all substitutions reduced RdRp activity by >90%. Notably, W1262P showed the strongest suppression of polymerase activity and exhibited reduced SPFX responsiveness (Fig. 3I and Supplementary Fig. 12G, H). However, because most substitutions at W1262 markedly impaired polymerase activity, interpretation of SPFX responsiveness at this residue is inherently limited, and the detailed analyses are therefore presented in Supplementary Fig. 12G, H.

We further attempted to generate recombinant RSV encoding selected candidate substitutions using reverse genetics. Despite multiple independent rescue attempts, no viable recombinant viruses carrying the W1262 substitution could be recovered, suggesting that the W1262 substitution imposes substantial fitness constraints on viral replication.

These findings suggest that SPFX acts through interactions associated with a structurally constrained region of the L polymerase centered on W1262.

### Host-targeted mechanism of SPFX-mediated anti-RSV activity

**Fig. 4:**
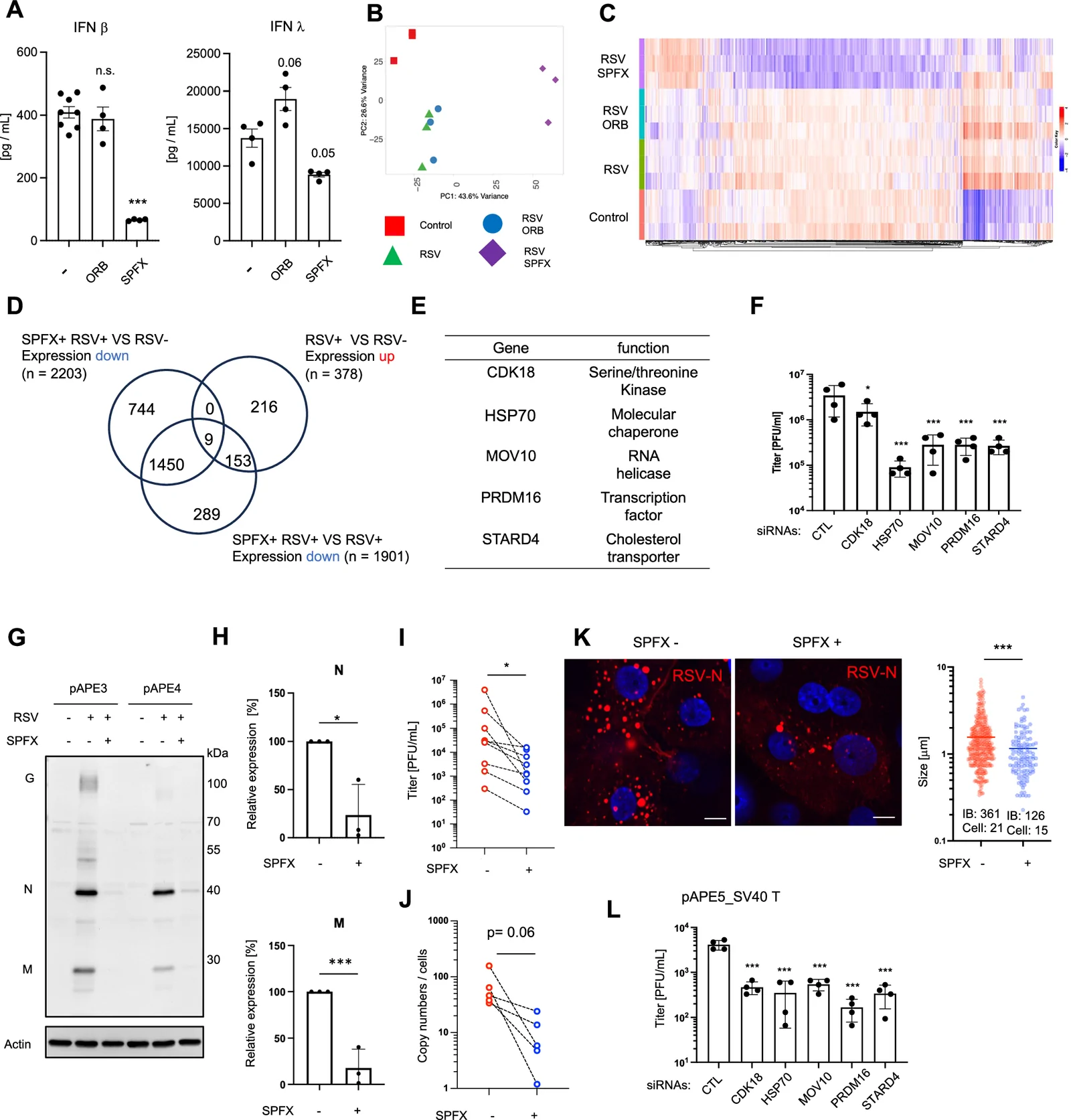
Sparfloxacin-regulated host genes contribute to RSV production in A549 and human pediatric adenoid primary epithelial (pAPE) cells. **A** Protein levels of interferon (IFN)-β (left) and IFN-λ (right) measured by ELISA at 24 h post-infection (h.p.i.) in RSV-infected cells treated with ORB or SPFX (200 μM). Data are shown as mean ± SD from four to eight independent experiments. *p < 0.05, ***p < 0.001 versus drug-free group; ordinary one-way ANOVA with Dunnett’s multiple-comparisons test. n.s., not significant. **B** Principal component analysis of bulk RNA sequencing data. Red squares, green triangles, blue circles, and purple diamonds represent uninfected, RSV-infected, RSV-infected + ORB (200 μM), and RSV-infected + SPFX (200 μM) groups, respectively. **C** Heatmap of differentially expressed genes identified by bulk RNA sequencing. Each condition represents three independent experiments. **D** Venn diagram showing candidate genes identified by bioinformatic analyses from (**B**, **C**) using the limma-voom pipeline. **E** Candidate host genes implicated in RSV replication. **F** RSV titers in A549 cells following siRNA-mediated knockdown of candidate genes at 48 h.p.i. (MOI = 0.1). Data are presented as mean ± SD from four independent experiments. *p < 0.05, ***p < 0.001 vs. negative control (CTL) siRNA-transfected cells; ordinary one-way ANOVA with Dunnett’s multiple comparisons test. **G** Representative western blot images, and **H** corresponding quantification of RSV protein expression in pAPE cells. *p < 0.05, **p < 0.01, ***p < 0.001; Student’s *t*-test. RSV titers (**I**) and (**J**) viral genome copy number in pAPE cells treated with or without 200 μM SPFX at 48 h.p.i. (MOI = 0.1). **K** Immunofluorescence images of RSV N protein (left) and quantification of inclusion body (IB) size (right) in cells treated with or without 200 μM SPFX at 48 h.p.i. Scale bar = 10 μm. The right panel indicates the numbers of IBs and cells counted. ***p < 0.001; Student’s *t*-test. **L** RSV titers in immortalized pAPE cells following siRNA knockdown of genes of interest at 48 h.p.i. (MOI = 0.1). Data are shown as mean ± SD from four independent experiments. ***p < 0.001 vs. CTL siRNA-transfected cells; ordinary one-way ANOVA with Dunnett’s multiple comparisons test.

Because SPFX suppressed RSV replication more efficiently than ORB despite their close structural similarity, we next investigated whether SPFX alters host cellular pathways associated with RSV replication. Fluoroquinolones have been reported to exhibit immunomodulatory properties^21,22^, raising the possibility that SPFX may additionally modulate host responses relevant to RSV infection. Notably, type I (α/β) and III (λ) interferons (IFNs), which are induced by RSV infection, were differentially suppressed by fluoroquinolones, with varying degrees of inhibition among compounds (Fig. 4A and Supplementary Fig. 13A). SPFX significantly suppressed the production of both IFNs, whereas ORB had no inhibitory effect (Fig. 4A). Because SPFX and ORB exhibited distinct effects on IFN responses despite structural similarity, we next performed RNA sequencing to identify SPFX-responsive host pathways associated with antiviral activity. Principal component analysis revealed that RSV infection induced a specific transcriptional profile. ORB treatment caused minimal transcriptional changes, whereas SPFX treatment induced a gene expression pattern clearly distinct from those of the untreated control, RSV-infected, and ORB-treated groups (Fig. 4B). Furthermore, SPFX treatment induced a unique set of differentially expressed genes (Fig. 4C). To identify host factors associated with the RSV life cycle that are modulated by SPFX, we first extracted genes upregulated by RSV infection (*n* = 378) and those downregulated by SPFX during RSV infection (*n* = 1901), identifying 162 overlapping genes. To prioritize SPFX-responsive host factors, we further focused on genes selectively downregulated by SPFX in both infected and uninfected conditions, yielding nine candidate genes identified using Limma-Voom (Fig. 4D). Independent analysis using DESeq2 identified the same candidate gene set, supporting the robustness of this prioritization strategy (Supplementary Fig. 13B). Among the six genes commonly identified by both Limma-Voom and DESeq2 analyses, the expression of *TCAF2* was also downregulated by ORB (Supplementary Fig. 13C), suggesting that this gene may be a general fluoroquinolone target rather than a SPFX-specific effect. Accordingly, we prioritized the remaining five genes—*CDK18, HSPA1A, MOV10, PRDM16,* and *STARD4*—for downstream functional analyses (Fig. 4E and Supplementary Fig. 13D, E). Knockdown of these genes using small interfering RNA (siRNA) in A549 cells significantly reduced infectious RSV titers (Fig. 4F and Supplementary Fig. 13F), supporting their functional contribution to efficient RSV replication. To evaluate these findings in a physiologically relevant pediatric airway model, we employed pediatric adenoid-derived primary epithelial (pAPE) cells^23,24^, which recapitulate a major upper airway site of RSV infection^15,23^. Expression of RSV structural proteins G, N, and M was detected 48 h post-infection, confirming that RSV efficiently infects and replicates in pAPE cells. SPFX treatment significantly reduced the expression levels of these RSV proteins across pAPE cells derived from multiple donors (Fig. 4G, H). Consistent with this reduction, SPFX-treated pAPE cells also exhibited decreased infectious viral titers and lower copy numbers of the RSV N gene (Fig. 4I, J). Furthermore, SPFX treatment reduced the number and size of inclusion bodies (IBs), as visualized by RSV N staining. Since IBs function as membraneless organelles supporting virus replication and transcription^25^, these results further confirm the inhibitory effect of SPFX on RSV replication in pAPE cells (Fig. 4K).

To facilitate siRNA-based functional validation in a pediatric airway epithelial context, we generated immortalized pAPE cells using simian virus 40 large T antigen (SV40 T) (pAPE_SV40 T cells). Silencing of the five SPFX-responsive host genes in pAPE_SV40 T cells led to a significant decrease in RSV titers (Fig. 4L). These results confirm that all five host factors are involved in the RSV life cycle.

### HSP70 involvement in the host-targeted mechanism of SPFX

**Fig. 5:**
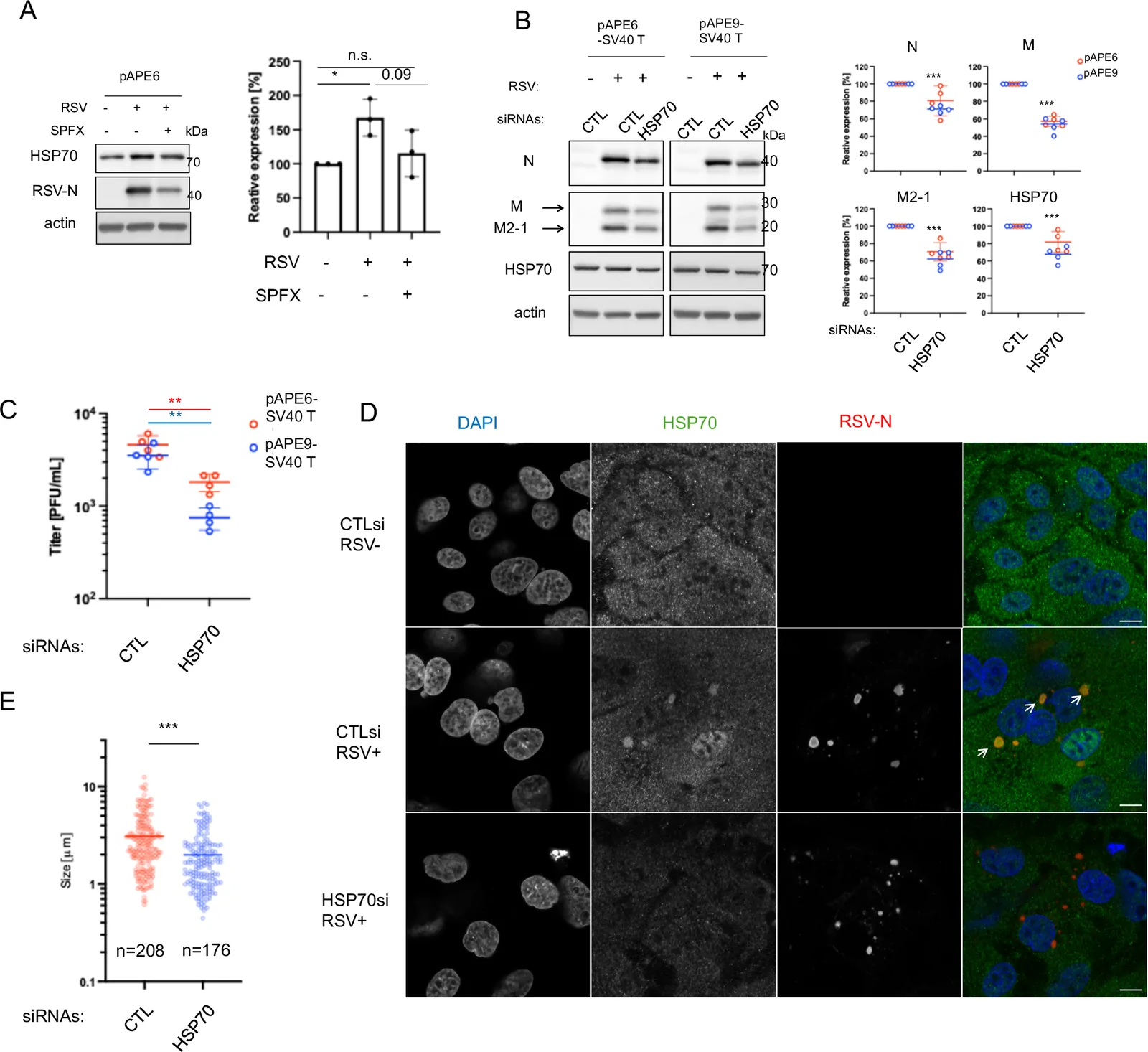
Heat shock protein 70 (HSP70) promotes production of infectious RSV by regulating inclusion body (IB) formation in pAPE cells. **A** Representative western blots (left) and quantification of HSP70 expression (right) in pAPE cells treated with SPFX (200 μM) and harvested at 48 h post-infection (h.p.i.; MOI = 0.1). *p < 0.05; ordinary one-way ANOVA with Dunnett’s multiple-comparisons test (*n* = 3). **B** Representative western blots (left) and quantification of HSP70 and RSV protein expression (right) in pAPE-SV40T cells following HSP70 knockdown at 48 h.p.i. (MOI = 0.1). ***p < 0.001 versus negative-control (CTL) siRNA-transfected cells; unpaired two-tailed Student’s *t*-test. **C** RSV titers in immortalized pAPE cells following HSP70 knockdown at 48 h.p.i. (MOI = 0.1). **p < 0.01 versus CTL siRNA-transfected cells; unpaired two-tailed Student’s *t*-test. **D** Immunofluorescence images of HSP70 (green) and RSV N protein (red) in pAPE-SV40T cells (no. 9) at 48 h.p.i. (MOI = 5). White arrows indicate inclusion bodies (IBs). Scale bar, 10 μm. **E** Quantification of IB size in HSP70-knockdown cells shown in (**D**). Numbers within the bars indicate the number of IBs analyzed. *p < 0.05; unpaired two-tailed Student’s *t*-test.

Among the five identified host factors, *HSPA1A* was prioritized because its knockdown produced the strongest and most reproducible reduction in RSV replication across both A549 and pAPE-derived systems. *HSPA1A* encodes the stress-inducible chaperone HSP70^26^. We found that HSP70 was upregulated in pAPE cells upon RSV infection and downregulated by SPFX treatment; these changes correlated with the RNA profiles shown in (Figs. 4C–E and 5A). To determine whether SPFX directly modulates host pathways independently of viral infection, we next examined its effects in non-infected cells. SPFX treatment resulted in a reduction of HSP70 at both protein and mRNA levels (Supplementary Fig. 14A–C). These findings indicate that SPFX suppresses HSP70 expression independently of RSV infection.

Therefore, we next investigated the functional contribution of HSP70 in pAPE_SV40 T cells. Transfection with siRNA targeting HSP70 reduced protein levels even under RSV infection conditions (Fig. 5B). Consistently, HSP70 knockdown suppressed the expression of viral structural proteins, including N, M, and M2-1, across pAPE_SV40 T cells derived from multiple donors (Fig. 5B). This was accompanied by a reduction in infectious RSV titers (Fig. 5C), indicating that HSP70 is required for efficient RSV replication.

Furthermore, immunofluorescence analysis revealed co-localization of HSP70 with IBs as marked by RSV N protein during RSV infection in pAPE-SV40 T cells (Fig. 5D). HSP70 knockdown led to a notable reduction in the size of IBs, further supporting its involvement in RSV replication (Fig. 5E). Because inclusion bodies (IBs) support RSV genome replication and transcription, we next examined whether HSP70 associates with components of the viral replication machinery. HSP70 and L were detected in the immunoprecipitated fraction, indicating that HSP70 associated with components of the RSV replication machinery. (Supplementary Fig. 14D).

Together, these findings support HSP70 as a host factor contributing to efficient RSV replication and suggest that modulation of HSP70-associated processes contributes to the antiviral activity of SPFX.

## Discussion

Here, we identified fluoroquinolones as mechanistically informative antiviral lead scaffolds against RSV through a targeted screening approach using clinically characterized compounds, thereby addressing some limitations of conventional high-throughput screening strategies. Using this framework, we found that SPFX exerts anti-RSV activity through effects consistent with modulation of both viral replication machinery and host pathways associated with efficient RSV replication. Together, our findings support a host–virus dual-target framework in which SPFX suppresses RSV replication through activity associated with both viral polymerase function and host pathways supporting viral replication. These results position fluoroquinolones as mechanistically informative antiviral scaffolds rather than directly repurposable therapeutic agents.

SPFX is a fluoroquinolone derivative that shares the characteristic bicyclic core structure^27^. Comparative analysis between SPFX and its structural analog, ORB, revealed that substitution at the 5-position is essential for suppressing RSV replication. Fluoroquinolones are generally avoided in pediatric populations due to cartilage toxicity concerns^28–31^. Accordingly, SPFX should be viewed primarily as a proof-of-concept antiviral scaffold rather than a clinically deployable RSV therapeutic. Future development toward clinical application would require medicinal chemistry optimization to improve safety while minimizing antibacterial activity, accompanied by dedicated juvenile toxicology and pharmacology studies. The structure–activity relationship analysis in this study suggests that specific structural features of fluoroquinolones may underlie their anti-RSV activity, supporting the rational design of derivatives with reduced side effects and minimal antibacterial activity.

In addition, the in vivo antiviral activity of SPFX was evaluated using a BALB/c mouse model infected with recombinant luciferase-expressing RSV. This imaging-based system enables longitudinal monitoring of infection dynamics and is well suited for mechanistic evaluation of antiviral activity. Bioluminescence imaging showed concordance with independent measures of lung viral burden, supporting the biological relevance of this model. However, because BALB/c mice do not fully recapitulate the permissive infection dynamics observed in more advanced RSV models such as cotton rats, future translational studies will require validation in additional animal systems. Consistent with this interpretation, SPFX retained antiviral activity against genetically distinct RSV clinical isolates in vitro, supporting antiviral activity beyond the recombinant reporter strain.

With respect to viral targets, SPFX suppressed RdRp activity in our minigenome assay, a highly conserved enzyme essential for viral genome replication and transcription, which has been widely studied^10,32^. Trp 1262, located in the priming loop, functions as a critical priming residue responsible for initial RNA synthesis by RdRp; loss of its aromatic ring disrupts genome replication and mRNA transcription^33,34^. These findings are consistent with a model in which SPFX binding may alter priming-loop flexibility, leading to reduced polymerase activity through perturbation of a structurally constrained region of the RSV polymerase complex. The strong functional constraint surrounding W1262 may also limit the evolutionary space available for stable resistance acquisition, consistent with the incomplete resistance observed during serial passage experiments. Further research is needed to identify SPFX-binding residues and analyze priming loop movement to guide the rational design of fluoroquinolone anti-RSV drugs.

HSP70 participates in multiple stages of viral replication across diverse viral families^35–37^. In RSV infection, HSP70 expression was upregulated, whereas HSP70 knockdown reduced viral titers and structural viral protein levels, supporting its role as a host-supportive factor for RSV replication. RSV replication occurs within cytosolic inclusion bodies (IBs), which serve as sites of viral genome replication and transcription^25^. Our findings that HSP70 co-localized with IBs, associated with RSV polymerase components, and altered IB architecture following knockdown support a model in which HSP70 contributes to efficient RSV replication by stabilizing viral replication compartments. Consistent with this interpretation, HSP70 has also been implicated in maintaining inclusion body integrity in other paramyxoviruses^38^. Together, these findings suggest that SPFX may partially suppress RSV replication through modulation of HSP70-dependent processes involved in maintenance of viral replication compartments.

We additionally identified four host factors—*CDK18, MOV10, PRDM16*, and *STARD4*—that may contribute to efficient RSV replication. These genes likely support distinct aspects of the viral life cycle, including cytoskeletal organization, cholesterol trafficking, and interferon regulation. For example, CDK18 has been implicated in focal adhesion kinase/Rho signaling pathways^39^, which may influence intracellular trafficking of RSV ribonucleoprotein complexes^20^. Similarly, STARD4-mediated cholesterol transport may support viral particle production, as cholesterol metabolism is known to influence replication of several enveloped viruses, including RSV^40–44^. Although MOV10 is generally regarded as an antiviral RNA helicase^45,46^, our findings suggest that it may instead function in a pro-viral manner during RSV infection. PRDM16 has been reported to suppress IFN-stimulated gene expression in adipocytes^47^, raising the possibility that its depletion may indirectly restrict RSV replication through enhanced antiviral signaling. While the precise contribution of these host factors requires further mechanistic investigation, our findings suggest that RSV replication depends on a broader host regulatory network than previously appreciated.

Compared with currently available RSV therapeutics, including ribavirin and monoclonal antibodies targeting the RSV F protein, the antiviral framework described here supports a complementary strategy based on simultaneous modulation of viral polymerase activity and host pathways required for efficient viral replication. Rather than functioning as a directly repurposable antiviral agent, SPFX may therefore represent a mechanistically informative starting point for development of more durable RSV therapeutics with reduced susceptibility to resistance.

Notably, antiviral activity was consistently observed across transformed cell lines, primary pediatric airway epithelial cells, and an in vivo infection model, supporting the robustness of the observed effects. The incorporation of pediatric adenoid-derived epithelial cells further strengthens the physiological relevance of this study, as RSV primarily infects the upper airway during early childhood. Because SPFX also suppressed HSP70 expression in the absence of infection, our findings further support the concept that clinically characterized compounds may reveal host pathways exploitable for antiviral intervention.

In conclusion, our findings establish SPFX as a quinolone-derived antiviral scaffold that suppresses RSV replication through activity associated with both viral RNA polymerase function and host pathways supporting efficient viral replication. Together, these findings support a host–virus dual-target framework for RSV therapeutic development and provide a mechanistic foundation for future optimization of quinolone-derived antiviral compounds.

### Limitations of the study

Several limitations of this study should be acknowledged. First, infectious viral titers in lung homogenates were not quantified by plaque assay in the in vivo experiments. Therefore, antiviral activity in mice was evaluated using multiple complementary measurements, including IVIS imaging of luciferase reporter activity, lung viral RNA quantification, viral antigen distribution, and histopathological assessment, each reflecting distinct aspects of RSV infection biology. Although concordant reductions were observed across these endpoints, direct quantification of infectious viral titers will be an important addition in future studies.

Second, a non-infected SPFX-treated control group was not included in the original in vivo experimental design. Consequently, the transient body weight reduction observed in SPFX-treated RSV-infected mice cannot be definitively attributed to compound-related toxicity, infection–treatment interactions, or both. Future studies incorporating drug-only control groups will be required to better define the in vivo tolerability profile of SPFX and related derivatives.

Third, although resistance selection experiments, docking analyses, and reverse-genetics studies implicated residues such as W1262 in SPFX responsiveness, we were unable to generate viral mutants that simultaneously exhibited robust polymerase activity and complete resistance to SPFX. Because W1262 resides within the highly constrained priming loop region of the RSV polymerase, future structural and biophysical studies will be necessary to define SPFX-binding interactions and conformational effects more precisely.

Finally, primary adenoid epithelial cells used in this study were derived from donors treated at a single center in East Asia, and all donors were of Asian ethnic background. Nevertheless, the consistent antiviral effects observed across transformed cell lines, primary epithelial cultures, and the mouse infection model suggest that the principal findings are unlikely to be strongly influenced by donor background. Validation in more diverse donor populations will nonetheless be important in future studies.

## Methods

### Cell culture and virus preparation

Human laryngeal carcinoma HEp-2 cells (American Type Culture Collection [ATCC], Manassas, VA, USA) were cultured in minimum essential medium (MEM) (FUJIFILM Wako, Tokyo, Japan) containing 10% (v/v) fetal bovine serum (FBS) with 100 U/mL penicillin G, and 10 mg/mL streptomycin (P/S) (FUJIFILM Wako). A549 cells (ATCC) were cultured in RPMI 1640 medium (Nacarai Tesque, Kyoto, Japan) with 10% (v/v) FBS and P/S. Baby hamster kidney cells expressing T7 RNA polymerase (BHK/T7-9) were obtained from RIKEN BRC (Wako, Japan) and maintained in Dulbecco’s modified Eagle medium (DMEM) supplemented with 0.6 mg/mL hygromycin, 10% (v/v) FBS, and P/S. Lenti-X 293T and 293T cells were obtained from Takara Bio Inc. (Kusatsu, Japan) and ATCC, respectively. Both cells were cultured in DMEM supplemented with 10% (v/v) FBS and P/S. All cell lines tested negative for mycoplasma contamination, and cells at passage numbers ≤20 were used in all experiments after thawing.

RSV Long (VR-26) and A2 strains (VR-1540) were obtained from ATCC. GFP-expressing RSV (RSV-GFP1) and firefly luciferase-expressing RSV (RSV-Luc5) were purchased from ViraTree (Durham, NC, USA). These RSV strains were grown in HEp-2 cells. Information on GFP-expressing human metapneumovirus and mumps virus was described in previous reports^48,49^. Human parainfluenza virus type 2 (hPIV2) was provided by Dr. Ito (Mie University, Tsu, Japan)^50^, and was amplified using Vero cells (KAC Co., Ltd., Kyoto, Japan), which were maintained in MEM supplemented with 10% FBS (v/v) and P/S. BHK-21 cells were purchased from the American Type Culture Collection (ATCC; Manassas, VA, USA). Briefly, BHK-21 cells and dengue virus II (DENVII; D2/hu/INDIA/09-74), Japanese encephalitis virus (JEV; Beijing-1), Zika virus (ZIKV; MR766), and Yellow fever virus (YFV; 17D-204) were incubated in 96-well plates with twofold serially diluted SPFX, OBFX, and ribavirin (*n* = 2).

A recombinant respiratory syncytial virus expressing mNeonGreen (RSV-mNeon) was generated using a reverse genetics system. The mNeonGreen reporter gene was inserted between the P and M genes of the RSV A2 antigenomic cDNA, flanked by authentic RSV gene start and gene end transcription signals. The full-length antigenomic plasmid was co-transfected into BHK/T7-9 cells together with support plasmids encoding the RSV N, P, L, and M2-1 proteins to facilitate virus rescue under T7 RNA polymerase–driven transcription. The recovered virus was subsequently amplified in HEp-2 cells. Viral stocks were clarified by low-speed centrifugation, aliquoted, and stored at −80 °C until use. Expression of mNeonGreen was confirmed by fluorescence microscopy, and viral titers were determined using a plaque assay on HEp-2 cells.

### Phylogenetic analysis of clinical RSV isolates

Clinical RSV isolates collected in Yamagata, Japan between 2020 and 2025 were provided by the Yamagata Prefectural Institute of Public Health. Viral RNA was extracted from cultured isolates using the QIAamp Viral RNA Mini Kit (QIAGEN) according to the manufacturer’s instructions. Whole-genome sequencing was performed as described in the Next-generation sequencing section.

Genome sequences were aligned using MAFFT (v7.526) with the “--auto” option. Phylogenetic trees were constructed from the aligned sequences based on pairwise genetic distances calculated using the ‘dist.ml‘ function in the “phangorn” package (R version 4.4.2), followed by tree inference using the neighbor-joining algorithm implemented in the ‘NJ‘ function of the “ape” package.

Lineage assignments were determined using a nearest-reference approach. Representative sequences for each lineage (based on Nextclade classification) were included as references, and each clinical isolate was assigned to the lineage of its closest reference sequence based on tree-based patristic distances. The resulting phylogenies were visualized using the ‘ggtree‘ package in R.

### Reagents and resources

All reagents, compounds, and resources are listed in Table 1. Chemical structures of all quinolones (Supplementary Fig. 2A) were drawn using ACD/ChemSketch (Freeware, 2021.1.3, ACD/Labs, Ontario, Canada).

**Table 1:** Compounds and reagents

Reagent, resource, or compound	Abbreviation	Source	Identifier
Compound			
Amoxicillin	-	Tokyo Chemical Industry	A2099
Azithromycin	-	Tokyo Chemical Industry	A2076
Albuterol (Salbutamol)	-	Selleck	S2507
Clavulanate	-	FUJIFILM Wako	097923
Cephalexin	-	Selleck	S1502
Nystatin	-	LKT Labs	N9874
Trimethoprim	-	FUJIFILM Wako	209-18381
Sulfamethoxazole	-	FUJIFILM Wako	199-10451
Acetaminophen	-	FUJIFILM Wako	015-22651
Chlorpheniramine	-	FUJIFILM Wako	030-23281
Ciprofloxacin	CIP	Selleck	S2027
Promethazine	-	Selleck	S5196
Antipyrine	-	FUJIFILM Wako	018-11112
Ranitidine	-	Selleck	S5662
Lansoprazol	-	Selleck	S1354
Montelukast	-	Selleck	S5783
Ibuprofen	-	Selleck	S1638
Cefdinir	-	Selleck	S1605
Cyclopamin	CPM	Selleck	S1146
Sparfloxacin	SPFX	Sigma-Aldrich	56968
Orbifloxacin	ORB	Selleck	S4150
Garenoxacin	GRNX	Selleck	S5908
Lascufloxacin	LCFX	MedChemExpress	HY-16745
Ofloxacin	OFX	Selleck	S1463
Tosufloxacin	TFLX	Selleck	E4847
Moxifloxacin	MXF	FUJIFILM Wako	M487460
Levofloxacin	LVX	FUJIFILM Wako	BIL1009
Sitafloxacine	STFX	Selleck	S2152
Lomefloxacin	LMX	Selleck	S5491
Gatifloxacin	GAT	Selleck	S1340
Norfloxacin	NOR	Selleck	S1509
Enrofloxacin	ENR	LKT Laboratories, Inc.	E5369
Alpha-^32^P-GTP (3000 Ci/mmol)	-	Revvity	BLU006H
**Antibody**			
Goat anti-whole RSV antibody	-	Abcam	Ab20745
Mouse anti-RSV F antibody	-	Abcam	Ab94968
Rabbit anti-RSV NS1 antibody	-	Produced by rabbit using purified recombinant full length NS1	In this study.
Mouse anti-RSV N	-	Abcam	Ab94806
Rabbit anti-HSP70	-	^61^	
Mouse anti-beta actin antibody	-	FUJIFILM Wako	010-27841
**Plasmids**			
pCMV-VSV-G	-	Addgene	#8454
psPAX2	-	Addgene	#12260
pLV-CMV-SV40-T-puro	-	VectorBuilder	VB900088-2959jeq

### MTT assay

MTT reduction assays were conducted as previously described^51^ to evaluate cell viability following virus-induced cytopathic effects and compound-induced cytotoxicity in HEp-2 cells. Assay conditions for all tested viruses followed protocols described in prior studies^52,53^. Fluoroquinolones were dissolved in 1 M NaOH to generate stock solutions and subsequently diluted in culture medium to the desired working concentrations. To avoid potential confounding effects of solvent dilution, all dose–response assays were performed under fixed solvent conditions, with a final NaOH concentration of 1 mM across all wells, such that only the compound concentration differed. Matched vehicle-only dilution series were performed in parallel to control for solvent effects, and assay conditions were maintained at physiological pH.

### EC_50_ and CC_50_

Overall, 1.2 × 10^5^ Vero cells were seeded into 12-well plates in 1 mL of growth medium and incubated for 24 h. Cells were infected with RSV-mNeon at a multiplicity of infection (MOI) of 0.005. At 2 h post-infection, cells were washed three times with growth medium and then incubated for 72 h in medium supplemented with ciprofloxacin, sparfloxacin, or orbifloxacin. After incubation, culture supernatants were collected, and viral titers were determined.

In addition to the MTT assay, cell viability was independently assessed using an ATP-based luminescence assay (CellTiter-Glo, Promega, Madison, WI, USA) according to the manufacturer’s instructions. This assay quantifies intracellular ATP levels as a surrogate measure of metabolically active viable cells and was performed under experimental conditions matched to those used in the antiviral assays. Luminescence signals were measured using an Infinite 200 Pro M Plex microplate reader (Tecan, Switzerland) and normalized to untreated control cells.

EC_50_ and CC_50_ values were determined using GraphPad Prism (version 10.1.1; GraphPad Software, Boston, MA, USA) by fitting dose–response curves with a four-parameter nonlinear regression model (variable slope). Data were derived from at least three biologically independent experiments. EC_50_ values represent the concentration required to reduce viral replication by 50%, whereas CC_50_ values represent the concentration required to reduce cell viability by 50% relative to untreated controls. Selectivity Index (SI) was calculated as the ratio of CC_50_ to EC_50_:

$${SI}=\frac{{CC}50}{{EC}50}$$

For antiviral assays, non-infected cells were defined as 100% viability, and infected cells treated with vehicle control (0.01% dimethyl sulfoxide) were defined as 0% inhibition. For cytotoxicity assays, untreated cells were used as the 100% viability reference, and compound-treated cells were normalized to vehicle-treated controls. For experiments incorporating the CellTiter-Glo assay, cell viability was quantified based on ATP-dependent luminescence intensity and analyzed using the same dose–response modeling approach described above.

### Titer

The measurement of RSV titer was performed according to a method modified from a previous report^48^. In total, 6 × 10^4^ HEp-2 cells were seeded to a 24-well plate in 0.5 mL growth medium and incubated for 24 h. Next, the medium was removed, and 150 μL of a 10-fold serial diluent of RSV was inoculated and incubated at 37 °C for 2 h with shaking every 15 min. After the inoculum was aspirated, 2 mL of growth medium supplemented with 0.6% (w/v) hydroxypropyl methylcellulose, 20 mM HEPES-NaOH pH 7.4, 2.5 μg/mL amphotericin B, and 50 μg/mL gentamicin (FUJIFILM Wako) was overlaid and incubated for 4 days at 37 °C. On day 4, cells were fixed by adding 0.5 mL of 10% (v/v) formaldehyde-containing 0.15 M saline overnight. After washing with 1× phosphate-buffered saline (-) [PBS (−)], plaques were visualized according to the previous report^48,49^.

### Minigenome assay

Codon-optimized RSV M2-1 cDNA was synthesized by Thermo Fisher Scientific (Waltham, MA, USA). cDNA of N was amplified from the cDNA library of RSV (Long strain) infected cells. These genes were cloned into the pCAGGS plasmid. Human codon-optimized L and P (A2 strain) were synthesized by VectorBuilder and Thermo Fisher Scientific, respectively. Flag-tagged L (Flag-L) and His-tagged P (His-P) cDNAs were subcloned into PCAGGS-woodchuck hepatitis virus post-transcriptional regulatory element (WPRE) plasmid^54^, which was replaced from beta-globin poly tail sequence to WPRE sequence. Mutants of the L gene were generated using NEBuilder HiFi DNA assembly (NEB, Ipswich, MA, USA). The sequence information of RSV minigenome was kindly provided by Dr. Racheal Fearns (Boston University, Boston, MA, USA), and its sequence and additional restriction enzyme restriction sites between the leader and trailer sequence was synthesized by Integra DNA Technologies (IDT, Coralville, IA, USA). This minigenome cDNA was cloned at the LacZa position of the PUC19 plasmid using NEBuilder HiFi DNA assembly. Firefly luciferase cDNA was subsequently inserted into the EcoRI and BamHI sites of the PUC19-RSV minigenome plasmid (Supplementary Fig. 15). Overall, 2.0 × 10^5^ of BHK/T7-9 cells were seeded onto a collagen-coated 12-well plate before the day of transfection. Before transfection, DMEM was changed to an SPFX-containing medium. Then, pCAGGS plasmids encoding N, P, M2-1, L, reporter plasmid, and pRL-TK (Promega, Madison, WI, USA) (molar ratio: 1, 1, 0.5, 0.33, 1, and 0.1) were transfected using Lipofectamine 3000 (Thermo Fisher Scientific). After 24 h incubation, cells were incubated for a further 24 h, followed by medium change. On day 4, cells were rinsed twice with 1× PBS (−), then lysed using 200 μL of cell culture lysis buffer (Promega). Firefly and Renilla luciferase activity was measured using a Dual-Luciferase reporter system (Promega) and a TECAN M1000 Pro system (Tecan, Männedorf, Switzerland). Firefly luciferase activity was normalized using Renilla luciferase activity.

### Mice

All mice used in the study were 8-week-old female BALB/c mice (Sankyo Labo Service Co., Tokyo, Japan) housed under a 12-h/12-h light/dark cycle. This study was approved by the Animal Care Committee of the Sapporo Medical University (permission number: H2204:22-034_23-092). The animal experiments were conducted in accordance with the ARRIVE 2.0 guidelines and included reporting of details such as average body weight, diet, housing conditions, group assignments, and the number of animals per group and per cage. Mice were anaesthetized with a mixture of medetomidine, midazolam, and butorphanol (4.0, 0.3, and 5.0 mg/kg, respectively) by intraperitoneal injection. Mice were infected intranasally with 50 μL of 1xPBS (-, without Ca²⁺/Mg²⁺) containing 1 × 10⁵ PFU of RSV-Luc5. Compounds were dissolved in 1 M NaOH and subsequently neutralized to physiological pH using 3% (v/v) acetic acid, followed by dilution in 1xPBS (-). After intranasal inoculation, mice were immediately recovered by intraperitoneal administration of atipamezole. Mice were treated once daily by intranasal administration in a volume of 50 μL. SPFX was administered intranasally at a dose of 50 mg/kg once daily. As a positive antiviral control, CPM was administered intraperitoneally at a dose of 15 mg/kg twice daily. For in vivo imaging, mice were anesthetized as described above. Mice were intranasally administered 50 μL of 1xPBS (-) containing D-luciferin (75 mg/kg). After 3 min, luciferase activity was imaged using an IVIS Lumina III imaging system (Xenogen, Perkin Elmer, Shelton, CT, USA) with a nose cone providing 100% oxygen. Bioluminescence images were acquired for 3 min with f/stop = 1 and large binning. Nasal photons were quantified within the nasal region of the mouse, and lung photons were quantified within the thoracic region. Photon emission was calculated using Living Image software (v.4.8.2, Perkin Elmer) and expressed as radiance (photons/s). For longitudinal IVIS analyses (Supplementary Fig. 6), repeated measurements obtained from the same animal across multiple time points were not treated as independent biological replicates.

### Quantitative polymerase chain reaction (qPCR)

Total RNA was purified using the RNeasy Plus kit (QIAGEN, Venlo, Netherlands) according to the manufacturer’s protocol. Then, the cDNA was synthesized using the ReverTra Ace qPCR RT master mix with a gDNA remover according to the manufacturer’s protocol (TOYOBO, Osaka, Japan). For kinetics of RSV amplification, poly-A tail primer or anti-genome primer (5′-gcgtacaacaaacttgcataaacc-3′) was used under the reverse transcription step. qPCR was performed using LightCycler480 (Roche, Basel, Switzerland). Primer-probe against the RSV N gene was used for detection and quantification^55^.

### Viral replication kinetics analysis

Viral replication kinetics were quantified assuming exponential growth during early exponential growth phases within defined time intervals. Replication rates (*r*) were calculated using the formula:

$$r=({ln}\,{N}_{2}-{ln}\,{N}_{1})/({t}_{2}-{t}_{1}),$$

where N₁ and N₂ represent viral RNA levels (qPCR) or infectious titers (PFU/mL) at time points t₁ and t₂, respectively. For qPCR data, N values correspond to fold changes calculated as 2^−ΔΔCt^ from RSV N gene expression normalized to ACTNB.

Doubling time (T_d) was calculated as:

$$T{\_}\,d={ln}\,(2)\,/\,r.$$

For qPCR-based analyses, replication rates were calculated between 12 and 28 h post-infection using ΔΔCt-derived fold changes of RSV N gene expression normalized to ACTNB. Each replicate was treated independently for the calculation of replication parameters. Only time intervals showing monotonic increases in viral RNA levels were used for analysis. To ensure the validity of the exponential growth assumption, only time intervals showing monotonic increases in viral RNA levels were used for analysis.

### Hematoxylin-Eosin staining and immunohistochemistry

Mouse lung tissues were fixed in formalin, and sections were prepared from paraffin blocks and stained with hematoxylin-eosin (HE) staining (MorphoTechnology, Sapporo, Japan). The pathological score was defined as below and scored by a pathologist. Clusters of neutrophils or lymphocytes were defined as 10 cells or more. Regarding neutrophil infiltration, level 2 was defined when neutrophils were seen sporadically, and level 1 was defined when one cell or more were seen in the visual field. Unstained sections were dehydrated, infiltrated, and embedded with paraffin, and tissue was sectioned. For antigen retrieval, sections were placed in boiling 10 mM citrate buffer for 20 min and cooled slowly. After rinsing, sections were blocked for endogenous peroxidase activity with 3% hydrogen peroxide/methanol. After rinsing, tissue sections were blocked for nonspecific binding with 2.5% rabbit serum/0.3% Triton-X 100/1 × PBS (−). After avidin/biotin blocking (VectorLabs SP2001), tissue sections were incubated with 40 μg/mL goat anti-whole RSV polyclonal antibody (AB20745, Abcam, Cambridge, UK) for 16 h at 4 °C. Sections were then rinsed and incubated in biotinylated secondary rabbit anti-goat IgG antibody (Vector Laboratories, Burlingame, CA, USA) at a 1:100 dilution for 1 h at room temperature (20–22 °C). After another rinse, sections were incubated in ABC reagent (avidin–biotin–horseradish peroxidase complex; Vector Laboratories) for 1 h at room temperature (20–22 °C). Sections were rinsed again and incubated with ImmPACT™ NovaRED™ reagent (Vector Laboratories) for 5 min at room temperature. Then, they were rinsed in deionized H_2_O, counterstained with hematoxylin, dehydrated, cleaned, mounted, and coverslipped using VECTASHIELD (Vector Laboratories) in preparation for microscopic analysis.

### L and P protein purification using HEK293T

PCAGGS-WPRE Flag-L and His-P plasmids were transfected to HEK293T cells using Polyethyleneimine MAX. Particularly, the molecular ratio of L and P gene-encoding plasmids was 2.029 pmol (15 μg) and 1.222 pmol (5 μg), respectively; a total of 20 μg plasmids were transfected. After 24 h post-transfection, fresh DMEM was changed and incubated for a further 24 h. Flag-L and His-P co-expressing cells were lysed with 0.5% NP40, 0.15 M NaCl, and 20 mM Imidazole in 20 mM Tris-HCl (7.4) following two times washing with cold 1 × PBS (−) and purified according to Ludeke et al.’s protocol^56^. Purified P and L concentrations were measured using the BCA protein Assay kit (Thermo Fisher Scientific).

### Primer extension assay

The primer extension assay was performed according to a method modified from a previous report^57^ using purified L and P proteins. For analysis of the competitive inhibition ability of SPFX and AVG-233, 1 μg purified RSV L and P proteins, 0.2 μM template (3′-UGCGCUUGUUU-5′), and 0.2 mM primer (5′-p-ACGC-3′), were incubated 30 °C for 10 min in reaction buffer [20 mM Tris-HCl (pH 7.5), 1 mM dithiothreitol, 8 mM MgCl_2_, 15 mM NaCl, 10% glycerol, and 0.02 U/μL SUPERase-In RNase Inhibitor (Thermo Fisher Scientific)], and then ATP and CTP (10 μM each), 100 nM of [α-^32^P]-GTP, and AVG-233 or SPFX were added and incubated at 30 °C for 30 min. After incubation, an equal volume of 0.01% bromophenol blue (FUJIFILM Wako) and 0.01% xylene cyanol FF (FUJIFILM Wako) dissolved in formamide was added and incubated at 95 °C for 5 min. Then, 5 μL of sample was separated using 22.5% polyacrylamide urea TBE sequencing gels in 0.5× TBE buffer for 1 h at 3500 V. The gel was dried, and the elongated primer was visualized using FLA3000 G (Cytiva, Tokyo, Japan).

### Next-generation sequencing

Whole-genome amplification of RSV was performed using a modified protocol based on the RSV genome sequencing method^58^. Five amplicons covering the viral genome were pooled in equimolar concentrations for each sample. Library preparation was conducted using the Nextera XT kit (Illumina, San Diego, CA, USA), followed by index addition through PCR amplification and purification using Agencourt AMPure XP beads. The resulting libraries were quantified using the Qubit dsDNA HS Assay Kit (Thermo Fisher Scientific).

A next-generation sequencing (NGS) library was also constructed using the QIAseq FX DNA Library Kit (QIAGEN) and sequenced on the Illumina iSeq platform. Short-read sequences were aligned to the RSV A2 reference genome (GenBank: AY911262) using the BWA-MEM algorithm (v0.7.13-r1126)^59^. Variant allele frequency (VAF) analysis was performed using VarScan version 2.4.3^60^. Mutations detected in the control samples subcultured in the presence of NaOH were excluded and are shown in Supplementary Fig. 11D.

### Docking simulation

The three-dimensional structure of the RSV polymerase protein was retrieved from the Protein Data Bank (PDB code: 6PZK). Initial docking simulations of sparfloxacin were performed using the Swiss-Dock server, targeting the PRNTase domain of the polymerase (residues 968–1460), which generated 256 docking poses. These poses were grouped into 33 clusters by the server, from which three representative poses (ai, fu, and gu) were manually selected based on their docking scores and favorable interactions with the protein.

Each selected pose was then mapped onto the full-length protein chain. The resulting complexes, including the SPFX molecule, were parameterized for molecular dynamics (MD) simulations using the all-atom CHARMM36 force field. The system was solvated with TIP3P water and neutralized with 0.15 M NaCl. Sequential equilibration was conducted at 303 K: first in the NVT ensemble using Nose-Hoover thermostats for 125 ps, followed by the NPT ensemble using Nose-Hoover thermostats and the Parrinello-Rahman barostat for 250 ps, while applying position restraints on the protein heavy atoms. Subsequently, a 50 ns production run was performed. All MD simulations were performed using the GROMACS 2022 software. The Root Mean Square Deviation (RMSD) values of the ligand across the three trajectories were analyzed (Supplementary Fig. 12A). All RMSD fluctuations remained within 0.25 nm, indicating that the docked structures were stable throughout the simulation. To validate the reliability of these docking poses, SPFX was manually mutated to orbifloxacin to serve as a negative control. These poses were refined using Glide and further optimized via Prime MM-GBSA (Supplementary Fig. 12B). The binding affinity of each complex was evaluated by calculating the binding free energy (ΔGbind). The ΔGbind values and key interactions are summarized in Supplementary Fig. 12C. Among the three candidates, the gu pose exhibited the lowest ΔGbind for sparfloxacin, identifying it as the most plausible binding mode. When the same MD simulation was performed for the orbifloxacin-mutated gu pose, the RMSD exceeded 1 nm (Supplementary Fig. 12F), consistent with the Prime MM-GBSA predictions and supporting the specificity of the SPFX interaction.

### RNA sequencing

Total RNA was extracted using the RNeasy Plus kit (QIAGEN). RNA sequencing (RNA-seq) was performed by Rhelixa (Tokyo, Japan) using the NovaSeq 6000 (Illumina). Sequencing was performed with a paired-end 150 bp configuration (PE150), generating 40 million reads per sample (20 million read pairs), corresponding to approximately 6 G bases per sample. Acquired sequence reads were aligned to the human genome sequence (hg38) using STAR. Read counts of each transcript and gene were calculated by RNA-seq by Expectation-Maximization (RSEM). To identify differentially expressed genes and their enrichment, expected counts calculated by RSEM were analyzed using integrated differential expression and pathway analysis (iDEP.96) and ShinyGO0.81 (University of South Dakota, Vermillion, SD, USA). Count data were transformed by EdgeR:log2 (counts per million + 4) for clustering and principal component analysis. Differentially expressed genes were selected by DEseq2 and Limma-Voom algorithm under the condition that the false discovery rate cutoff and minimum fold change were 0.1 and 2, respectively. Volcano plots were generated with the EnhancedVolcano package (version 1.26.0) in R (version 4.5.0). The data supporting the findings of this study are available within the paper and its Supplementary Information files. RNA-seq data have been deposited in the NCBI BioProject database under accession number PRJNA1282552 and will be made publicly available upon publication. Supplementary Data 1 are provided with this paper.

### Small interfering RNA (siRNA)

siRNA (Silencer Select) was purchased from Thermo Fisher Scientific. siRNA was transfected using RNAimax (Thermo Fisher Scientific) according to the manufacturer’s protocols. After 24 h of transfection, the growth medium was changed and incubated for another 24 h. At 48 h post-transfection, RSV was infected for 2 h, and then the cells were rinsed three times with medium. After 48 h post-infection, the titer was determined and calculated as PFU/mL.

### Immunofluorescence

Cells were passaged on a collagen-coated glass bottom dish (Matsunami Glass Ind., Ltd., Osaka, Japan), then incubated for 48 h. Cells were inoculated with RSV at a multiplicity of infection (MOI) = 5–10. After RSV infection, the cells were cultured in BEGM™ BulletKit™ medium containing 10% (v/v) FBS. Cells were fixed after 24 h of infection in 4% (v/v) paraformaldehyde at room temperature for 15 min. Furthermore, specimens were treated with 1× PBS (-) containing 2.5% (v/v) normal goat serum (Abcam) and 0.3% (v/v) Triton-X 100 for 1 h following washing with 1× PBS (−). A mouse anti-RSV N was diluted using 1× PBS (−) containing 0.3% Triton-X 100, and samples were incubated with the diluted antibodies for 16 h at 4 °C. After rinsing with 1× PBS (−), specimens were reacted with Alexa Fluor 594-conjugated goat anti-mouse IgG (Thermo Fisher Scientific) at 25 °C for 1 h. Finally, specimens were mounted with ProLong Gold Antifade reagent with DAPI (#8961, Cell Signaling Technology, Danvers, MA, USA). Fluorescent images were obtained using an ELYRAS.1 LSM780 microscope (Carl Zeiss Microscopy, Jena, Germany).

### pAPE cells

Human adenoids (nasal tonsils) were obtained from patients with obstructive sleep apnea syndrome caused by adenoid hypertrophy. The donors were patients aged 3 to 15 years who underwent adenoidectomy at the Sapporo Medical University Hospital. Informed consent was obtained from all parents of the patients, and this study was approved by the institutional review board of Sapporo Medical University Hospital (302-245). Donors exhibiting clinical symptoms or syndromic conditions beyond simple hypertrophy were excluded from the study. Tissue samples were washed with Dulbecco’s phosphate-buffered saline (D-PBS), mechanically fragmented, and incubated for 16 h at 4 °C in RPMI1640 medium containing 30 µg/mL DNase I and 1 mg/mL type I collagenase. Subsequently, tissues were minced using a gentleMACS dissociator (Miltenyi Biotec, Auburn, CA, USA) and the cells were filtered through 100 µm MACS SmartStrainers (Miltenyi Biotec). Cells were then treated with red blood cell lysis solution (Miltenyi Biotec) and washed with D-PBS. After centrifugation at 800 × *g* for 5 min, the isolated human adenoid epithelial cells were plated with Bronchia; Epithelian Cell Growth Medium Bulletkit (BEGM) (Lonza, Switzerland) containing 10% (v/v) FBS on 100-mm culture dishes, which were coated with rat tail collagen (1 mg/mL in 0.02 N acetic acid, Corning, Corning, NY, USA) in a humidified, 5% CO_2_/95% air incubator at 37 °C^23,24^. On the following day, the medium was replaced with FBS-free BEGM, and cells were maintained until used for experiments. The information on established pAPE cells is listed in Table 2. To generate immortalized pAPE cells, a lentiviral encoding simian virus 40 large T antigen (SV40 T) was generated using Lenti-X 293T cells. The information on plasmids for lentivirus generation is listed in Table 1. After transduction, SV40 T-expressing pAPE cells were maintained BEGM on a collagen-coated cell culture dish, and SV40 T expression was confirmed by western blotting and immunofluorescence.

**Table 2:** Characteristics of adenoid tissue per patient

Patients	NO.	Sex	Age at the time of surgery (months)
Ade25	pAPE1	M	37
Ade26	pAPE2	F	46
Ade28	pAPE3	M	40
Ade31	pAPE4	M	75
Ade36	pAPE5	M	88
Ade41	pAPE6	M	103
Ade54	pAPE7	M	180
Ade61	pAPE8	M	47
Ade62	pAPE9	F	112

### Statistics

All data are presented as mean ± SEM or SD, as indicated in the figure legends. Statistical analyses were performed using GraphPad Prism 10 and R version 4.4.2. Normality was assessed using the Shapiro–Wilk test for comparisons between two groups and the Brown–Forsythe test for comparisons among multiple groups.

For comparisons between two groups, either Student’s *t*-test or Mann–Whitney *U* test was used as appropriate. Paired data were analyzed using paired *t*-tests or Wilcoxon matched-pairs signed-rank tests. For comparisons among multiple groups, one-way ANOVA followed by Holm–Šídák multiple-comparison testing or Kruskal–Wallis test followed by Dunn’s multiple-comparison test was used as appropriate.

Longitudinal IVIS data presented in Supplementary Fig. 6 were analyzed using repeated-measures ANOVA. Correlations were assessed using Spearman’s rank correlation coefficient. A two-sided *P* value < 0.05 was considered statistically significant.

### Reporting summary

Further information on research design is available in the Nature Portfolio Reporting Summary linked to this article.

## Supplementary information

Transparent Peer Review file

Supplementary Information

Description of Additional Supplementary Files

Supplementary Data 1

Reporting Summary

## Data Availability

The data supporting the findings of this study are available within the paper and its Supplementary Information files. Raw RNA-seq data have been deposited in the NCBI BioProject database under accession number PRJNA1282552. Uncropped source images are provided in Supplementary Figs. 16–34. Supplementary Data 1 are provided with this paper. Additional data are available from the corresponding author upon reasonable request.
