# Supplementary material for: Sparfloxacin reveals a host–virus dual-target antiviral scaffold against respiratory syncytial virus
Source: Commun Biol. 2026 Aug 8;9:1434. doi: 10.1038/s42003-026-10744-5 (PMC13722688; doi:10.1038/s42003-026-10744-5)
Supplement: Supplementary file 1 [file 42003_2026_10744_MOESM1_ESM.pdf]

nature portfolio

Peer Review File

Sparfloxacin reveals a host–virus dual-target antiviral scaffold against respiratory syncytial virus

Corresponding Author: Dr Noriko Ogasawara

This file contains all reviewer reports in order by version, followed by all author rebuttals in order by version.

Version 0:

Reviewer comments:

Reviewer #1

(Remarks to the Author)
Dear Editor, Dear Authors

The Authors addressed most of my multiple comments in an adequate way. They clarified that testing the impact of SPFX on the activity of the RdRp with an L protein harboring mutations at W1262 is likely not meaningful as this mutant displays only marginal activity. So please remove panel J from Figure 3. I consider the manuscript eligible for publication.

Reviewer #2

(Remarks to the Author)
The authors conducted a clinically oriented, medium-throughput screening of clinically approved drugs indicated for paediatric use, and identified sparfloxacin (SPFX), a fluoroquinolone-class antibiotic, as a structurally valuable antiviral scaffold for mechanistic investigation. In vitro assays verified that SPFX exerts antiviral efficacy against genetically diverse clinical isolates of respiratory syncytial virus (RSV), and its in vivo anti-RSV potency was further validated using a mouse infection model. Mechanistic studies reveal that SPFX inhibits RSV replication via a dual host–virus targeting mode of action: it interferes with viral RNA polymerase activity while concurrently suppressing the pro-viral host pathway mediated by heat shock protein 70 (HSP70). Collectively, these results highlight the translational potential of SPFX as a promising candidate for future therapeutic development against RSV.
I previously evaluated the initial version of this manuscript submitted to Nature Communications and provided a comprehensive set of peer review comments. I have now examined the revised manuscript and confirm that the authors have thoroughly addressed all my raised concerns, which has substantially strengthened the overall quality of this work. The revised manuscript fully satisfies the rigorous publication criteria of Communications of Biology. Accordingly, I recommend the immediate acceptance of this manuscript.

Open Access This Peer Review File is licensed under a Creative Commons Attribution 4.0 International License, which permits use, sharing, adaptation, distribution and reproduction in any medium or format, as long as you give appropriate credit to the original author(s) and the source, provide a link to the Creative Commons license, and indicate if changes were made.
In cases where reviewers are anonymous, credit should be given to 'Anonymous Referee' and the source.
The images or other third party material in this Peer Review File are included in the article's Creative Commons license, unless indicated otherwise in a credit line to the material. If material is not included in the article's Creative Commons license and your intended use is not permitted by statutory regulation or exceeds the permitted use, you will need to obtain permission directly from the copyright holder.
To view a copy of this license, visit https://creativecommons.org/licenses/by/4.0/

Response to Reviewers

Manuscript ID: COMMSBIO-26-5310-T

Title: "Sparfloxacin reveals a host–virus dual-target antiviral scaffold against respiratory syncytial virus"

Reviewers' comments:

Reviewer #1 (Remarks to the Author):

Dear Editor, Dear Authors

The Authors addressed most of my multiple comments in an adequate way. They clarified that testing the impact of SPFX on the activity of the RdRp with an L protein harboring mutations at W1262 is likely not meaningful as this mutant displays only marginal activity. So please remove panel J from Figure 3. I consider the manuscript eligible for publication.

Response:

We sincerely thank the reviewer for the positive evaluation of our revised manuscript and for this helpful suggestion. As recommended, panel J has been removed from the main Figure 3 and is now presented as Supplementary Fig. 12G. The original Supplementary Figs. 12G and 12H have been renumbered as Supplementary Figs. 12H and 12I, respectively. In addition, we have added a statement to the Results section noting that interpretation of SPFX responsiveness at W1262 is limited because most substitutions markedly impaired polymerase activity.

Results: Page 14, "Because most substitutions at W1262 markedly impaired polymerase activity, interpretation of SPFX responsiveness at this residue is inherently limited. Therefore, the detailed analyses are provided in Supplementary Fig. 12G–I."

Reviewer #2 (Remarks to the Author):

The authors conducted a clinically oriented, medium-throughput screening of clinically approved drugs indicated for paediatric use, and identified sparfloxacin (SPFX), a fluoroquinolone-class antibiotic, as a structurally valuable antiviral scaffold for mechanistic investigation. In vitro assays verified that SPFX exerts antiviral efficacy against genetically diverse clinical isolates of respiratory syncytial virus (RSV), and its in vivo anti-RSV potency was further validated using a mouse infection model. Mechanistic studies reveal that SPFX inhibits RSV replication via a dual host–virus targeting mode of action: it interferes with viral RNA polymerase activity while concurrently suppressing the pro-viral host pathway mediated by heat shock protein 70 (HSP70). Collectively, these results highlight the translational potential of SPFX as a promising candidate for future therapeutic development against RSV.

I previously evaluated the initial version of this manuscript submitted to Nature Communications and provided a comprehensive set of peer review comments. I have now examined the revised manuscript and confirm that the authors have thoroughly addressed all my raised concerns, which has substantially strengthened the overall quality of this work. The revised manuscript fully satisfies the rigorous publication criteria of Communications of Biology. Accordingly, I recommend the immediate acceptance of this manuscript.

Response:

We sincerely thank the reviewer for the careful re-evaluation of our manuscript and for the highly positive assessment of our work. We greatly appreciate the reviewer's constructive comments throughout the review process, which have substantially strengthened the manuscript. We are grateful for the reviewer's recommendation for acceptance.
