# Supplementary material for: Sparfloxacin reveals a host–virus dual-target antiviral scaffold against respiratory syncytial virus
Source: Commun Biol. 2026 Aug 8;9:1434. doi: 10.1038/s42003-026-10744-5 (PMC13722688; doi:10.1038/s42003-026-10744-5)
Supplement: Supplementary file 2 [file 42003_2026_10744_MOESM2_ESM.pdf]

Supplementary Information
1. Supplementary Figures
2. Supplementary Tables
3. Supplementary Methods

Sparfloxacin reveals a host–virus dual-target antiviral scaffold against respiratory syncytial virus

Yuka Takumi-Tanimukai, Soh Yamamoto, Yurie Yoshida, Tatsuki Takahashi, Akira Takasawa, Akira Katsuyama, Takumi Nishimaki, Haruka Maeda, Katsumi Mizuta, Masashi Idogawa, Yukari Mitsuhashi, Yume Takayanagi, Sithumini M. W. Lokupathirage, Keitaro Yoshida, Keisuke Yamamoto, Takuya Kakuki, Sumito Jitsukawa, Toyotaka Sato, Hirofumi Sawa, Yasuko Orba, Akihiko Sato, Takashi Kojima, Wataru Kamitani, Satoshi Ichikawa, Tetsuya Nosaka, Kenichi Takano, Shin-ichi Yokota, Noriko Ogasawara

1. Supplementary Figures

Supplementary Figure 1

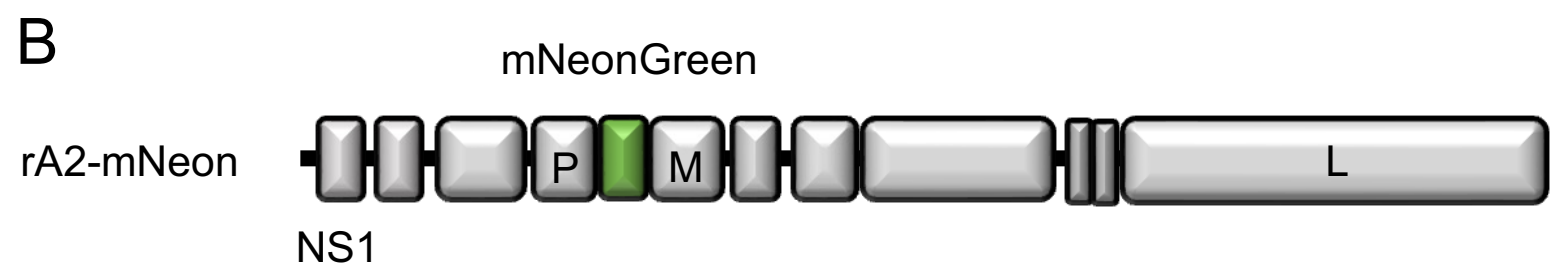

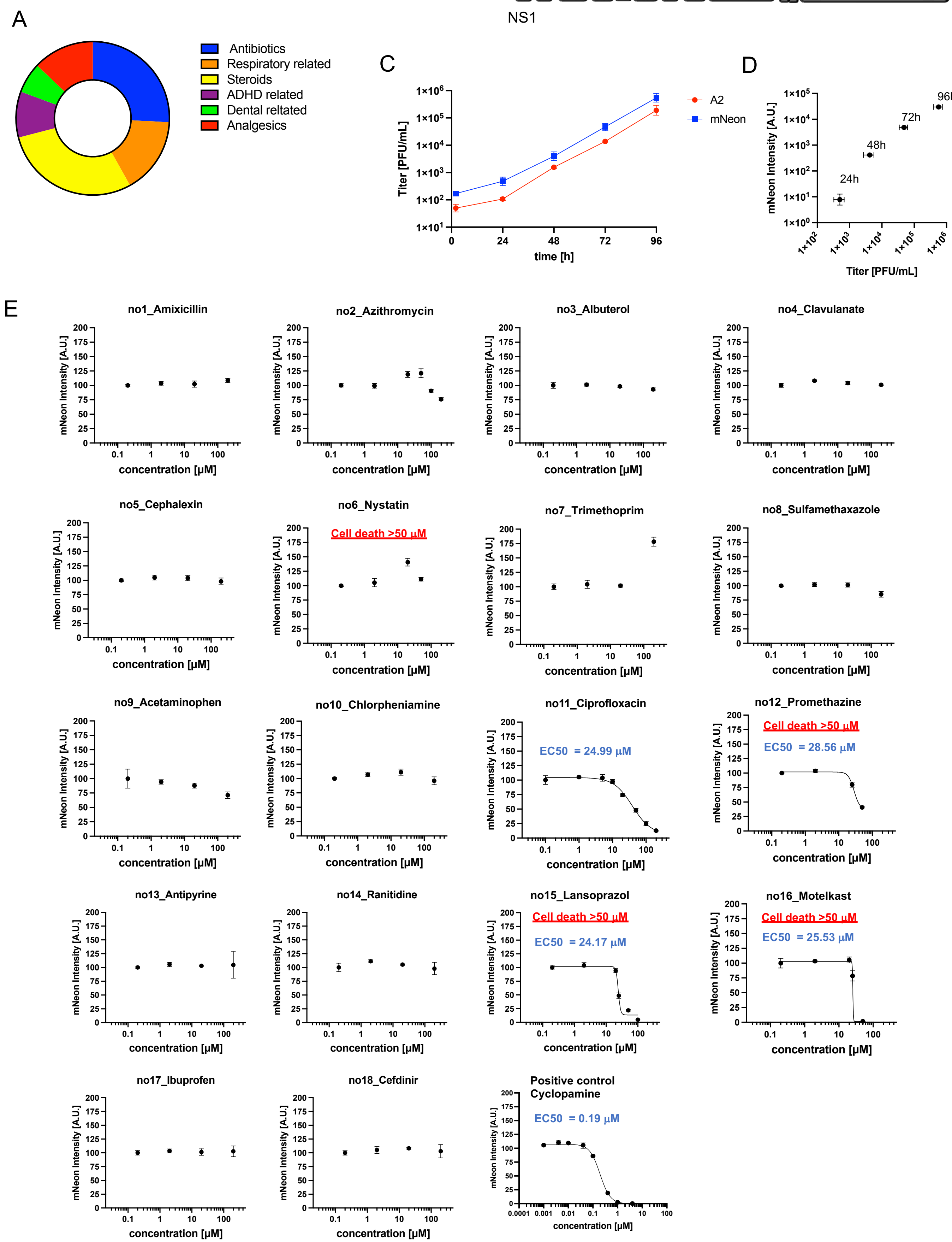

Supplementary Figure 1. Drug screening for anti-RSV activity using an mNeonGreen reporter virus.

(A) Pie chart showing the distribution of prescription drug categories in children under 17 years of age, based on data from reference (17). **(B)** Genome structure of recombinant RSV A2 expressing monomeric NeonGreen (mNeonGreen) inserted between the P and M genes (mNeon-A2 virus). **(C)** Replication kinetics of mNeon-A2 compared with wild-type RSV A2. HEp-2 cells were infected at an MOI of 0.005, and viral titers (PFU/mL) were determined at the indicated time points. **(D)** Correlation between reporter fluorescence intensity and viral titers. **(E)** Screening results of candidate drugs for anti-RSV activity using the mNeon-A2 reporter assay. Briefly, HEp-2 cells were infected with mNeon-A2 at an MOI of 0.005. After 2 h of incubation, cells were washed four times with medium and then incubated with medium supplemented with the indicated drugs for 72 h. Cyclopamine was used as a positive control. After incubation, cells were washed twice with cold 1× PBS (−) and lysed with buffer containing 1% NP-40, 5% glycerol, 0.15 M NaCl, 2 mM EDTA-Na, and 50 mM HEPES-NaOH (pH 7.4). Fluorescence of the lysate was determined using a microplate reader. Red and blue annotations indicate cytotoxic concentrations and calculated half-maximal effective concentration (EC_{50}) values, respectively.

Supplementary Figure 2

A

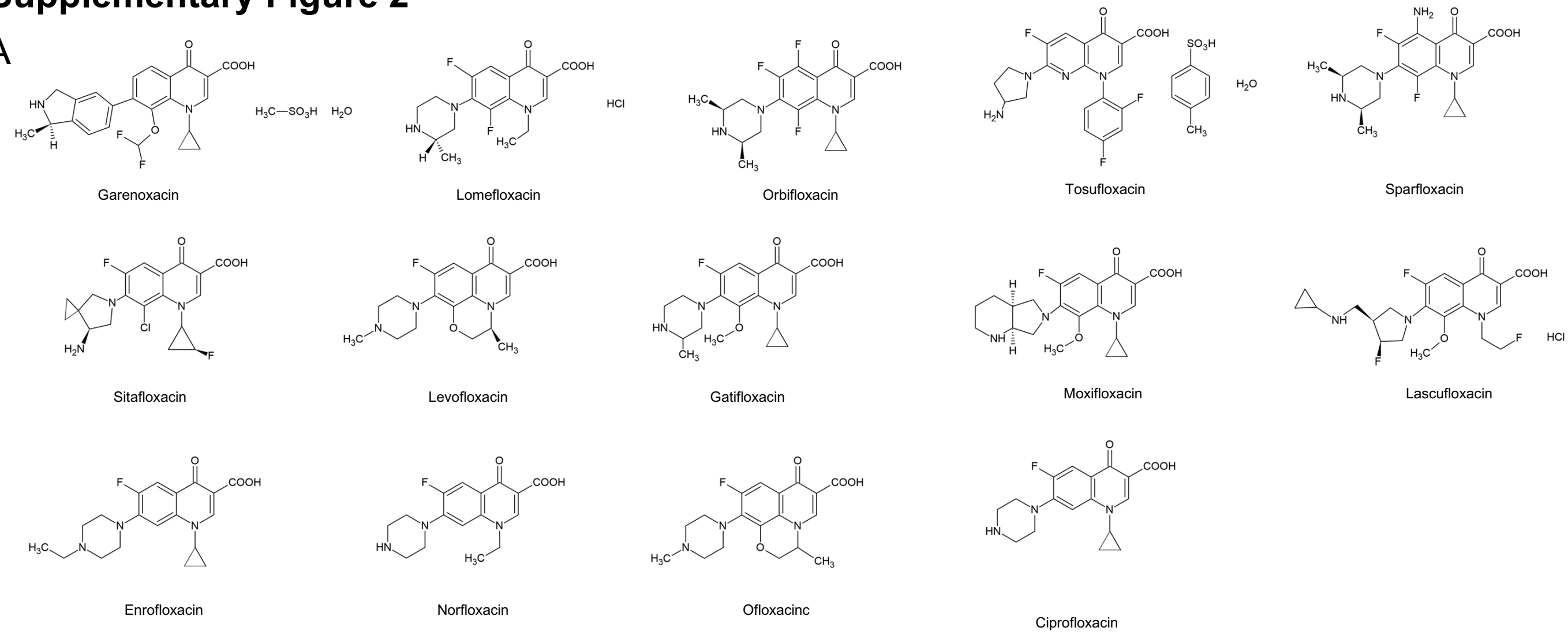

B

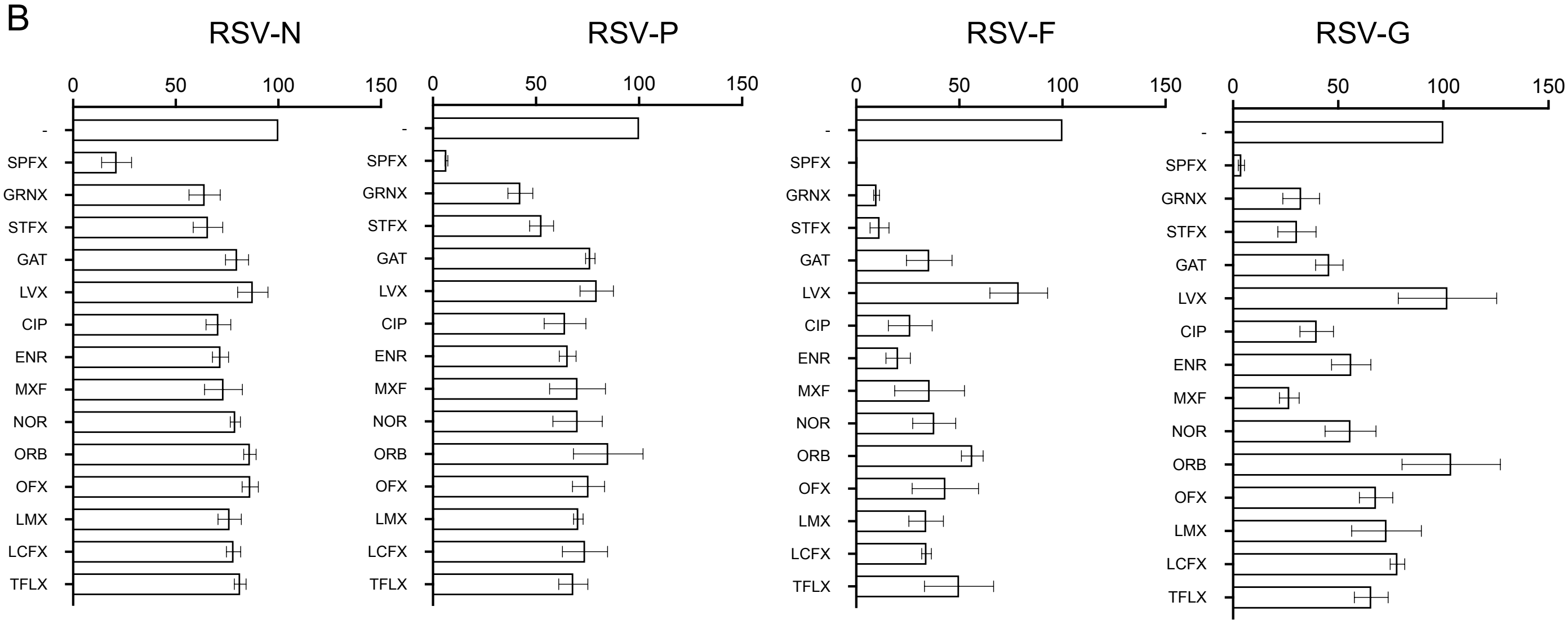

Supplementary Figure 2 Evaluation of fluoroquinolone derivatives and antiviral specificity. **(A)** Structural formulas of the fluoroquinolone antibiotics used in this study. **(B)** Quantification of RSV protein expression (N, P, F, and G) following fluoroquinolone treatment with the indicated fluoroquinolones. Protein levels were quantified from western blot analyses corresponding to Fig. 1E. Abbreviations of antibiotics appear in Table 1. Representative western blots are shown in Supplementary Fig. 3.

Supplementary Figure 3

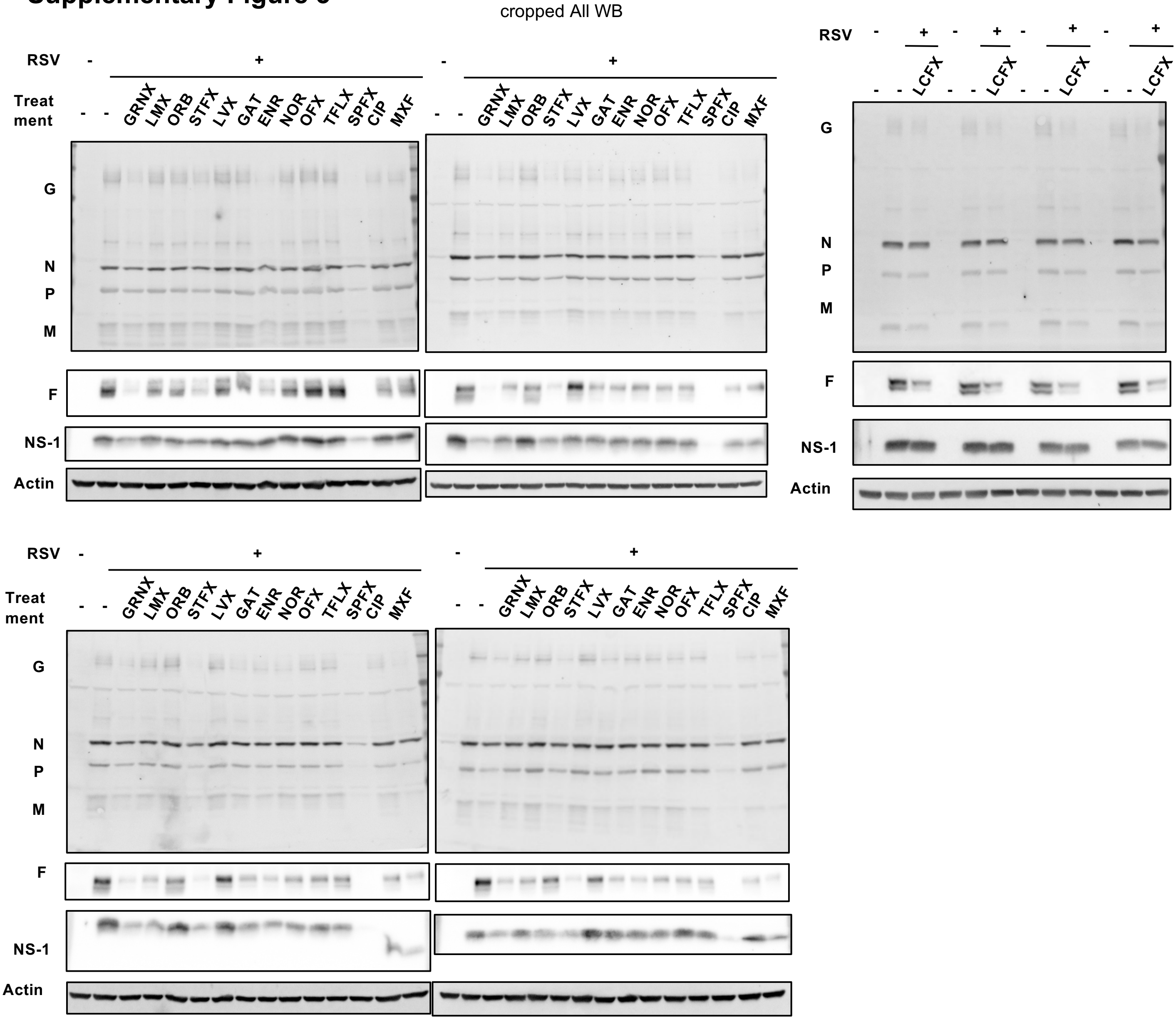

Supplementary Figure 3. Cropped western blots used for quantification of RSV proteins.

Cropped western blots corresponding to the quantification shown in Fig. 1E are presented. A549 cells were infected with RSV in the absence or presence of one of 14 fluoroquinolone antibiotics, or left untreated as a control. After 2 h incubation at 37 ° C, the inoculum was removed and replaced with fresh medium without drugs. Cell lysates were collected 24 h later.

Antibodies used for detection included anti-RSV, anti-RSV F, anti-RSV NS1, and anti-β-actin antibodies. Detailed antibody information is provided in Table 1. Band intensities were quantified using ImageJ. Abbreviations of antibiotics are listed in Table 1.

Supplementary Figure 4

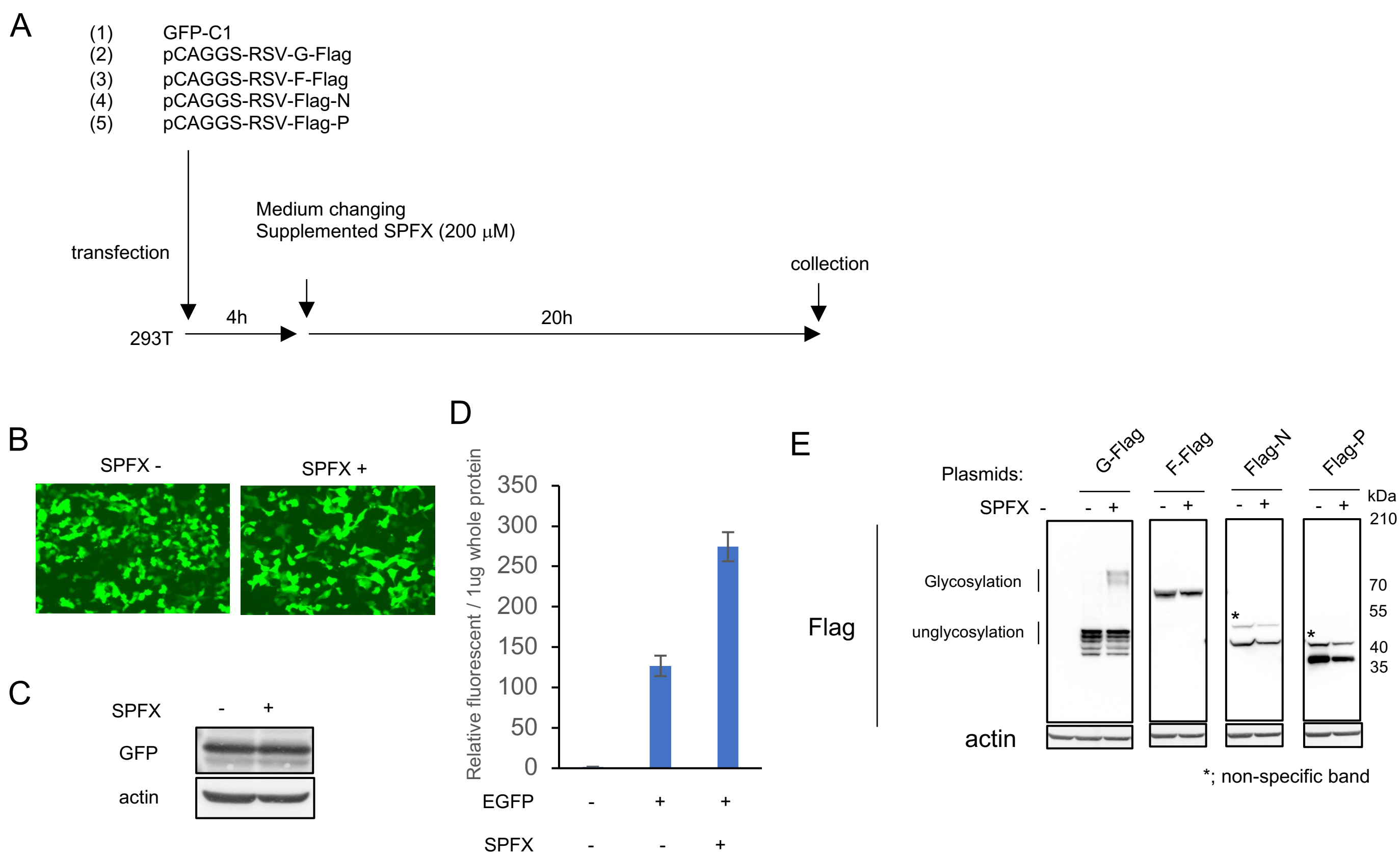

Supplementary Figure 4. SPFX does not impair cellular protein expression.

(A) Experimental scheme. GFP- or Flag-tagged RSV protein expression plasmids were transfected into HEK293T cells using Lipofectamine 3000. Four hours after transfection, the medium was replaced with fresh medium containing SPFX (200 μM), and cells were incubated for an additional 20 h before harvest. **(B)** Fluorescence images of GFP-transfected cells in the presence or absence of SPFX. **(C)** Western blot analysis of GFP expression in transfected cells with or without SPFX treatment. **(D)** Quantification of GFP fluorescence using a TECAN Infinite M200. Fluorescence intensity was normalized to total cellular protein. **(E)** Western blot analysis of Flag-tagged RSV proteins expressed from transfected plasmids in the presence or absence of SPFX.

Supplementary Figure 5

EC_{50}	JEV (ssRNA+)	ZIKV (ssRNA+)	YFV (ssRNA+)
SPFX	>283 uM	>283 uM	>283 uM
ORB	>283 uM	200.7 uM	>283 uM
Ribavirin	42.1 uM	32.8 uM	>400 uM

Supplementary Figure 5. SPFX does not affect the replication of flaviviruses. The antiviral activities of SPFX, ORB, and ribavirin against representative flaviviruses were assessed using an MTT-based cytopathic effect inhibition assay, as previously described (Uemura et al., 2023, ref. 18). BHK-21 cells were infected with Japanese encephalitis virus (JEV; Beijing-1), Zika virus (ZIKV; MR766), or yellow fever virus (YFV; 17D-204), and treated with twofold serial dilutions of SPFX, ORB, or ribavirin in 96-well plates. EC_{50} values were calculated using the same curve-fitting procedure as that used for RSV.

Supplementary Figure 6

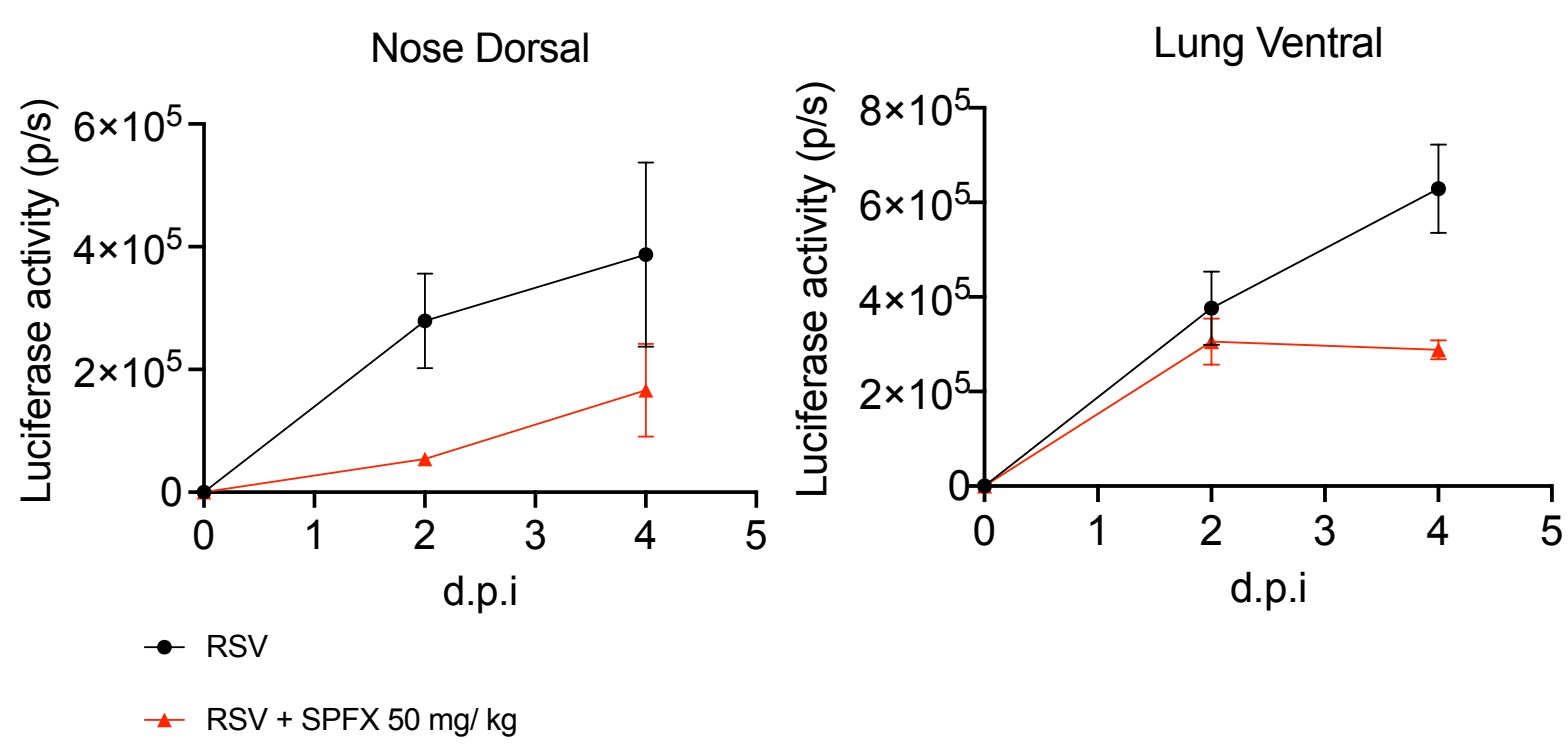

Supplementary Figure 6. Time course of in vivo luciferase activity using RSV-Luc5.
(A) Time course of luciferase activity in mice infected with RSV-Luc5. Bioluminescence signals were monitored longitudinally using an IVIS imaging system. Because panel (A) was intended to illustrate longitudinal viral dynamics using the RSV-Luc5 reporter as a surrogate for viral replication, only the RSV and RSV + SPFX groups were included. A subset of animals was used for serial imaging (RSV, n = 8; RSV + SPFX, n = 3). Repeated measurements obtained from the same animal across multiple time points were not treated as independent biological replicates.

Supplementary Figure 7

A. Ventral

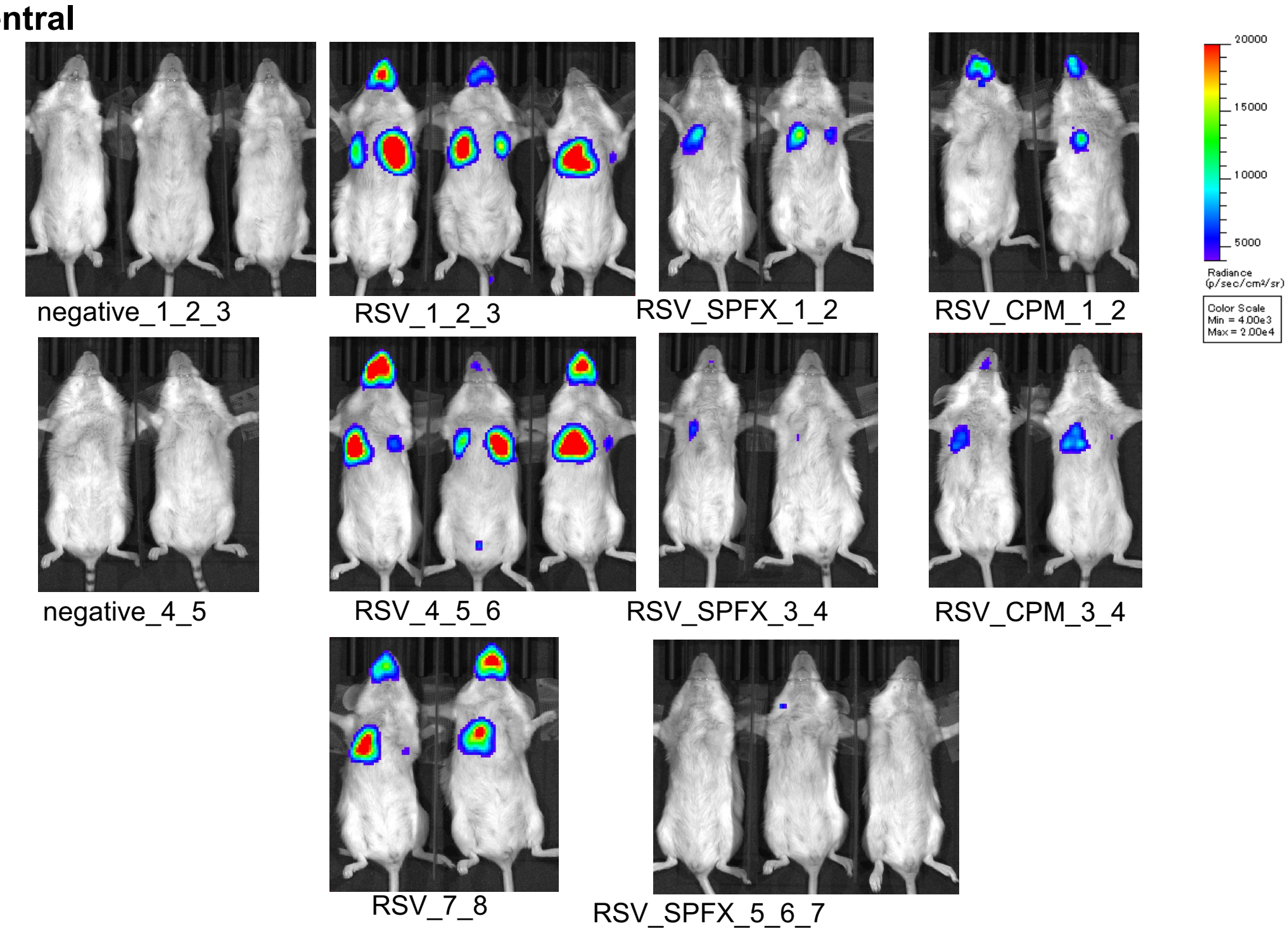

B. Dorsal

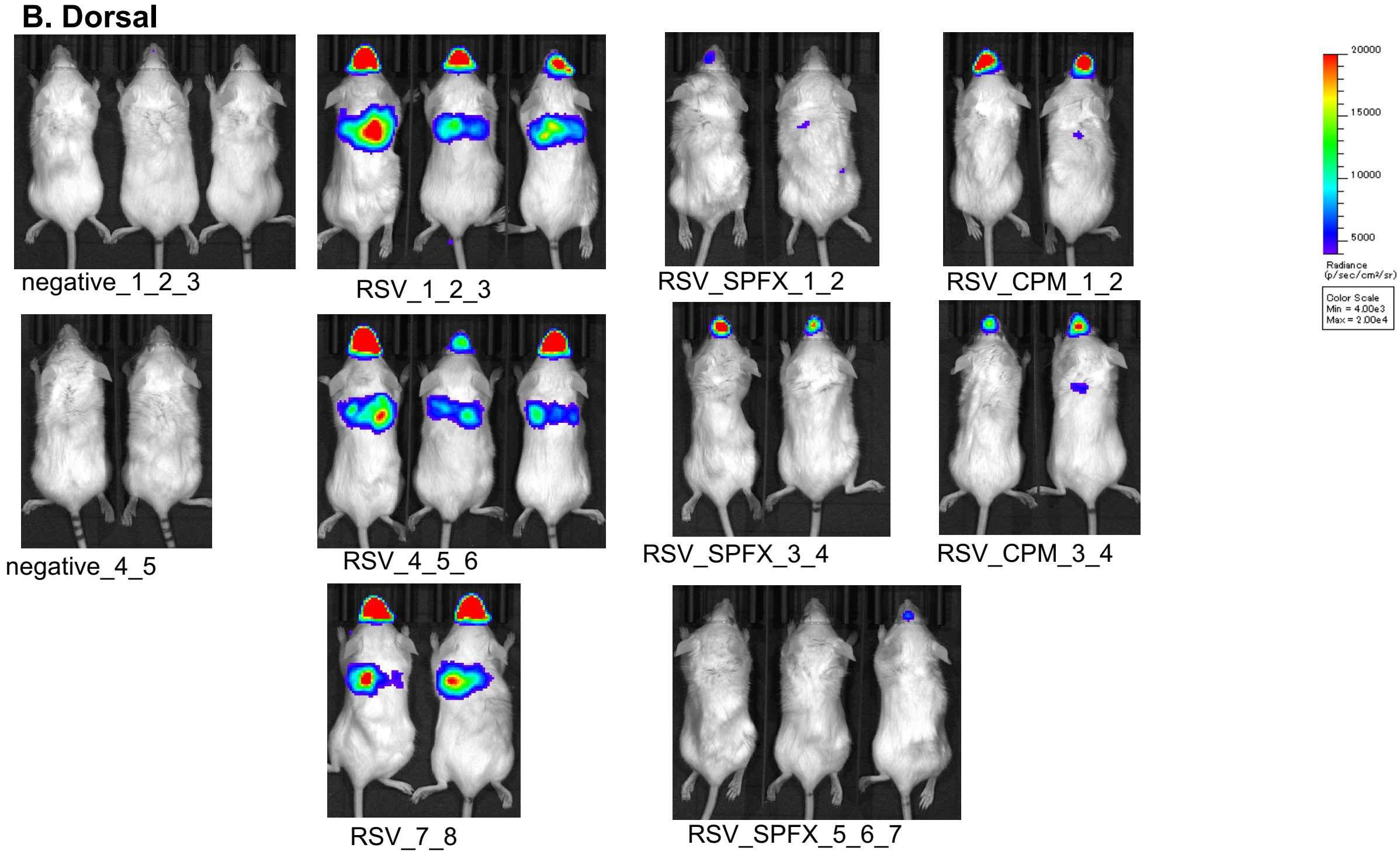

Supplementary Figure 7. Representative IVIS bioluminescence images of mice infected RSV-Luc5. Experimental groups were as follows: Group 1 (n = 5), uninfected controls; Group 2 (n = 8), RSV-Luc5 only (1 $\times$ 10^5 PFU/mouse); Group 3 (n = 7), RSV-Luc5 + SPFX; and Group 4 (n = 4), RSV-Luc5 + Cyclopamine (CPM).

Bioluminescence imaging was performed at 2 and 4 d.p.i. after administration of D-luciferin (75 mg/kg). Photon emission was captured using the IVIS imaging system. Representative ventral (A) and dorsal (B) views are shown.

Supplementary Figure 8

A. Weight

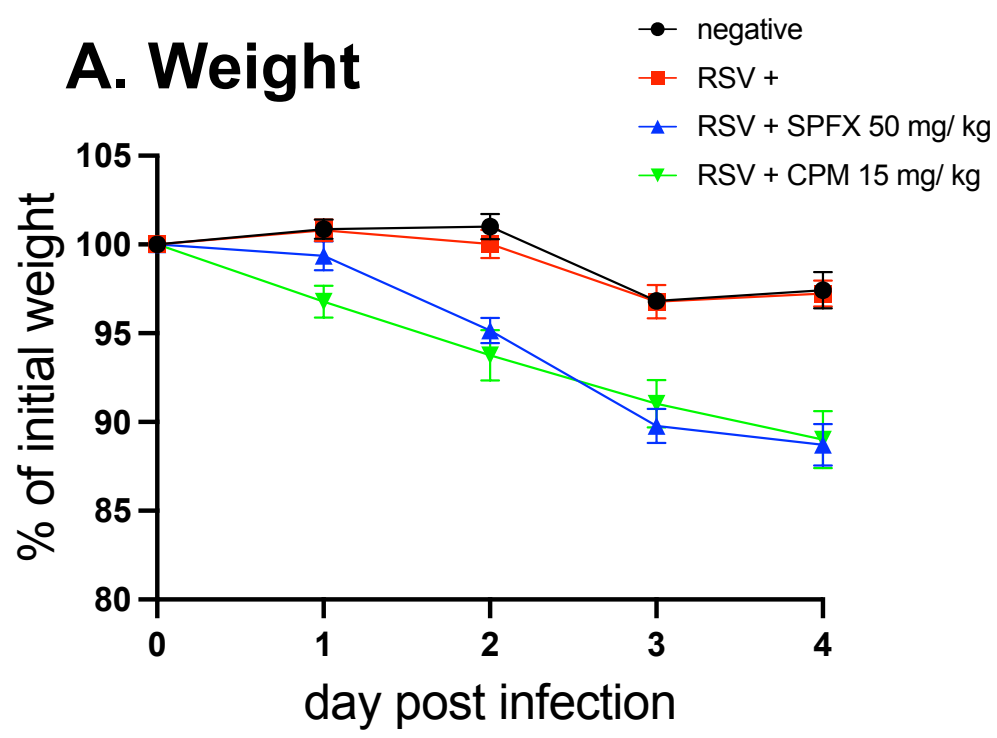

B. Ventral lung

NO.	negative	RSV	RSV_SPFX	RSV_CPM
1	1143	8130	2134	1144
2	876.4	4840	2721	1763
3	978.2	5480	1264	1729
4	782.2	4480	1025	2226
5	832.8	4900	986.3	
6		5980	1279	
7		4175	867.1	
8		4106		

C. Dosal Nose

NO.	negative	RSV	RSV_SPFX	RSV_CPM
1	1021	8131	1528	7998
2	1072	4842	1056	8067
3	1129	5484	4687	2694
4	933.6	4478	2563	4354
5	1083	4903	909.8	
6		5979	1184	
7		19020	1383	
8		19080		

D. Histopathological score

	Neutrophil accumulation	Degree of neutrophil infiltration (including without accumulation) 0-3	Sum of Neutrophil score	lymphocyte accumulation	total score
negative1	2	2	4	0	4
negative2	1	1	2	0	2
negative3	1	1	2	0	2
negative4	0	0	0	0	0
negative5	0	0	0	1	1
RSV1	2	2	4	0	4
RSV2	4	2	6	3	9
RSV3	0	1	1	4	5
RSV4	1	2	3	0	3
RSV5	4	3	7	4	11
RSV6	3	3	6	7	13
RSV7	1	1	2	36	38
RSV8	8	3	11	2	13
RSV_SPFX1	2	2	4	1	5
RSV_SPFX2	0	1	1	0	1
RSV_SPFX3	2	1	3	2	5
RSV_SPFX4	3	2	5	1	6
RSV_SPFX5	2	1	3	0	3

Supplementary Figure 8. Summary of in vivo infection dynamics and clinical parameters in the RSV-Luc5 mouse model. (A) Relative body-weight changes following infection. Experimental groups were: Group 1, uninfected control (n = 5); Group 2, RSV-Luc5 infection alone (1×10^5 PFU/mouse, n = 8); Group 3, RSV-Luc5 infection with SPFX treatment (50 mg/kg, n = 7); and Group 4, RSV-Luc5 infection with Cyclopamine (CPM) treatment (n = 4). Body weight was recorded daily and expressed as the percentage of initial body weight at day 0. **(B, C)** Quantification of luciferase activity from IVIS images using Living Image software (v4.8.2, PerkinElmer). Signals were measured in the ventral lung region (B) and dorsal nasal region (C) and expressed as photons per second (p/s). Data include infected groups only (RSV, n = 8; RSV + SPFX, n = 7; RSV + CPM, n = 4). **(D)** Summary of histopathological scoring of lung inflammation. Neutrophil or lymphocyte clusters were defined as aggregates of ≥10 cells. For neutrophil infiltration, level 2 indicates sporadic neutrophils within the field, whereas level 1 indicates the presence of at least one neutrophil. The combined inflammatory score is presented as a scatter plot in Fig. 2E.

Supplementary Figure 9

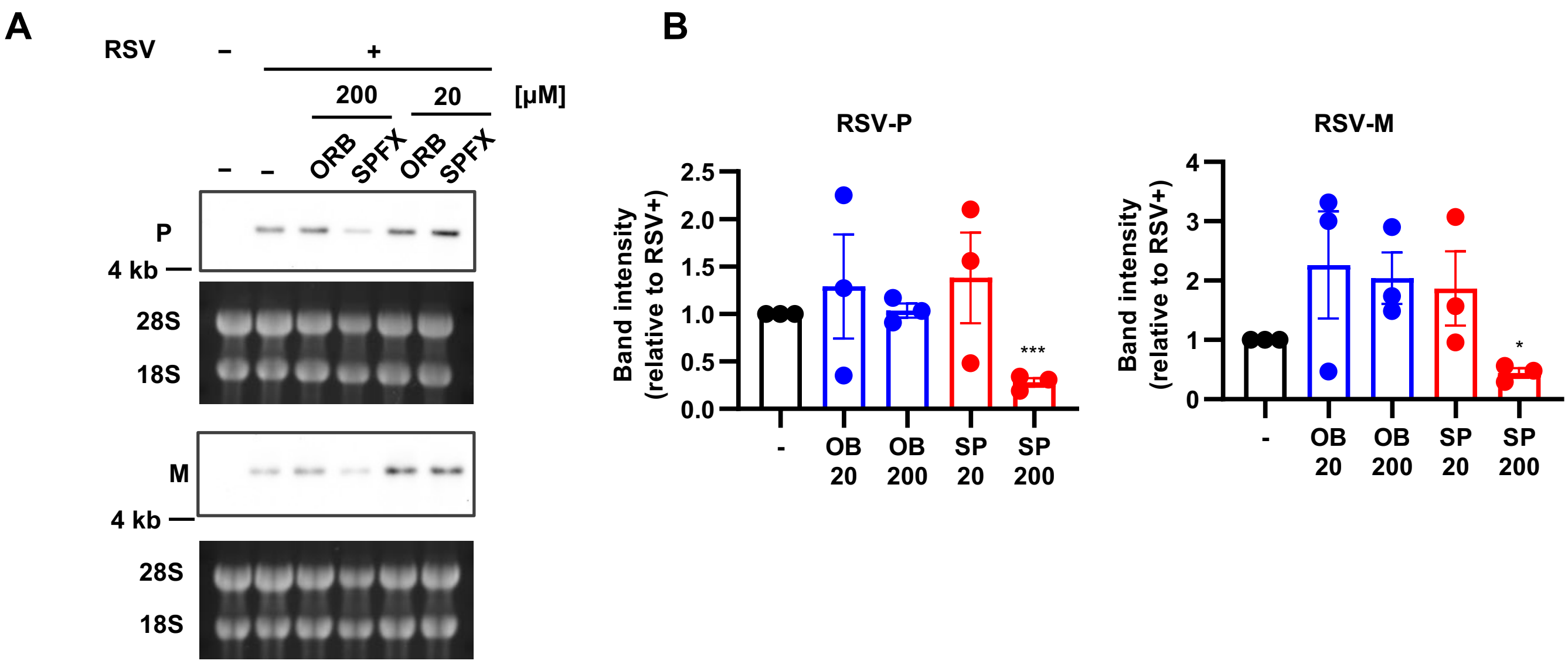

Supplementary Figure 9. Effects of ORB and SPFX on RSV mRNA levels assessed by Northern blotting.

(A) Representative Northern blots of RSV P and M transcripts following treatment with ORB (20 or 200 μM) or SPFX (20 or 200 μM). Molecular sizes are indicated to the left of each blot. Northern blots of 18S or 28S rRNA used as loading controls are shown below. **(B)** Quantification of RSV P and M transcript levels. Band intensities were determined by densitometric analysis using ImageJ (n = 3). *$p < 0.05$, **$p < 0.01$ versus untreated control by repeated-measures one-way ANOVA with Dunnett's multiple-comparisons test.

Supplementary Figure 10

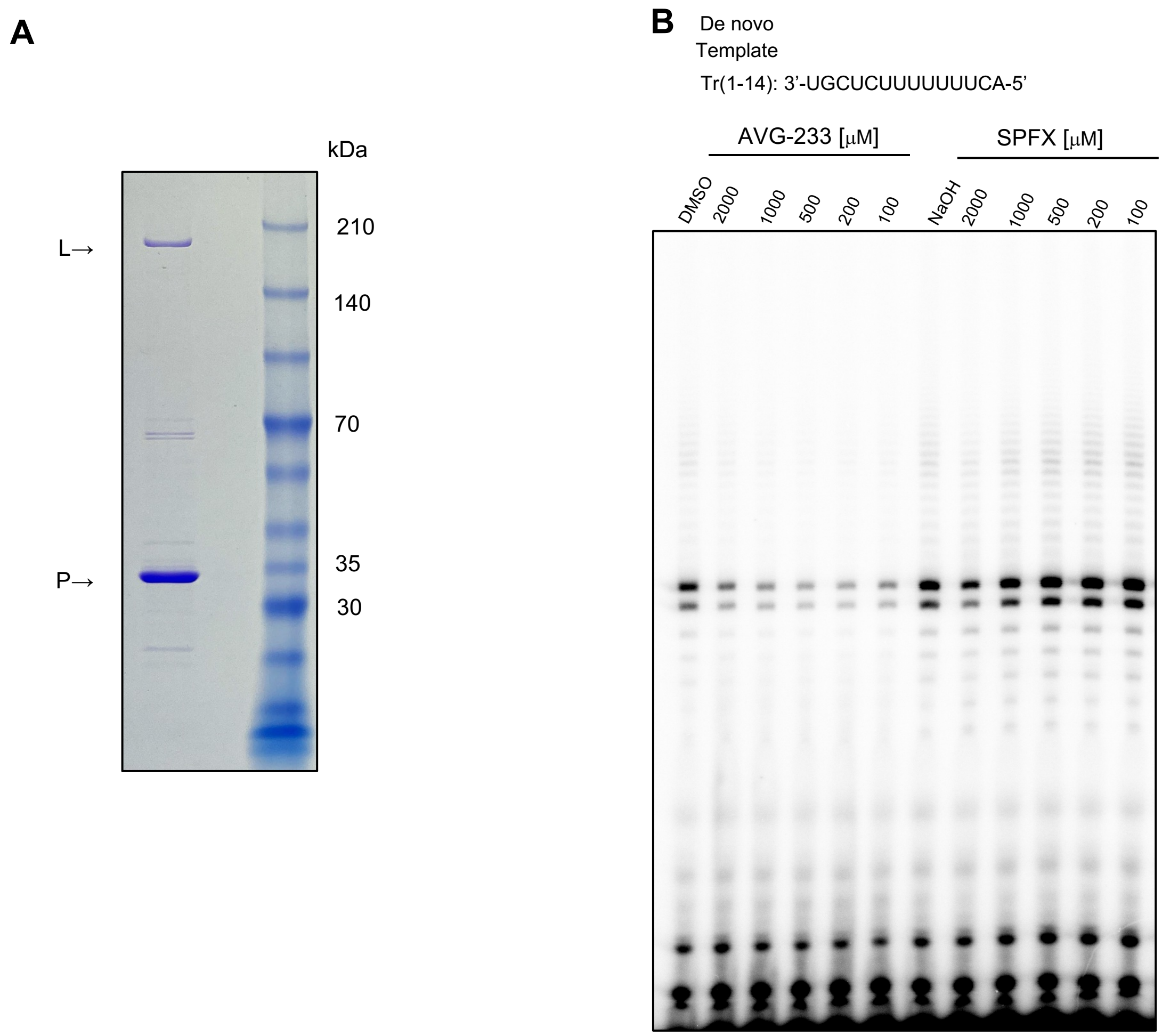

Supplementary Figure 10. Purified RSV L–P complex and in vitro RNA synthesis assays (de novo synthesis).

(A) SDS–polyacrylamide gel electrophoresis (SDS–PAGE) showing the quality and purity of the purified RSV L–P complex. Two micrograms of protein were loaded per lane. **(B)** Representative autoradiogram of in vitro RSV RNA-dependent RNA polymerase (RdRp) assays measuring de novo RNA synthesis from the promoter template in the presence of AVG-233 (0–2000 μM) or SPFX (0–2000 μM).

Supplementary Figure 11

A

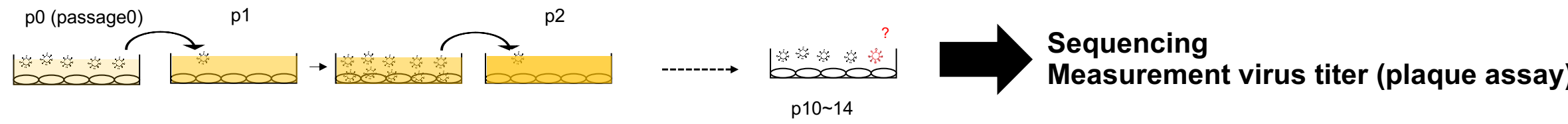

B C

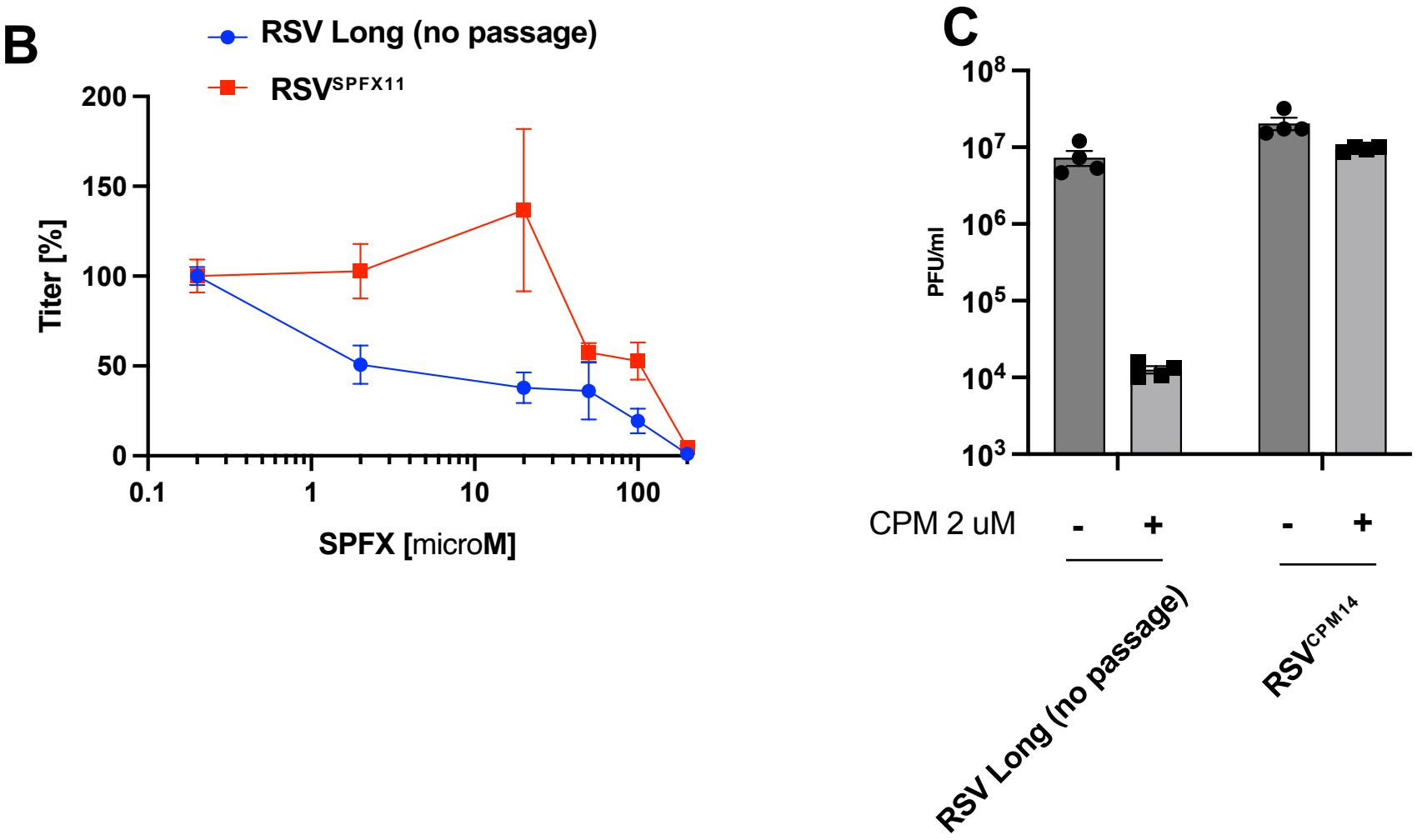

D

Virus	position	location	original AA	mutation AA
NaOHp6	63	before NS1		
	4041	between M and SH		
	5854	F	AAG(K)	AAT(D)
	5991	F	CTA(L)	CCA(P)
	6237	F	TTA(L)	TCA(S)
CPMp14	2322	between N and P		
	2632	P	AAT(D)	AGT(S)
	2637	P	TTT(F)	CTT(L)
	2654	P	AAA(K)	AAC(D)
	3177	between P and M		
	3247	between P and M		
	3717	M	GTA(V)	ATA(I)
	4880	G	GCA(A)	GTA(V)
	5406	G	AAA(K)	AAT(D)
	8004	M2_1/2	TAT(Y)	CAT(H)
	8036	M2_1/2	CAA(Q)	CAT(H)
	8043	M2_1/2	CAT(H)	AAT(D)
	8073	M2_1/2	TTG(L)	GTG(V)
ORBp11	40	before NS1		
	11573	L	GAT(D)	GGT(G)
SPFXp11	10655	L	CTG(L)	CAG(Q)
	11224	L	AGT(S)	GGT(G)
	11889	L	TTT(F)	TTG(L)

E

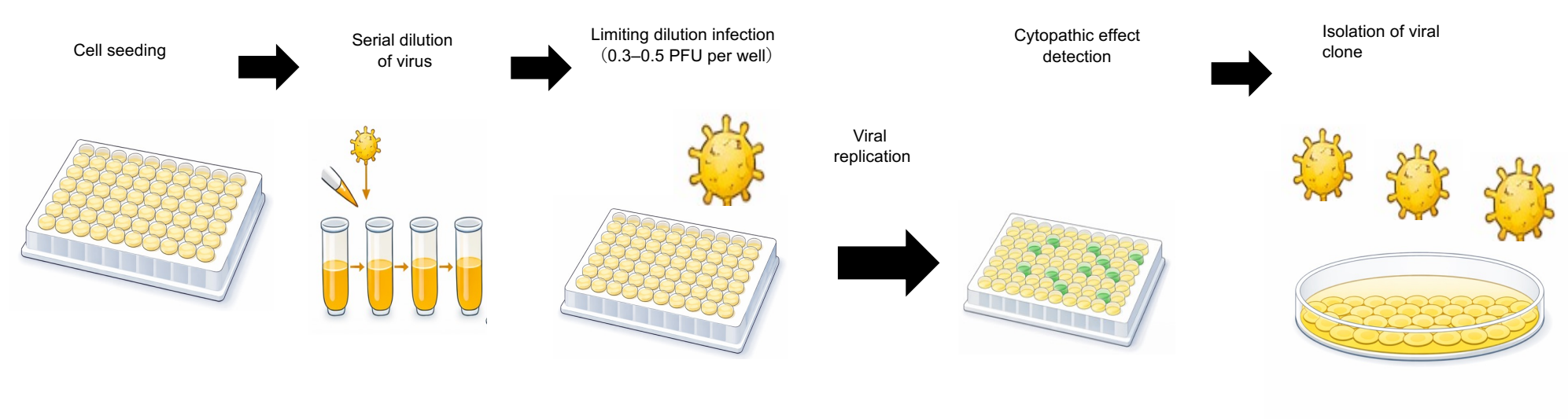

F G

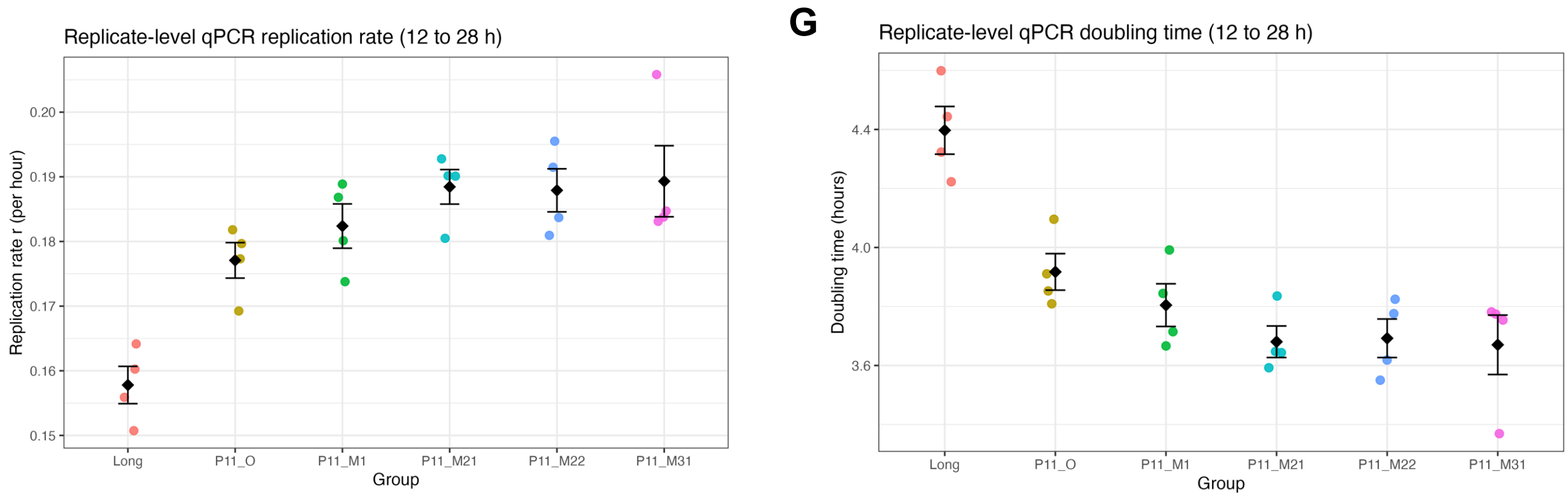

Supplementary Figure 11. Attempt to generate SPFX-resistant RSV mutants.

(A) Schematic of serial passage used to select drug-resistant RSV mutants. Virus was sequentially passaged in the presence of increasing concentrations of SPFX (20–80 μM), ORB (20–160 μM), or cyclopamine (CPM; 0.08–1 μM), and compared with control passages performed in the presence of NaOH vehicle. **(B)** Dose–response relationship of viral titers following SPFX selection. Viral titers were normalized to untreated conditions (set to 100%) and compared between parental virus (Long, no passage) and SPFX-passaged virus (RSV^{SPFX11}). **(C)** Viral titers of parental virus (Long, no passage) and CPM-passaged virus (RSV^{CPM14}) in the presence or absence of CPM (2 μM). Titers were determined by plaque assay in HEp-2 cells. **(D)** Nucleotide mutation sites and predicted amino acid substitutions identified by genome sequencing of RSV^{NaOH6}, RSV^{CPM14}, RSV^{ORB11}, and RSV^{SPFX11} relative to the reference RSV A2 strain (AY911262). **(E)** Schematic overview of limiting dilution cloning used to isolate monoclonal viral populations from SPFX-passaged virus. **(F)** Replicate-level qPCR-based replication rates (12-28 h post infection) for parental virus (Long), passaged population (P11_O), and representative monoclonal derivatives (P11_M1, P11_M21, P11_M22, and P11_M31). Replication rates (r) were calculated assuming exponential growth using $r = (\ln N_2 - \ln N_1)/(t_2 - t_1)$. Each dot represents an individual replicate; black diamonds indicate mean values with error bars showing SEM (n = 4). **(G)** Corresponding doubling times derived from qPCR measurements. Doubling time was calculated as $T_d = \ln(2)/r$.

Supplementary Figure 12

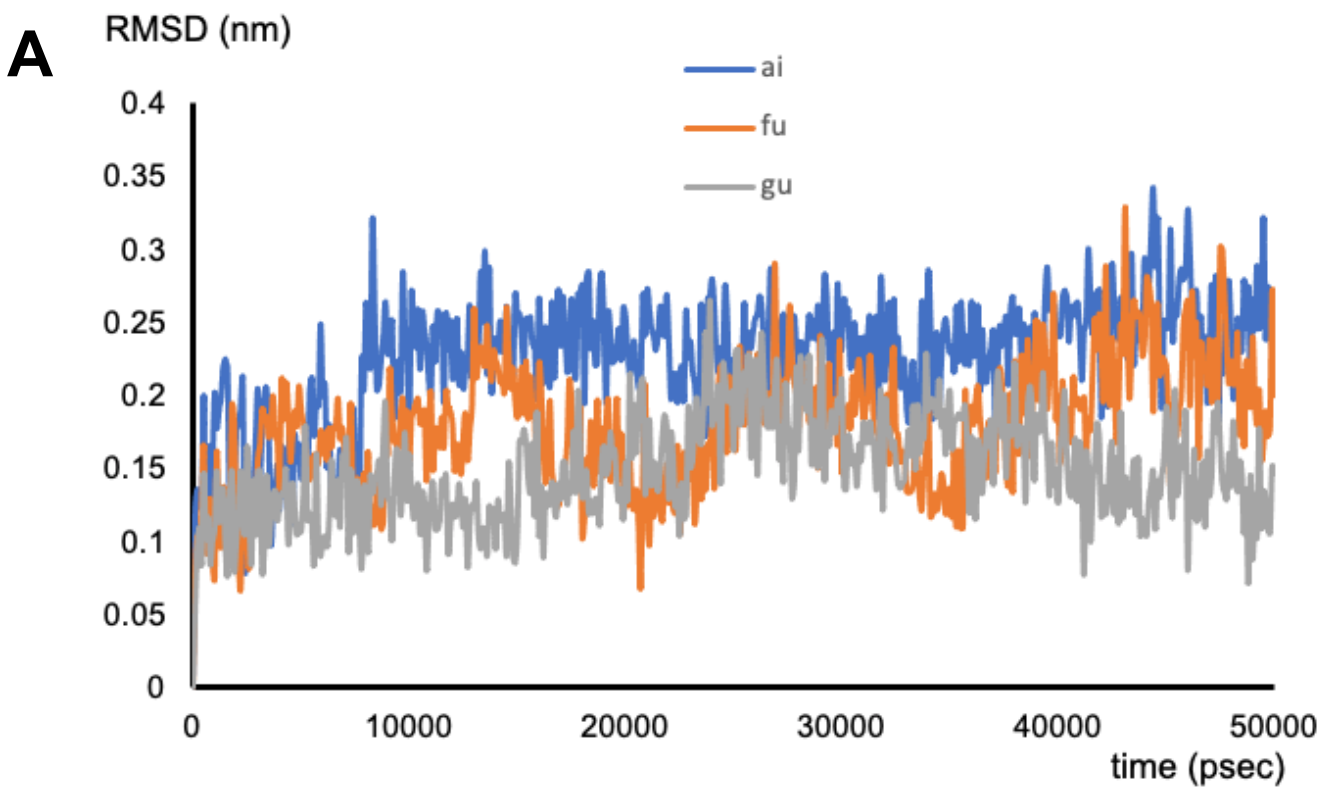

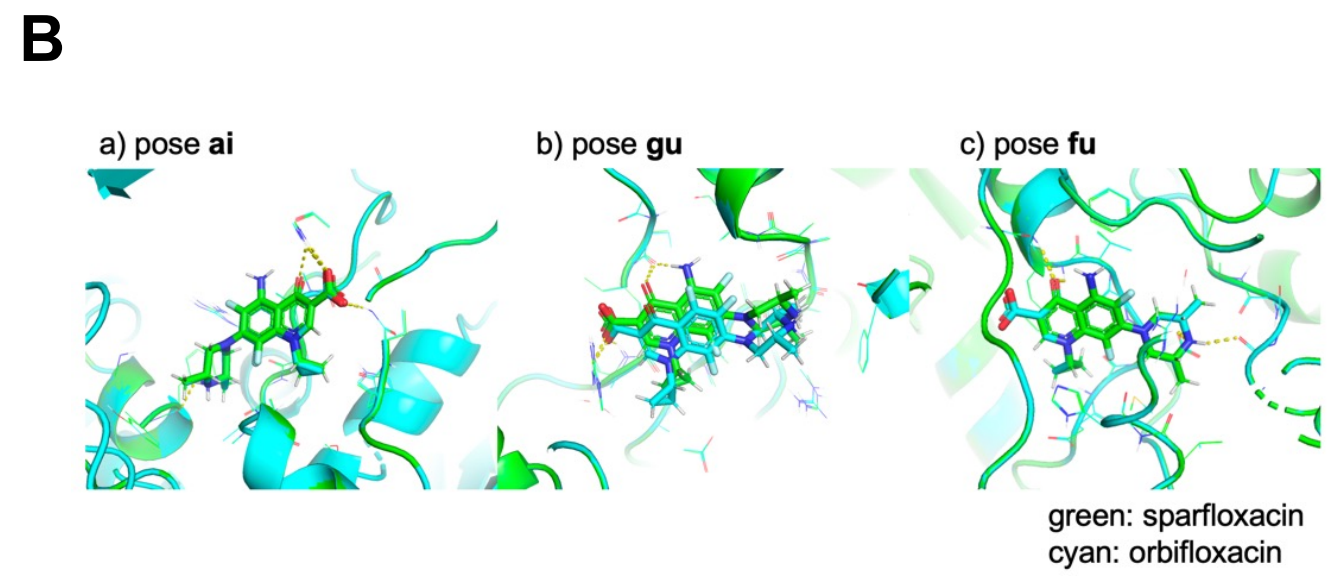

C

comp. (pose ID)	key interactions	deltaG bind
sparfloxacin (ai)	H-bond: R661, G1264, Y876	-40.14 KJ/mol
sparfloxacin (gu)	H-bond: R661, W1262	-35.03 KJ/mol
sparfloxacin (fu)	H-bond: G1218, Q1386, salt bridge: D1382	-26.72 k/Jmol
orbifloxacin (ai)	H-bond: R661, G1264, Y876	-41.29 KJ/mol
orbifloxacin (gu)	H-bond: R661	-32.93 KJ/mol
orbifloxacin (fu)	H-bond: G1218, Q1386, salt bridge: D1382	-31.41 KJ/mol

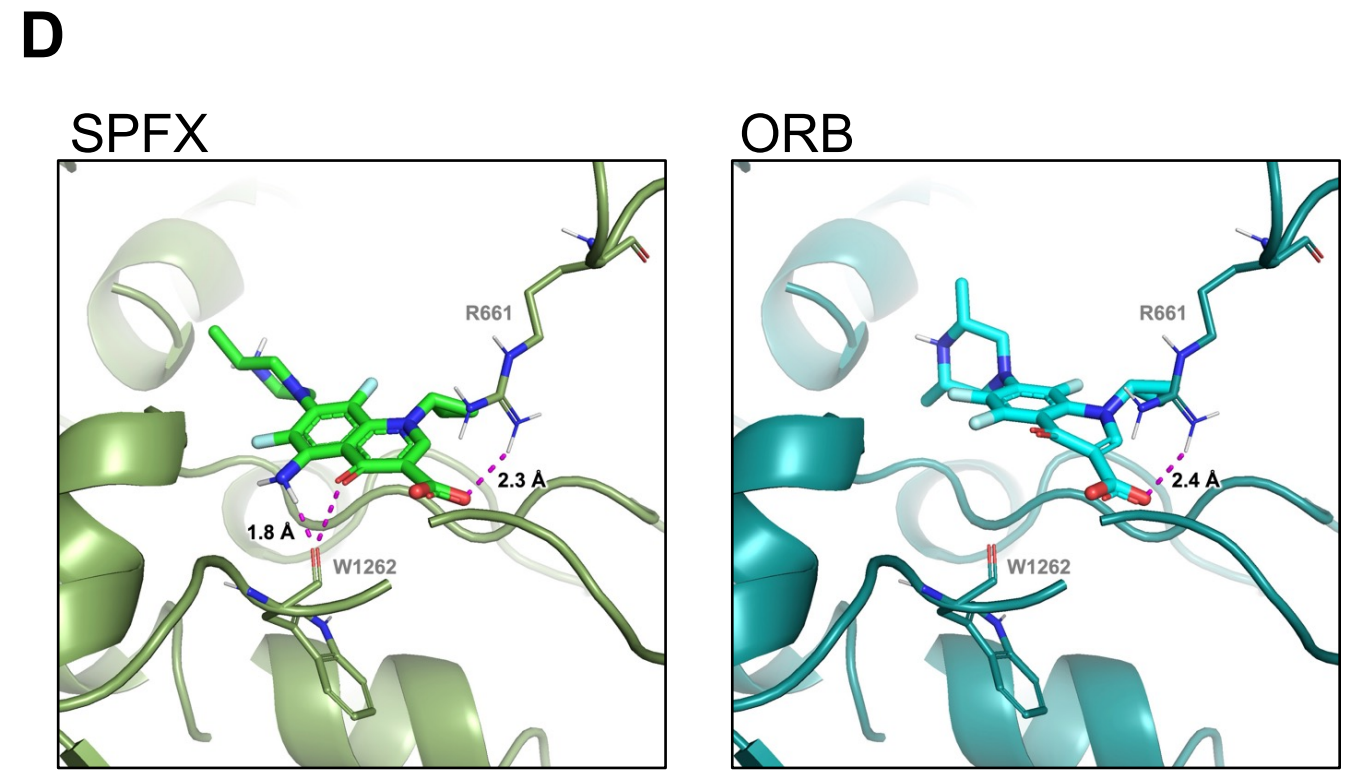

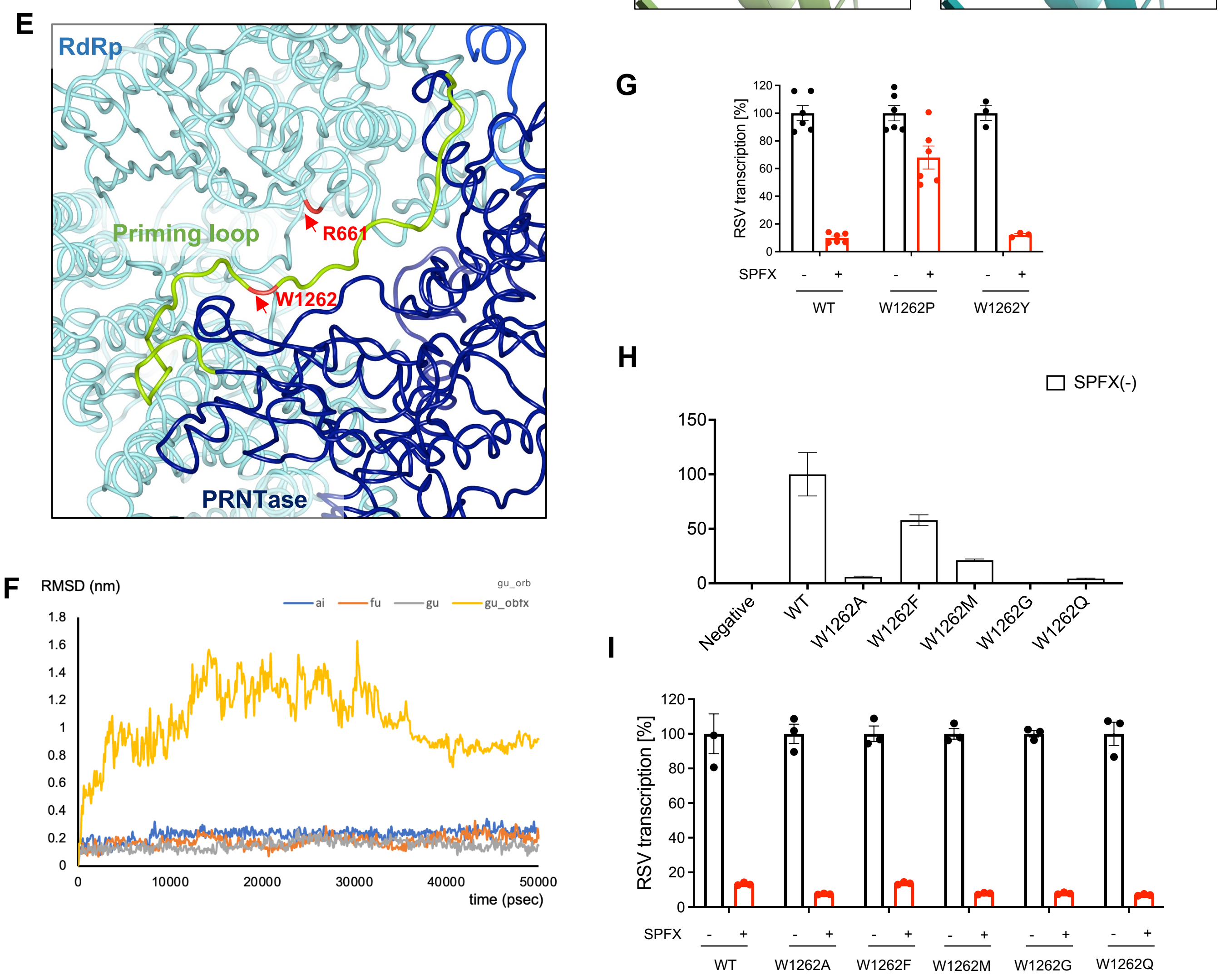

Supplementary Figure 12. Molecular docking simulations and functional validation of SPFX binding to RSV L protein.

(A) Trajectory root mean square deviation (RMSD) values of SPFX in three predicted docking poses (ai, blue; fu, orange; gu, gray). **(B)** Docking poses refined using the Glide program and further optimized by the Prime MM-GBSA method. SPFX is shown in green and ORB in cyan. **(C)** Predicted binding free energy values (ΔG_bind) for SPFX and ORB in the three docking poses. **(D)** Structural mapping of predicted SPFX- and ORB-binding residues on the RSV L protein. Right and left panels indicate SPFX and ORB, respectively. **(E)** Structural mapping of candidate SPFX-binding residues on the RSV L protein. Red indicates residues predicted by molecular docking. Light blue, dark blue, and light green denote the RdRp domain, PRNTase domain, and priming loop, respectively. **(F)** Trajectory RMSD values of ORB in the gu docking pose (gu_orb), compared with the SPFX trajectory shown in **(A)**.

(G, H, I) RSV minigenome assays using L protein variants at residue W1262. In **(G),** luciferase activity of each variant without SPFX was set to 100%. Red bars indicate the 200 μM SPFX treatment group. Data are shown as mean ± SD from n = 3–6 independent experiments.

In **(H)**, luciferase activity of wild-type L protein (WT) without SPFX was set to 100%. In **(I)**, luciferase activity of each variant without SPFX was set to 100%. Red bars indicate the 200 μM SPFX treatment group. Data are shown as mean ± standard deviation from three independent experiments.

Supplementary Figure 13

A

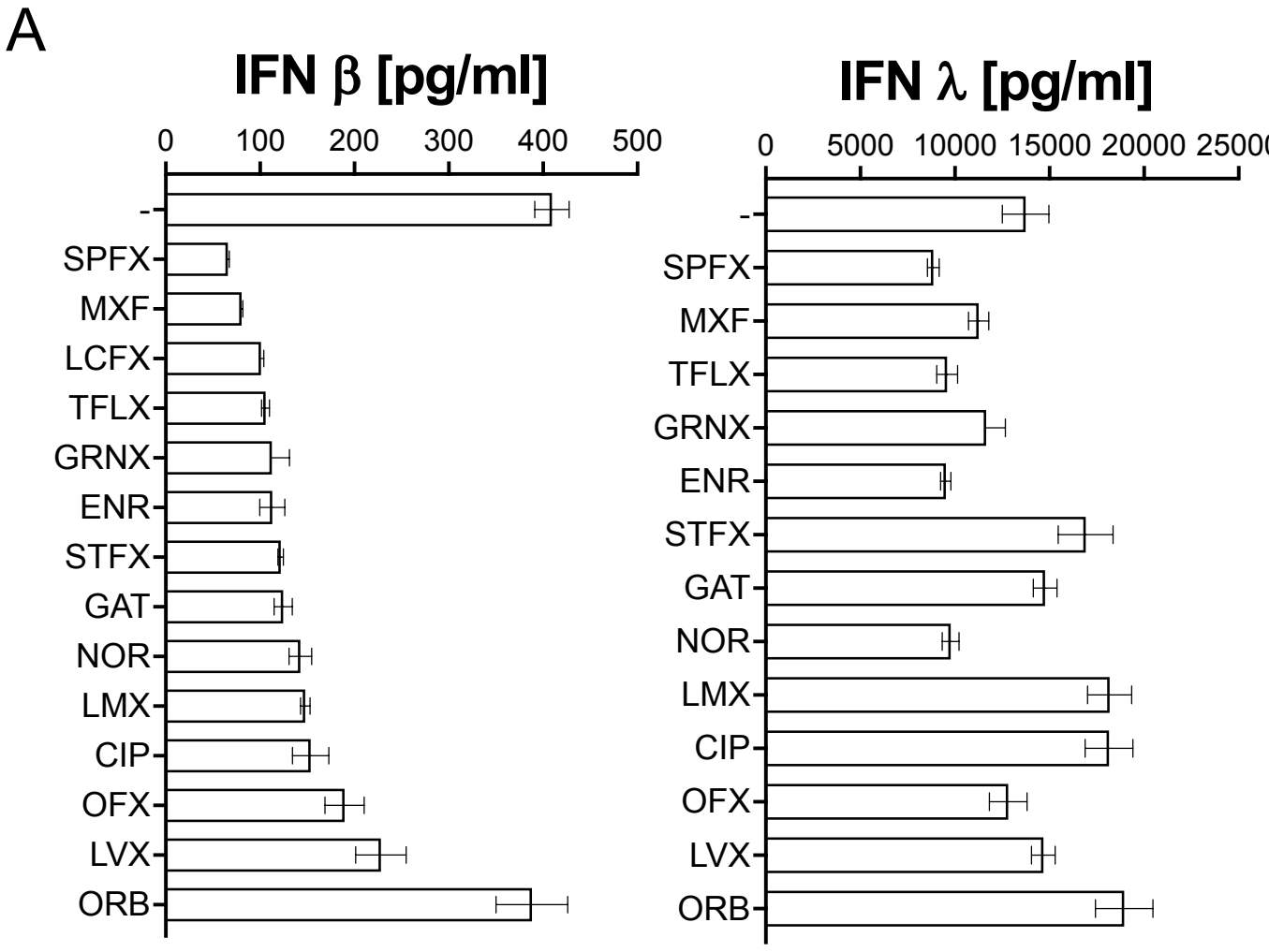

B

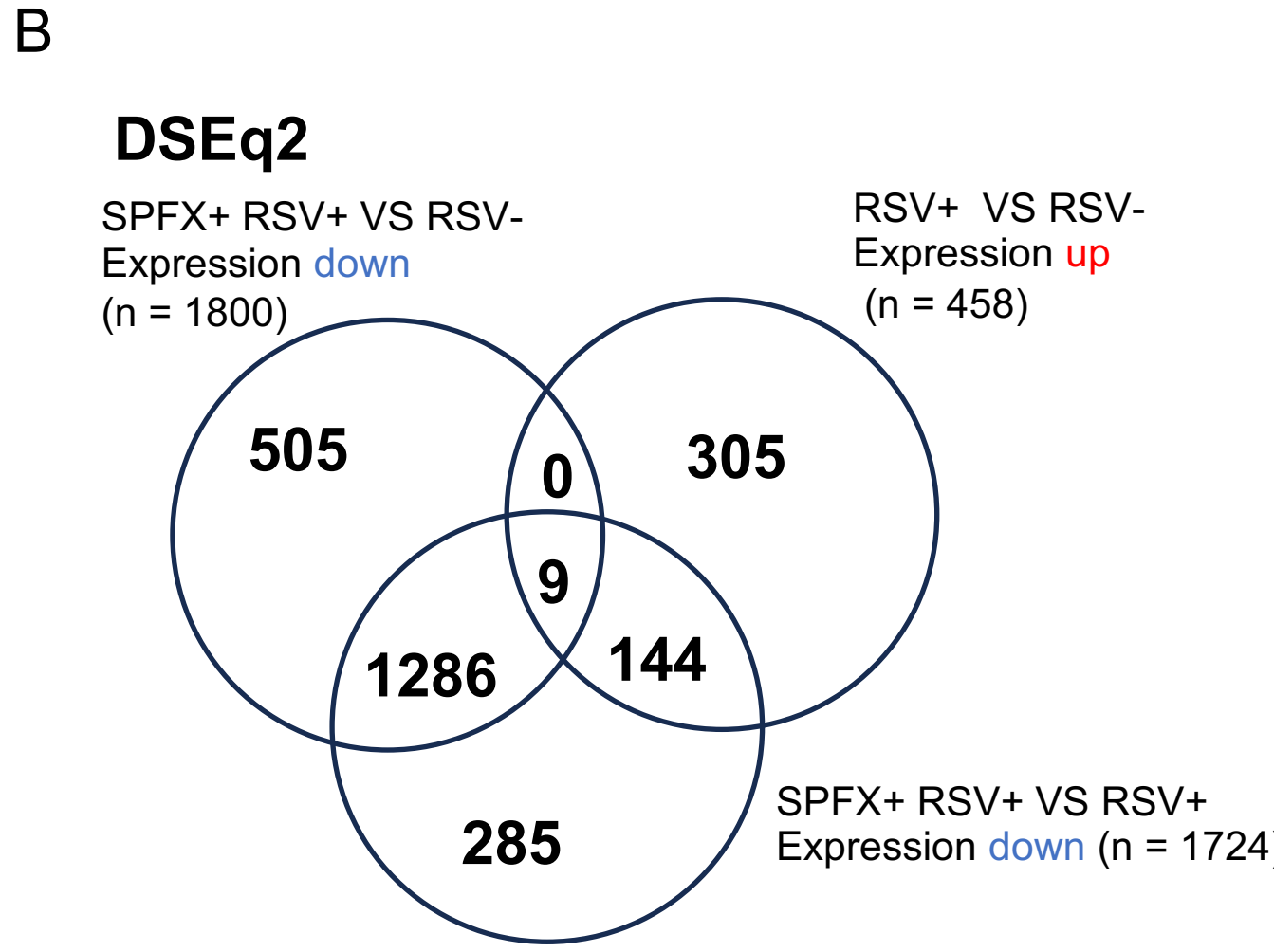

C

		RSV VS Non-infection up		SPFX VS Non-infection down		SPFX VS RSV down		ORB VS RSV	
		log2 FC	Adj.Pval	log2 FC	Adj.Pval	log2 FC	Adj.Pval	log2 FC	Adj.Pval
LimmaVoom	PRDM16	1.85433767	0.00776	1.23971266	0.000305	-4.8520848	0.000223	-	-
	FERMT3	1.86652876	0.0581	-1.9164214	0.0803	-3.7829501	0.00209	-	-
	CDK18	1.54264613	0.0957	-3.0022531	0.047	-4.5448992	0.00577	-	-
	STARD4	1.2702554	0.000637	-2.4015441	0.0000148	-3.6717995	0.0000013	-	-
	HSPA1A	1.75090768	0.000166	-1.2402923	0.00028	-2.9912	0.000000793	-	-
	ZBTB42	1.12331798	0.037	-1.0529859	0.0411	-2.1763039	0.000592	-	-
	MOV10	1.35952629	0.000781	-1.4320816	0.000432	-2.7916079	0.00000368	-	-
	TCAF2	1.41163637	0.055	-1.8238373	0.0456	-3.2354736	0.00185	-	-
	HSPA1B	1.62932356	0.000699	-1.151824	0.00122	-2.7811476	0.00000275	-	-
DESeq2	PRDM16	2.001969644	6.30E-05	-2.798462801	1.48E-04	-4.8004324	1.02E-11	-	-
	CDK18	1.788237806	7.17E-03	-2.858451057	1.87E-05	-4.6466889	8.56E-13	-	-
	STARD4	1.328027031	2.03E-09	-2.270977332	2.13E-24	-3.5990044	1.25E-60	-	-
	PAXIP1_AS2	1.116579124	3.30E-02	-1.067483713	2.05E-02	-2.1840628	0.000000555	-	-
	HSPA1A	1.814985738	3.04E-17	-1.055981517	9.00E-07	-2.8709673	1.32E-43	-	-
	MOV10	1.473286852	7.94E-09	-1.267078103	4.46E-07	-2.740365	1.74E-29	-	-
	GSDMD	1.149684762	5.29E-02	-1.740035512	2.42E-04	-2.8897203	4.03E-10	-	-
	SPOCD1	1.131465672	5.22E-02	-1.865965756	1.93E-04	-2.9974314	7.17E-10	-	-
	TCAF2	1.496117998	2.20E-02	-1.809868211	3.29E-03	-3.3059862	1.92E-08	-1.683507528	6.56E-02

D

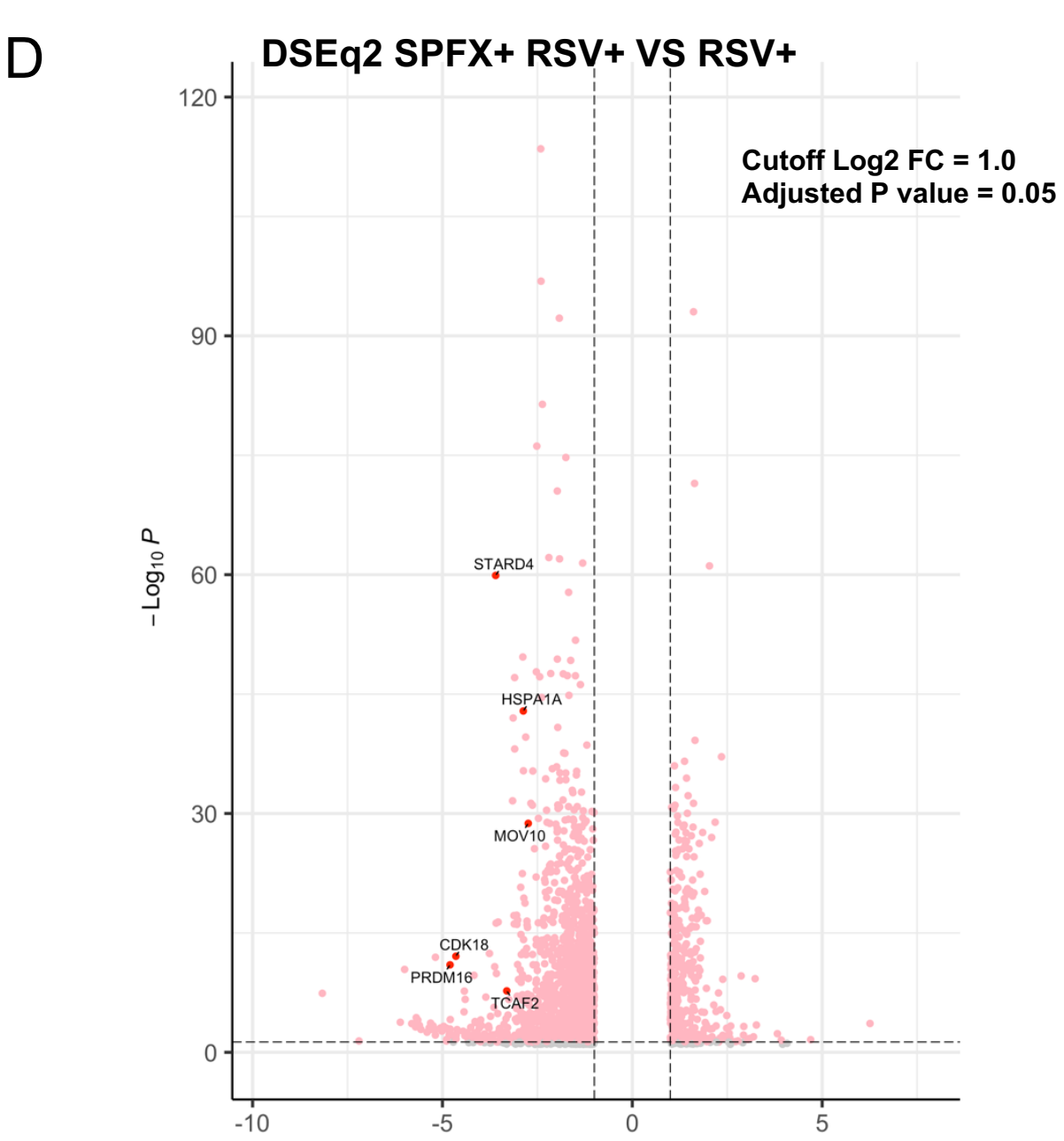

E

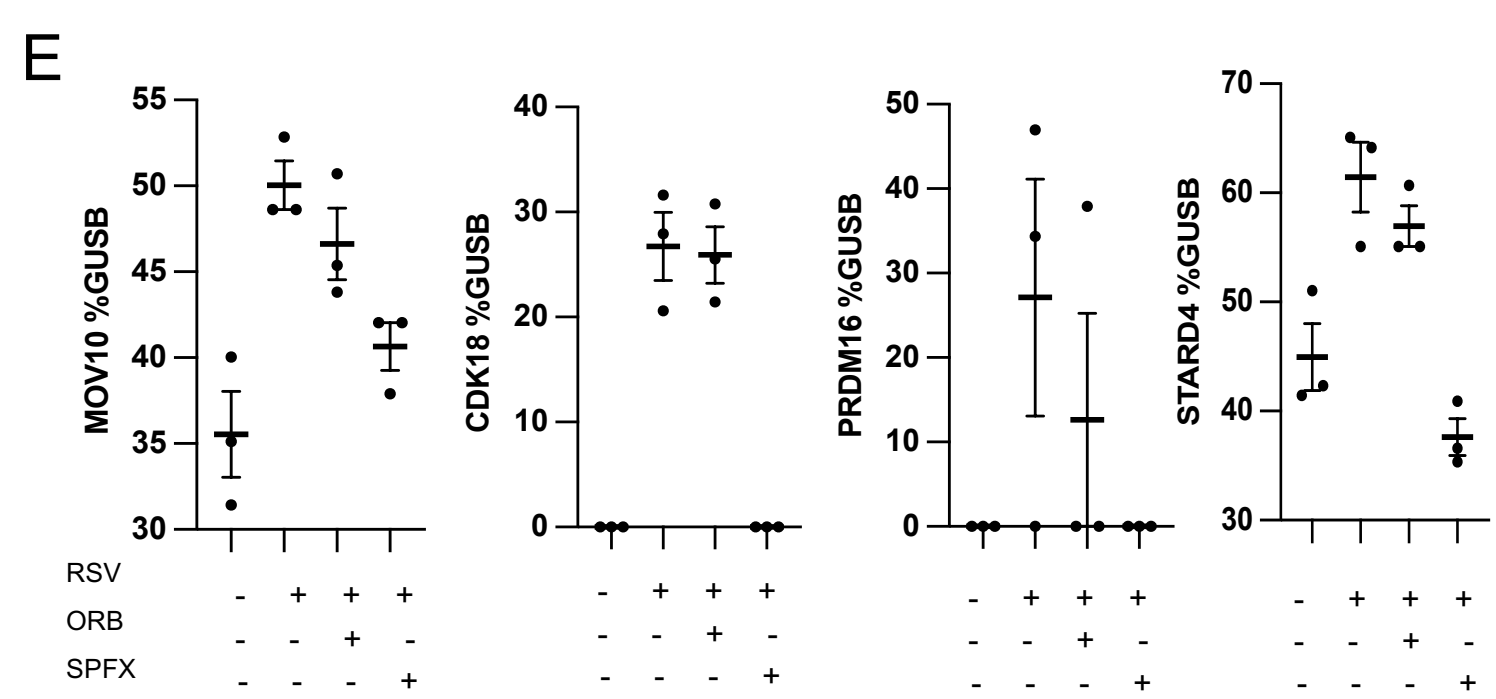

F

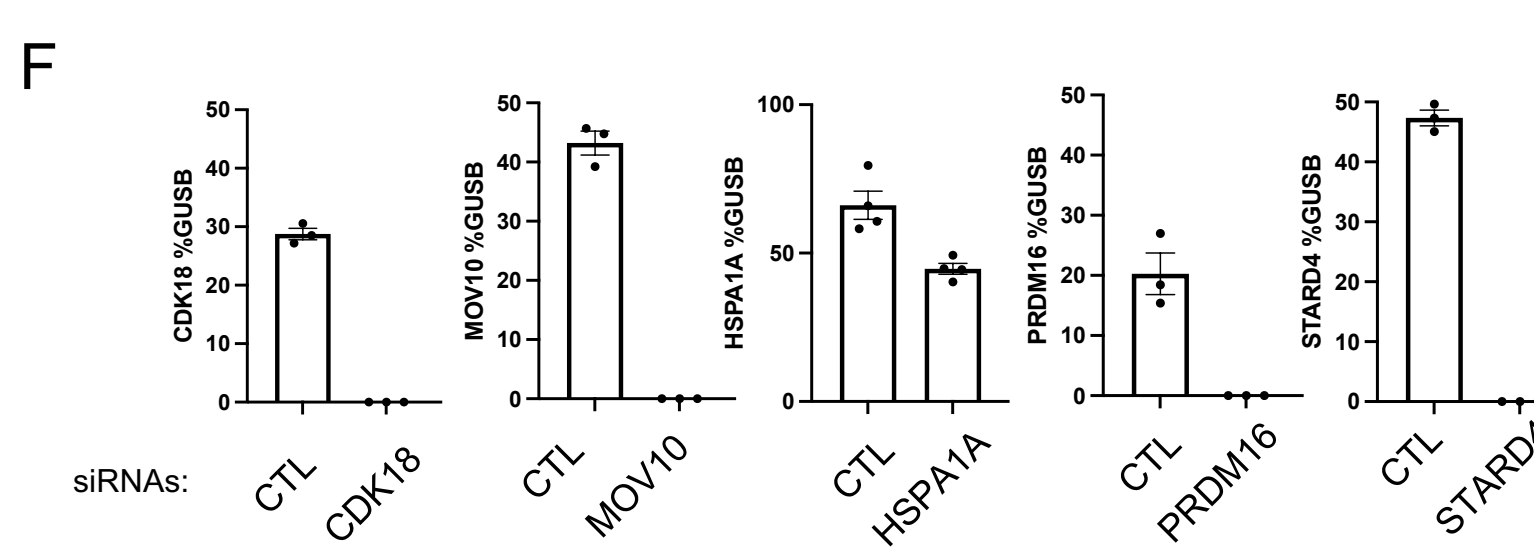

Supplementary Figure 13. Transcriptomic analysis of RSV-associated host factors specifically affected by SPFX treatment.

(A) Protein levels of interferon-β (left) and interferon-λ (right) measured by ELISA at 24 h post-infection (h.p.i.) with RSV in the presence or absence of the 14 fluoroquinolones tested. Abbreviations of fluoroquinolones are listed in Table 1. **(B)** Venn diagram showing candidate genes identified by bioinformatic analyses from Fig. 4B and C based on the DESeq2 algorithm. **(C)** List of $\log_2$ fold changes and adjusted p-values for the nine genes extracted by each analysis method. **(D)** List of $\log_2$ fold changes and adjusted p-values for the nine genes extracted by each analysis method. **(E)** Expression of four host genes (*CDK18*, *PRDM16*, *MOV10*, and *STRAD4*) during RSV infection and following SPFX or ORB treatment, measured by qPCR, normalized to *GUSB*, and expressed as percentage of control. **(F)** Knockdown efficiency of five candidate genes (*CDK18*, *MOV10*, *HSP70* [*HSPA1A]*, *PRDM16*, and *STRAD4*) following siRNA transfection, quantified by qPCR and normalized to *GUSB*.

Supplementary Figure 14

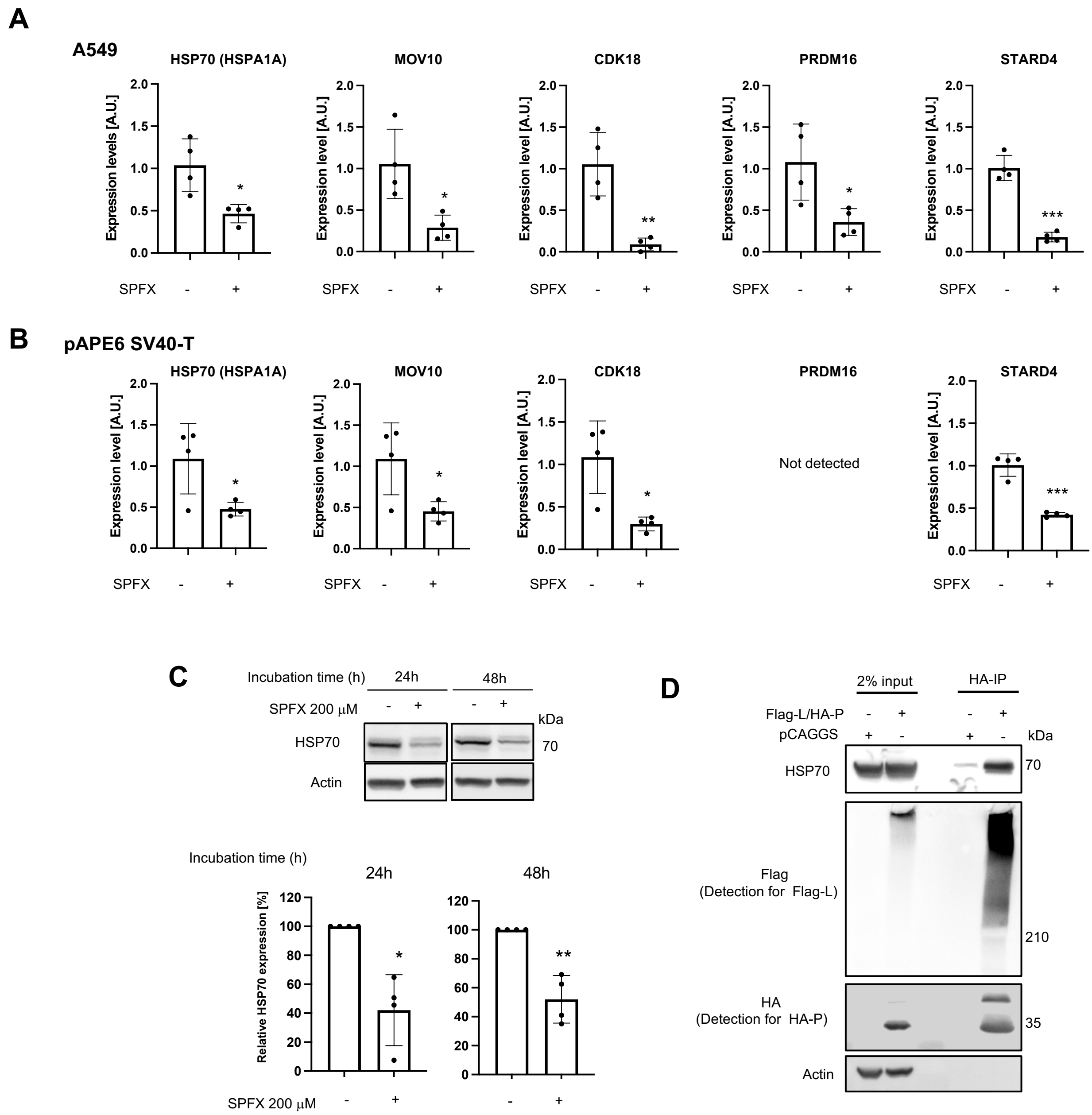

Supplementary Figure 14. Effects of SPFX on HSP70 expression and RSV P/L complexes. (A and B) Quantification of HSP70 mRNA expression in A549 cells **(A)** and pAPE6 SV40-T cells **(B)** treated with SPFX (200 µM) for 24 h. **(C)** Western blot analysis of HSP70 protein expression in A549 cells treated with SPFX (200 µM) for 24 or 48 h. **(D)** Co-immunoprecipitation analysis using an anti-HA antibody. Briefly, Flag-tagged RSV L- and HA-tagged RSV P-expression plasmids were transfected into Lenti-X 293T cells using PEI MAX. After 48 h, cells were crosslinked with 0.1% (v/v) formaldehyde for 10 min at 25 ° C, followed by quenching with 100 mM glycine-NaOH (pH 7.5) for 5 min. After washing with PBS, HA-tagged RSV P protein was immunoprecipitated using anti-HA Magarose beads (AlpaLifeBio, KTSM1335) for 1 h at 4 ° C. Proteins were eluted in 1× SDS sample buffer by boiling. Statistical significance was determined using an unpaired two-tailed Student's t-test versus SPFX-untreated controls. *p < 0.05, **p < 0.01, ***p < 0.001.

Supplementary Figure 15

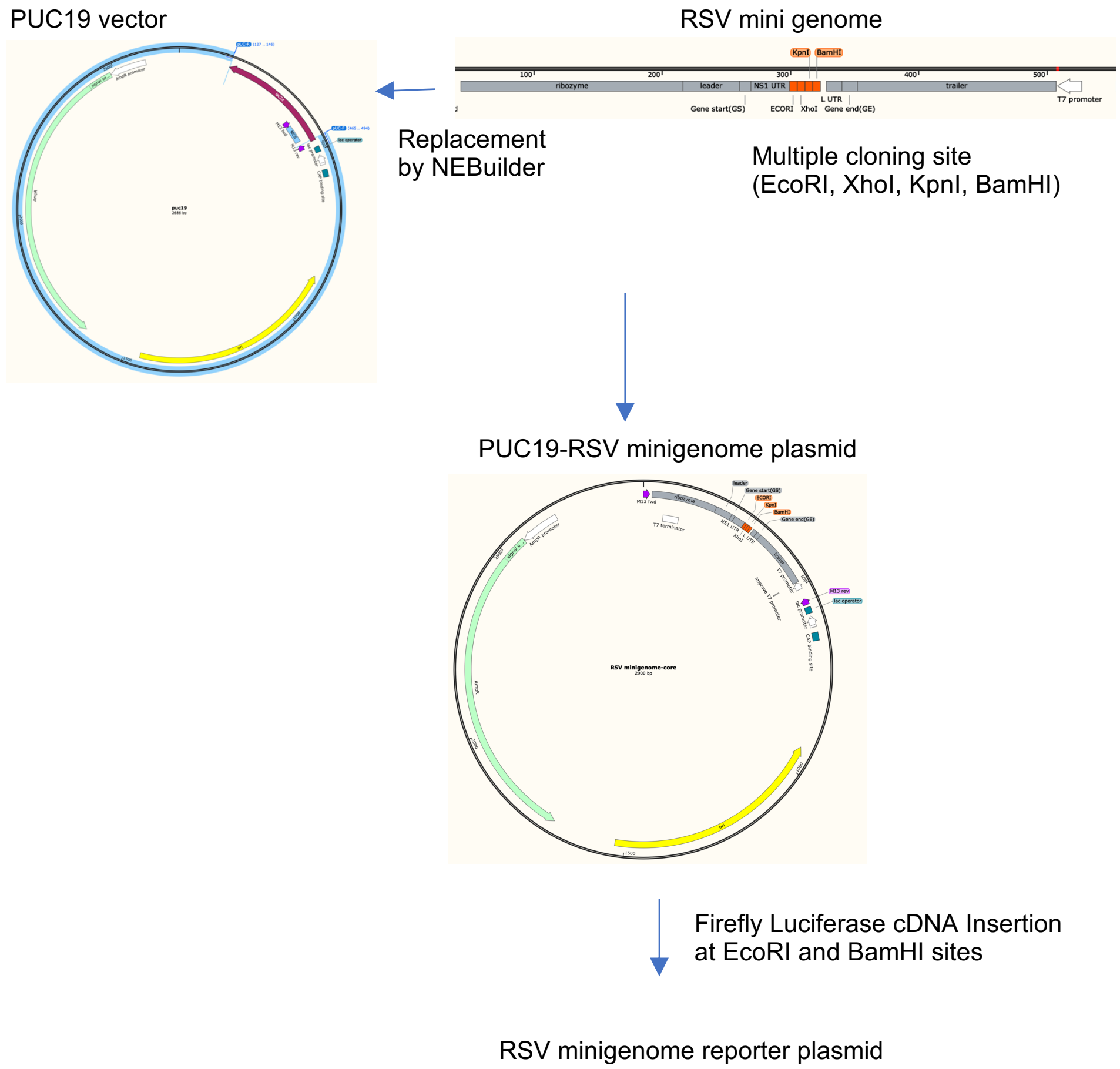

Supplementary Figure 15. Construction of plasmids used for the RSV minigenome reporter system. Schematic representation of the RSV minigenome reporter plasmid containing the luciferase reporter cassette and associated helper plasmids used in this study. Detailed cloning procedures are described in the Materials and Methods section.

Supplementary Figure 16

Uncropped images Fig. 3 B

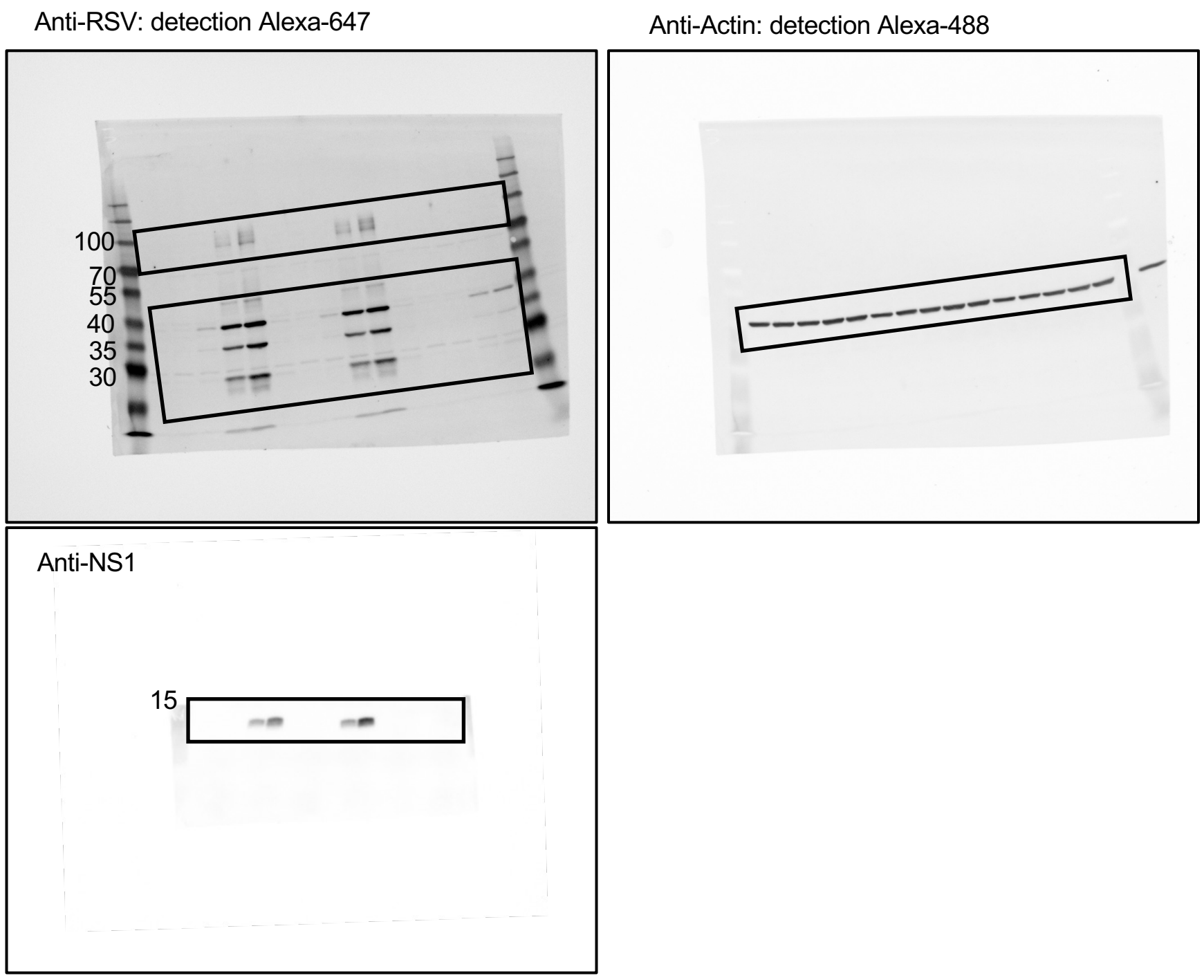

Supplementary Figure 17

Uncropped Fig. 3D

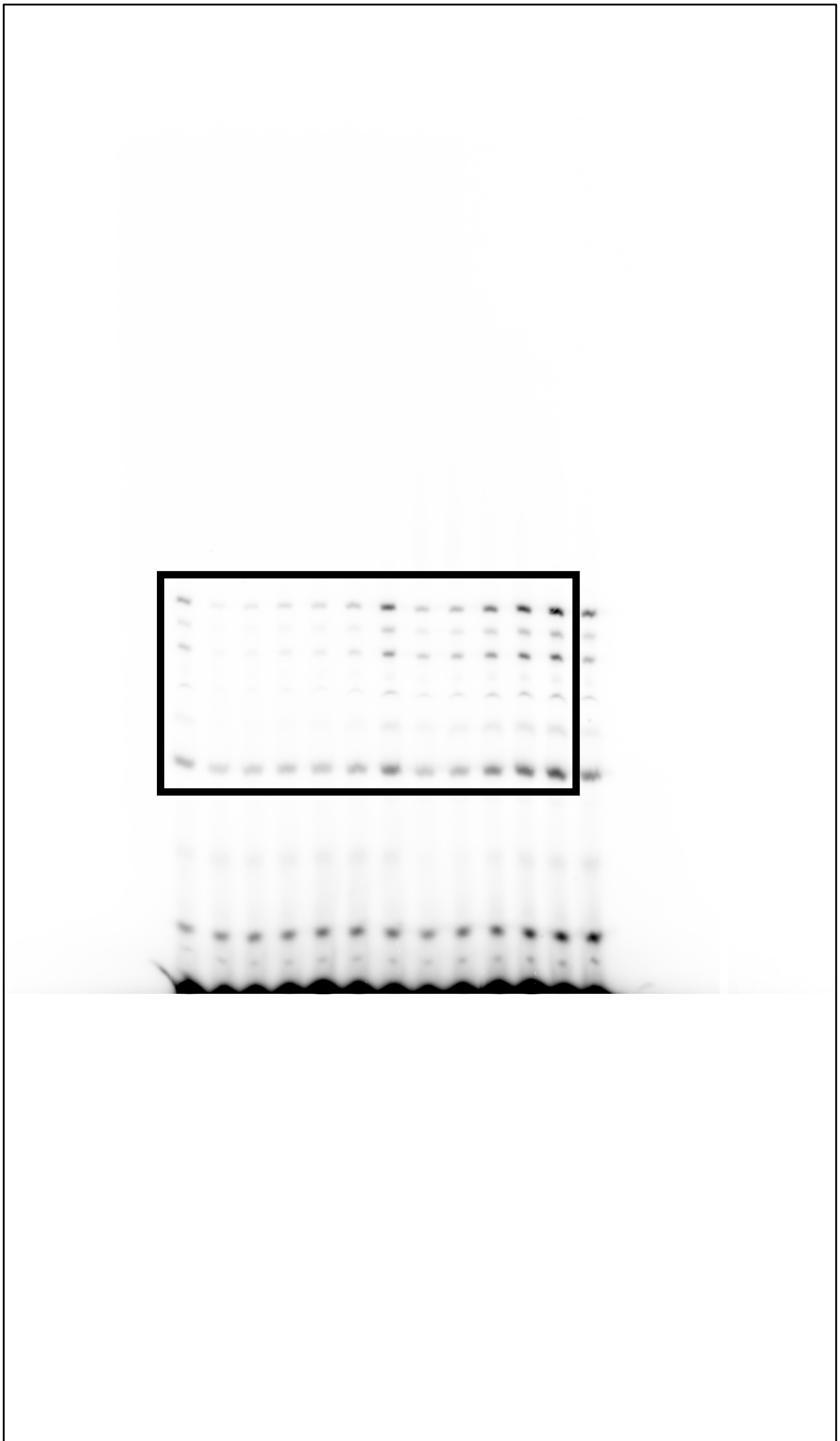

Supplementary Figure 18

Uncropped images Fig. 4 G

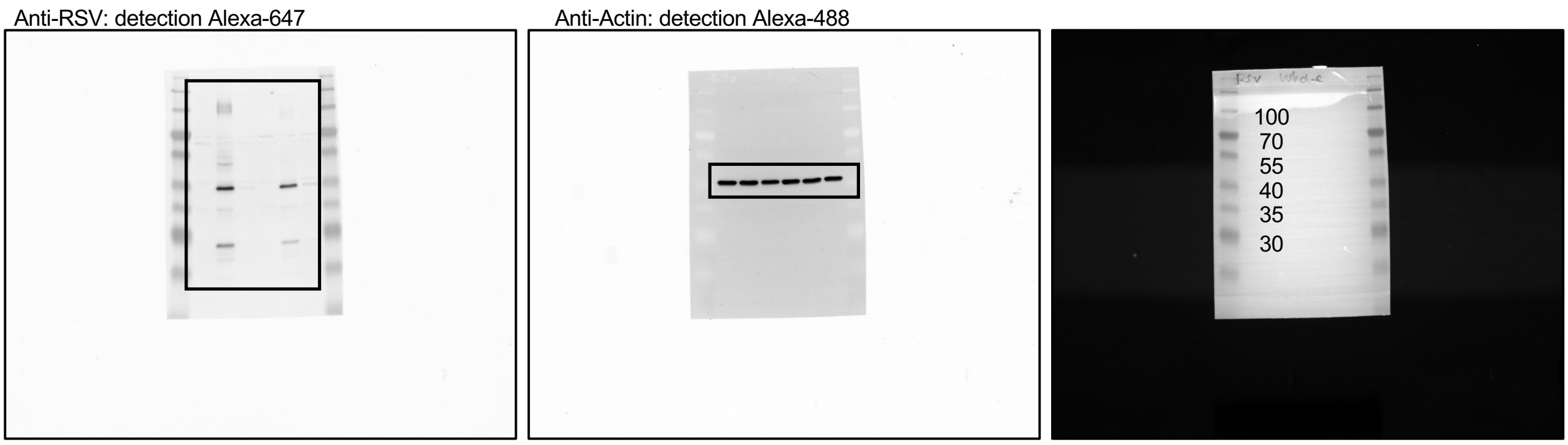

Supplementary Figure 19

Uncropped images Fig. 4K

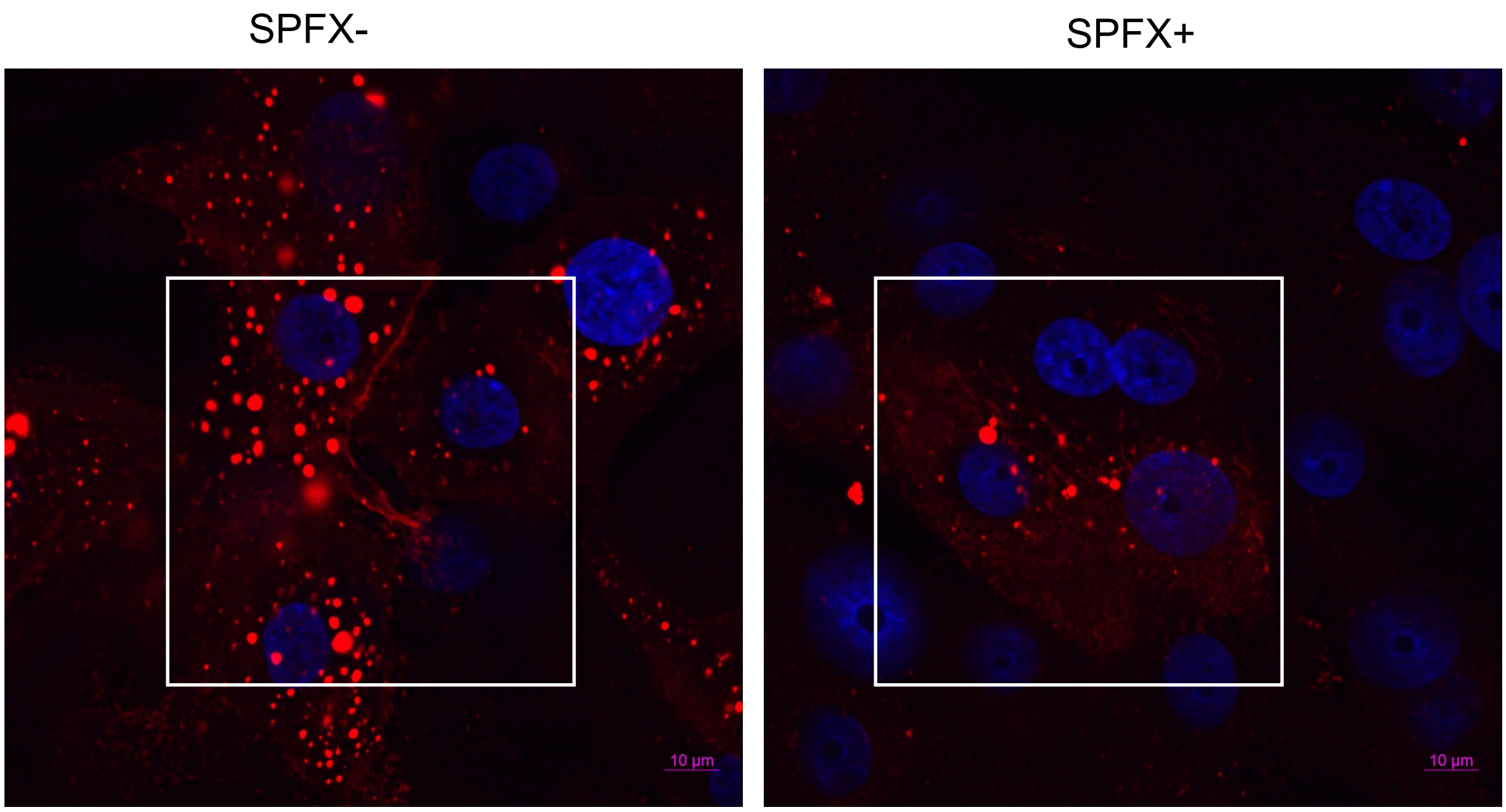

Supplementary Figure 20

Uncropped images Fig. 5A

Anti-HSP70

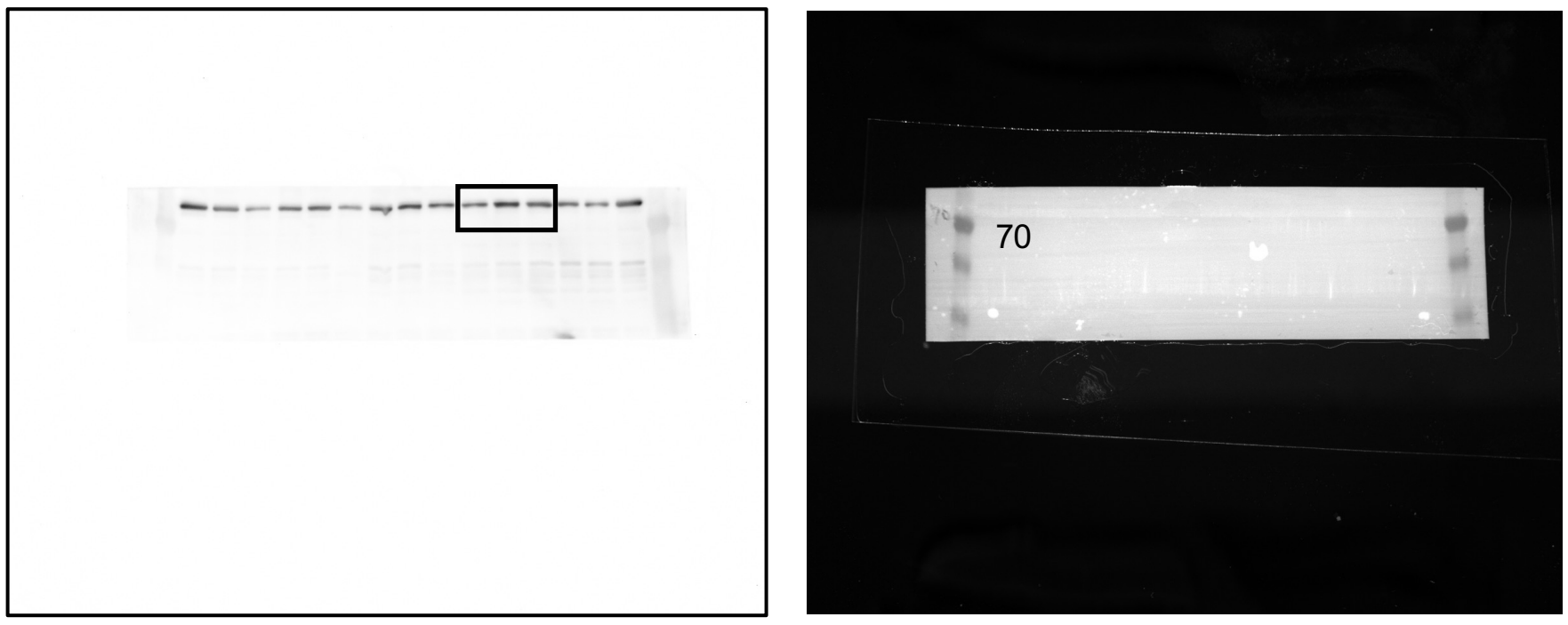

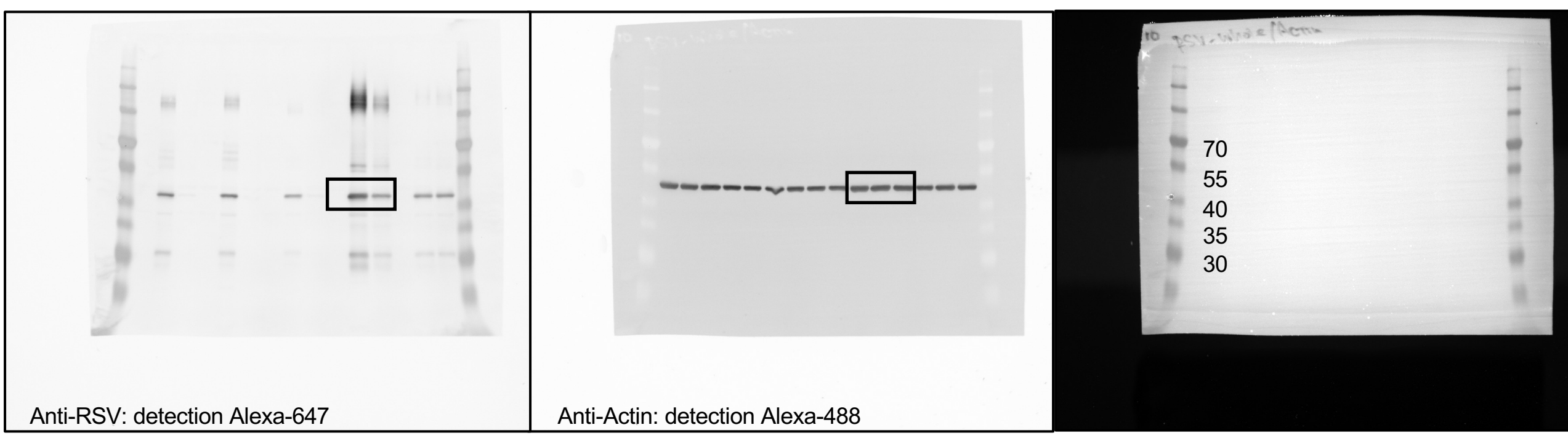

Supplementary Figure 21

Uncropped images Fig. 5B

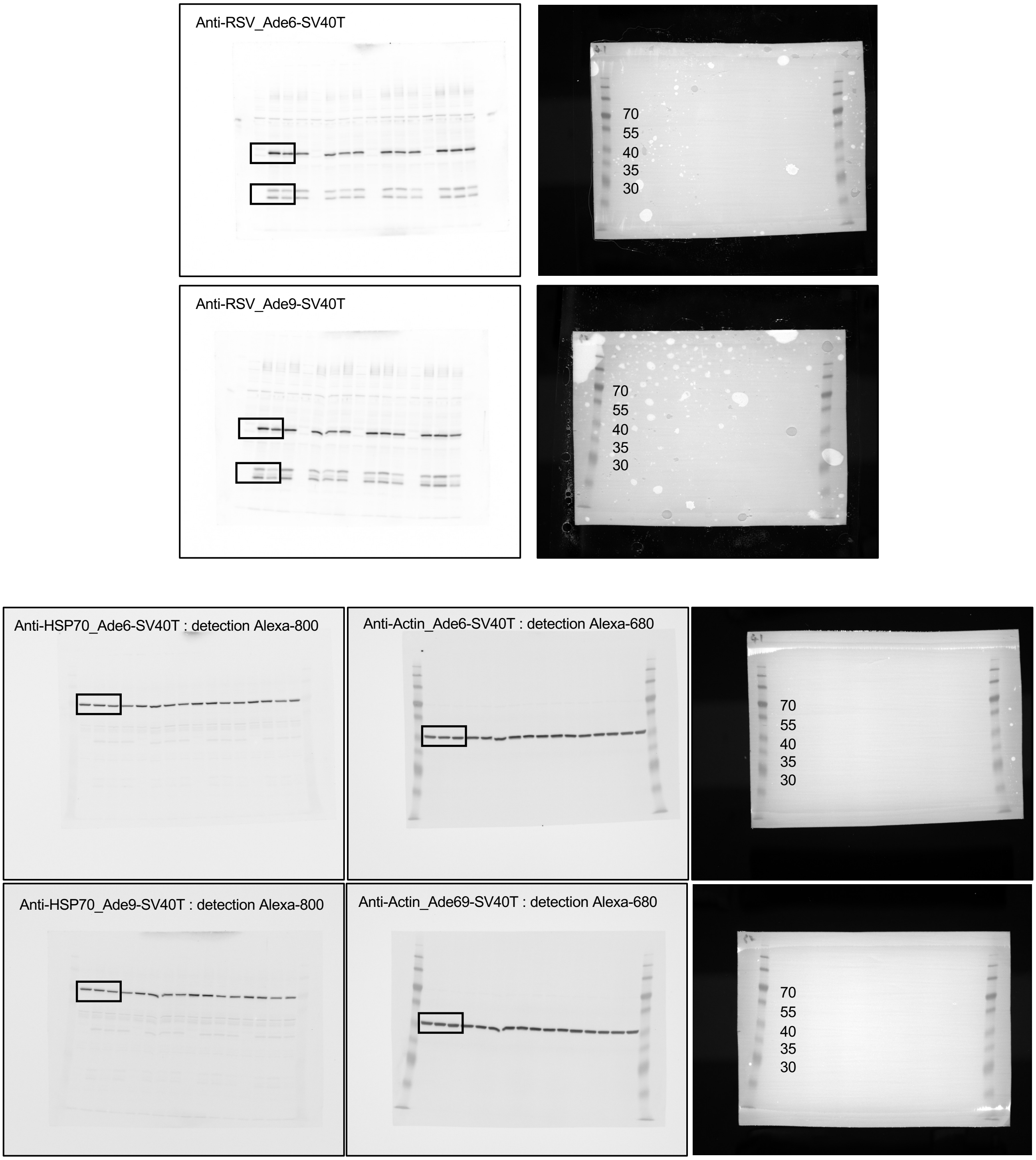

Supplementary Figure 22

Uncropped images Fig. 5D

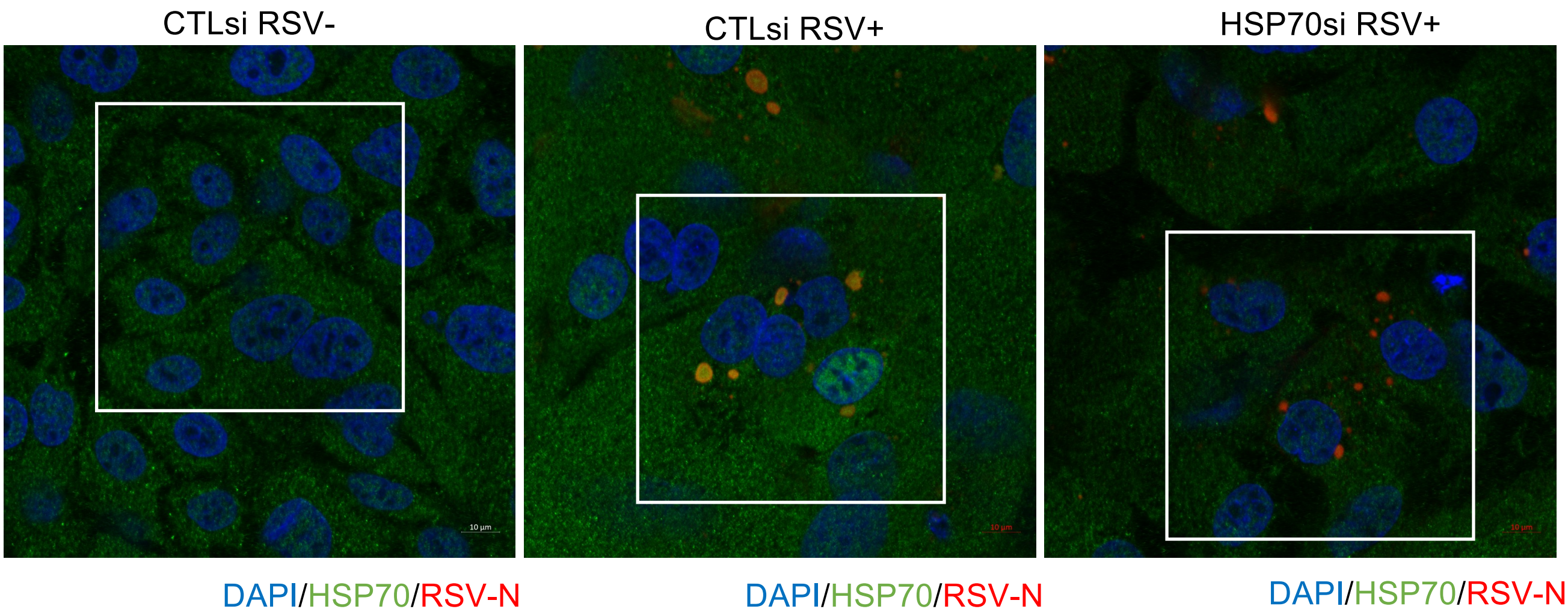

Supplementary Figure 23

Uncropped images S. Fig. 3 no1

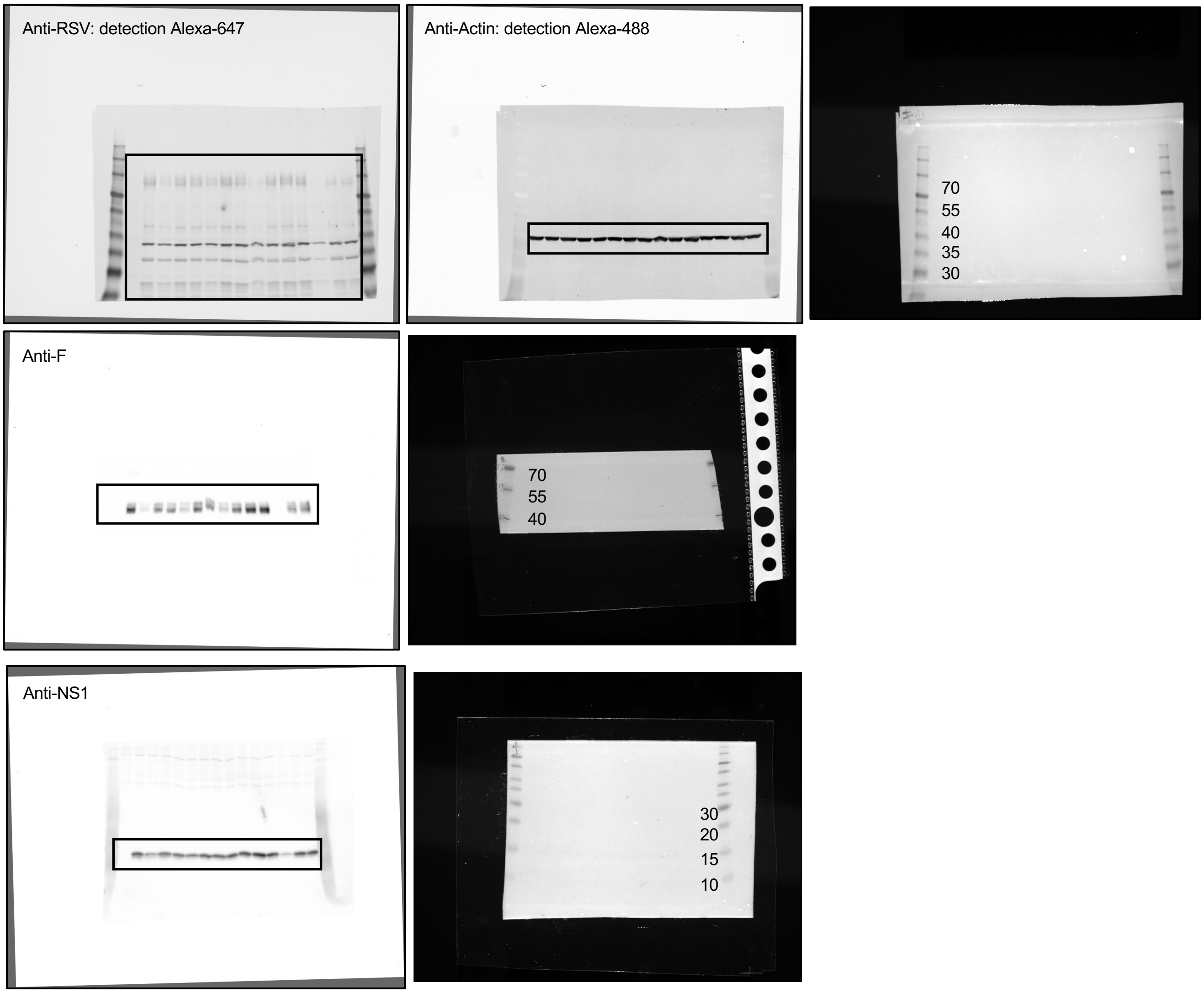

Supplementary Figure 24

Uncropped images S. Fig. 3 no2

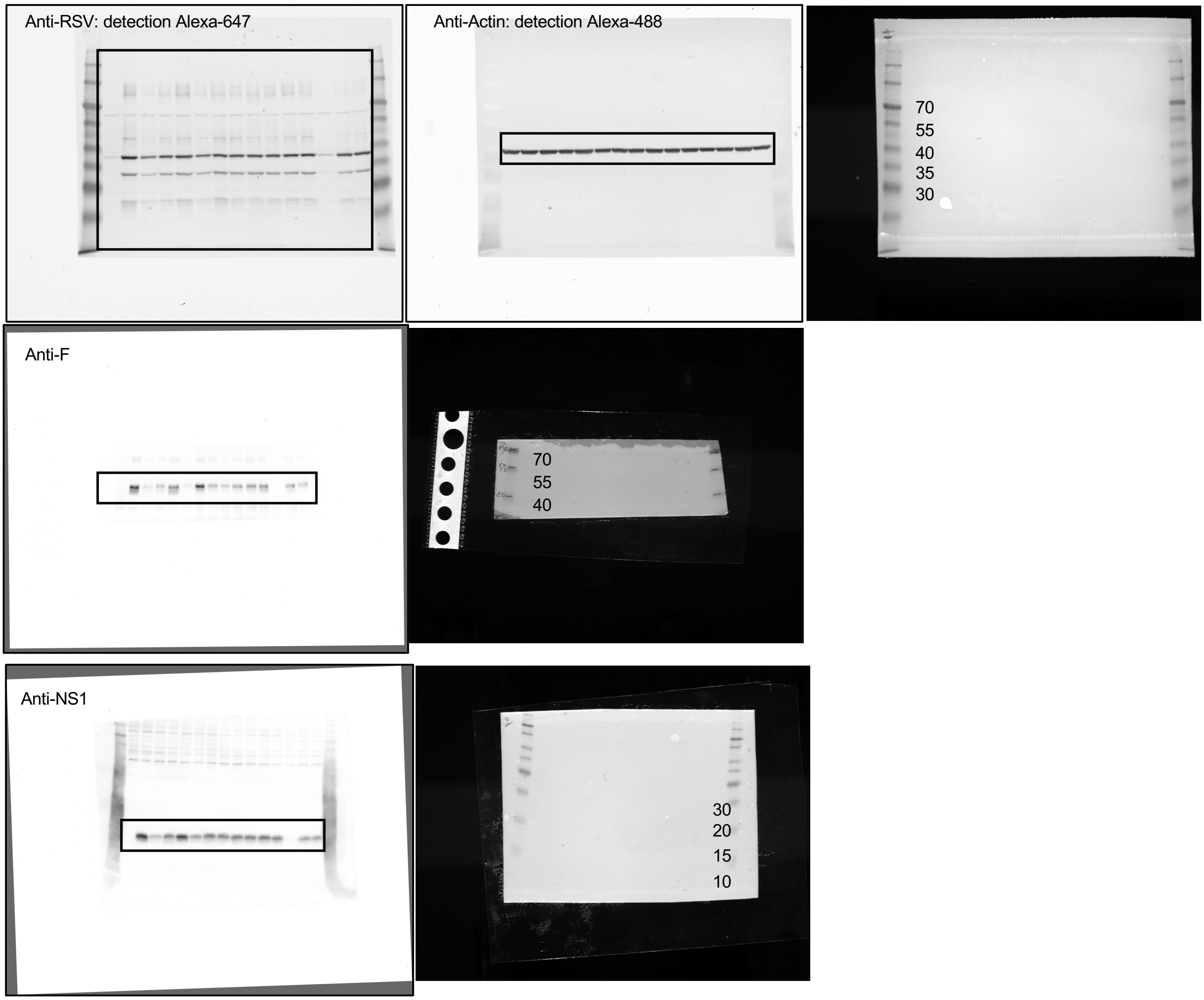

Supplementary Figure 25

Uncropped images S. Fig. 3 no3

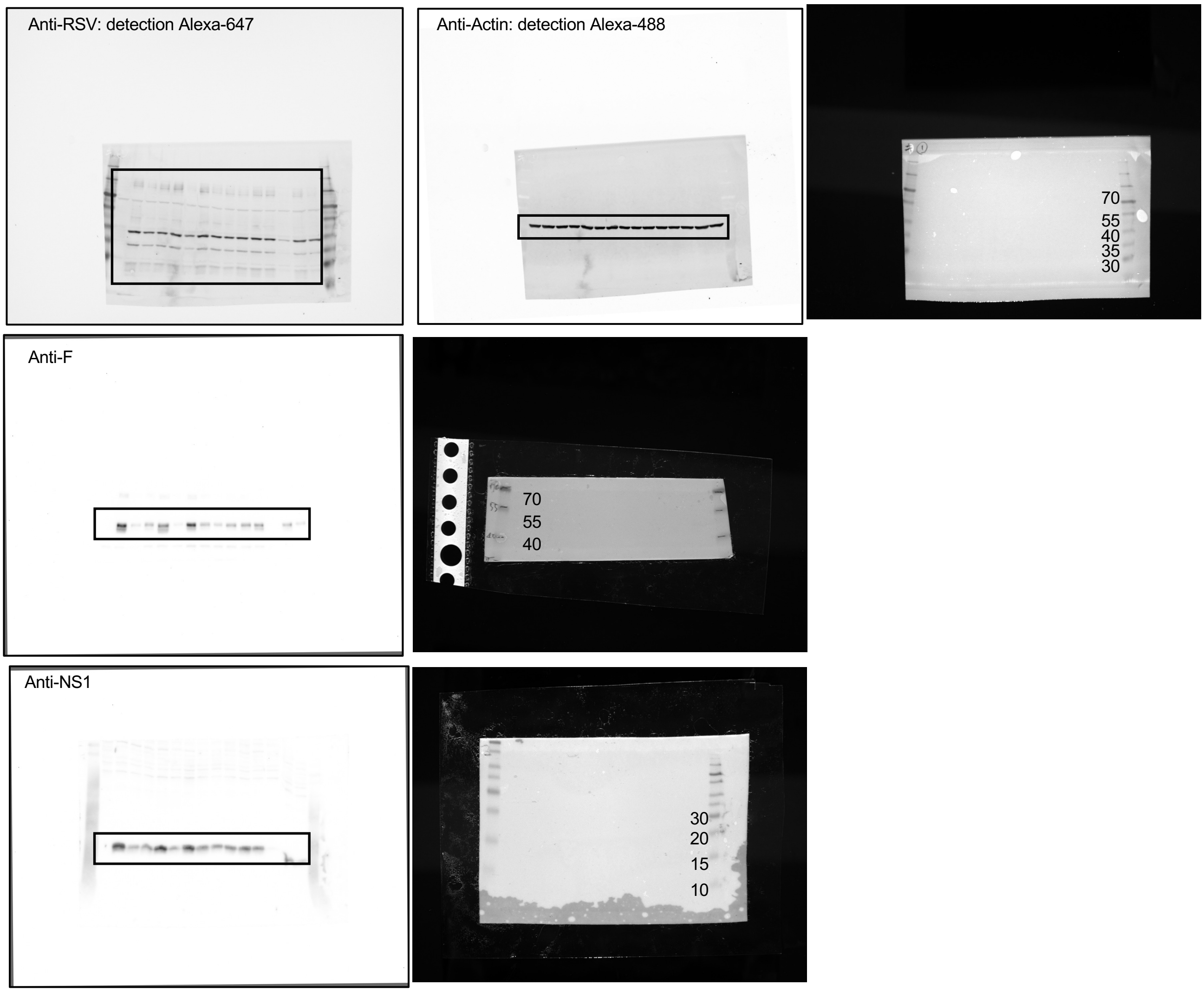

Supplementary Figure 26

Uncropped images S. Fig. 3 no4

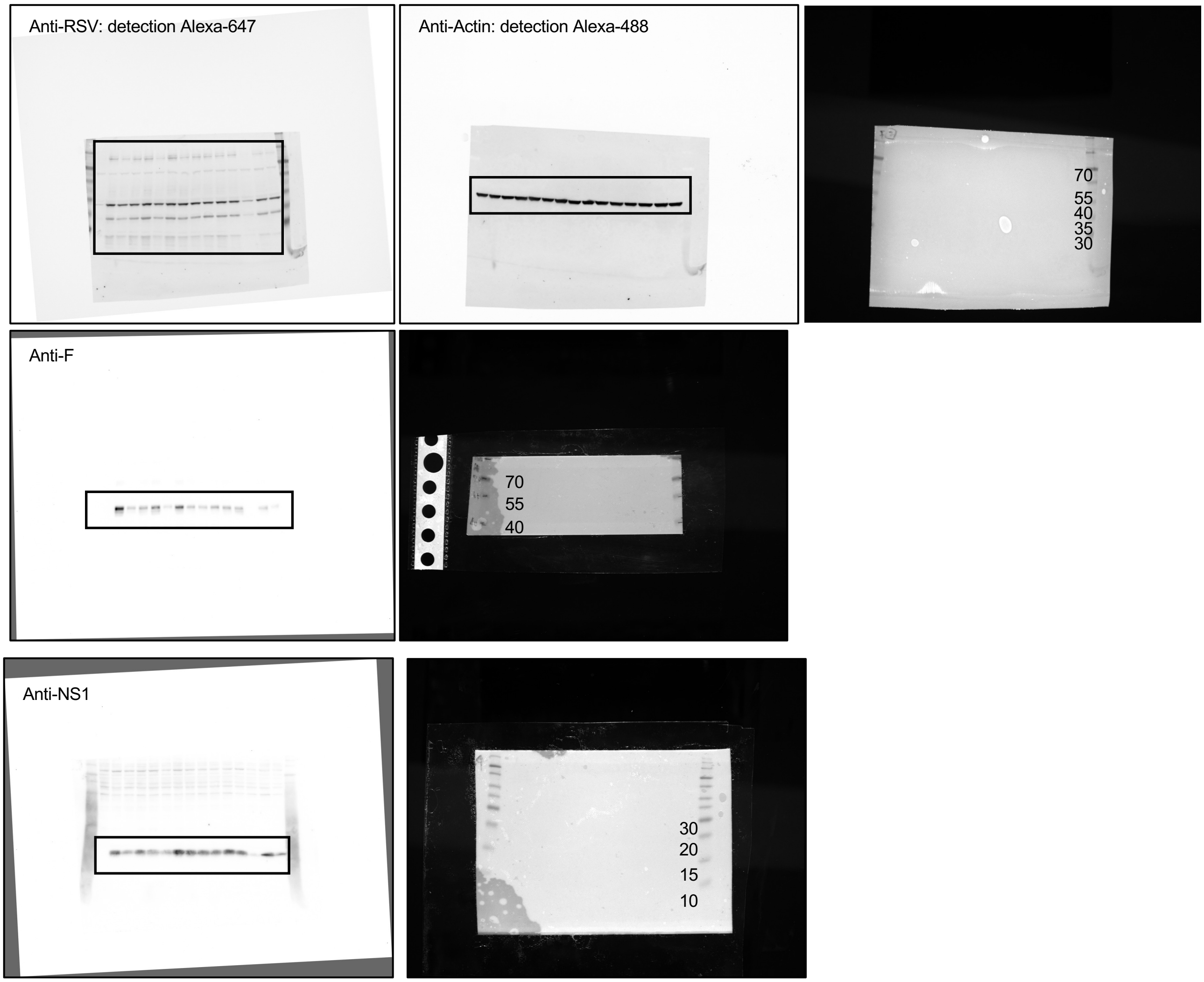

Supplementary Figure 27

Uncropped images S. Fig. 3 LCFX

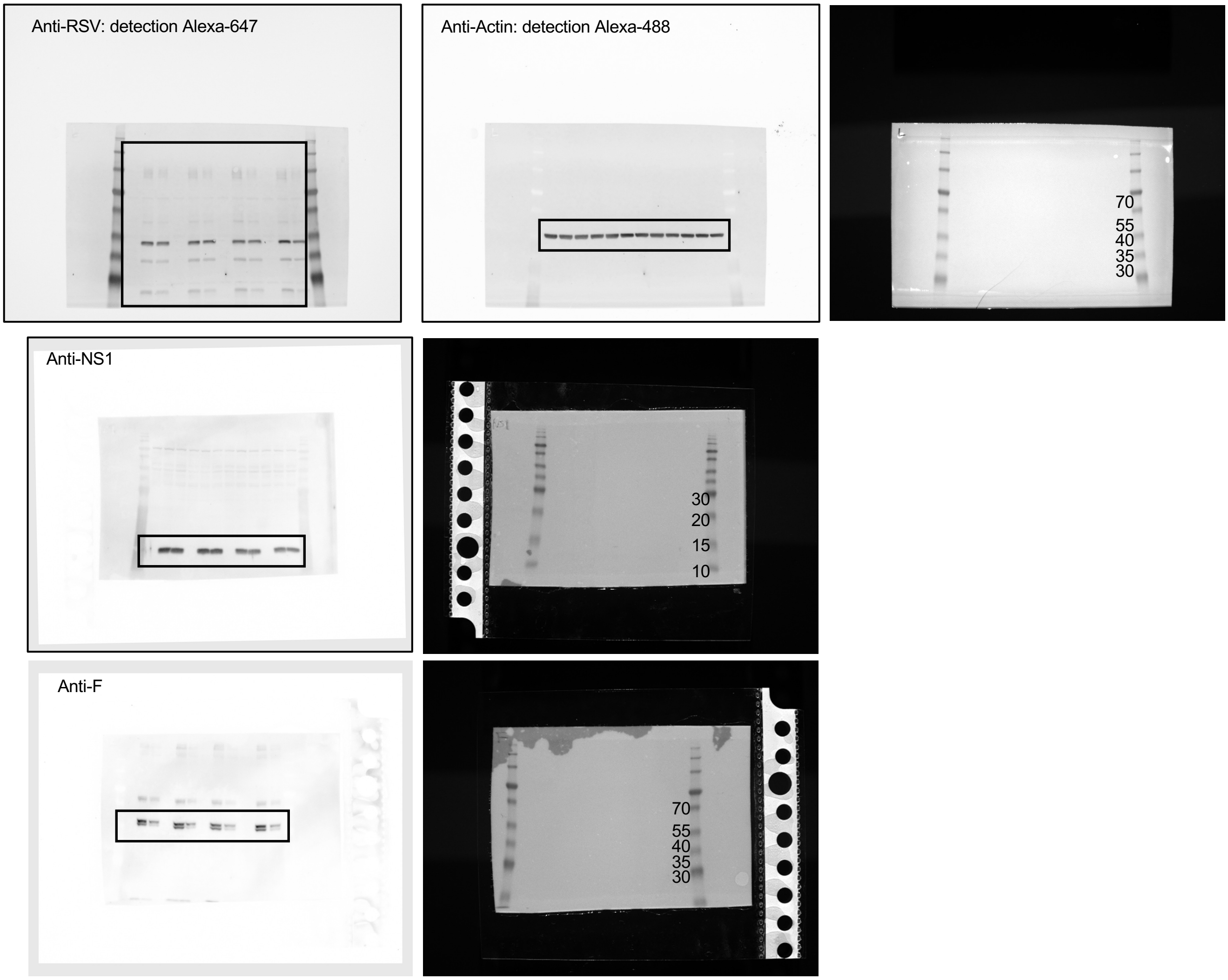

Supplementary Figure 28

Uncropped images S. Fig. 4C

Anti-Actin: detection Alexa-555

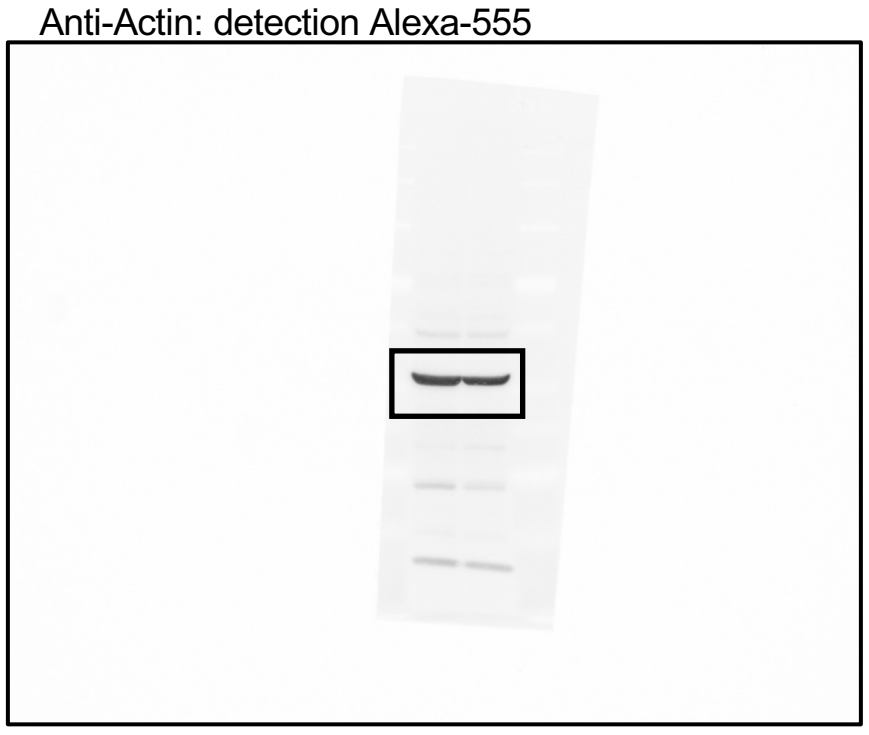

Anti-GFP: detection Alexa-647

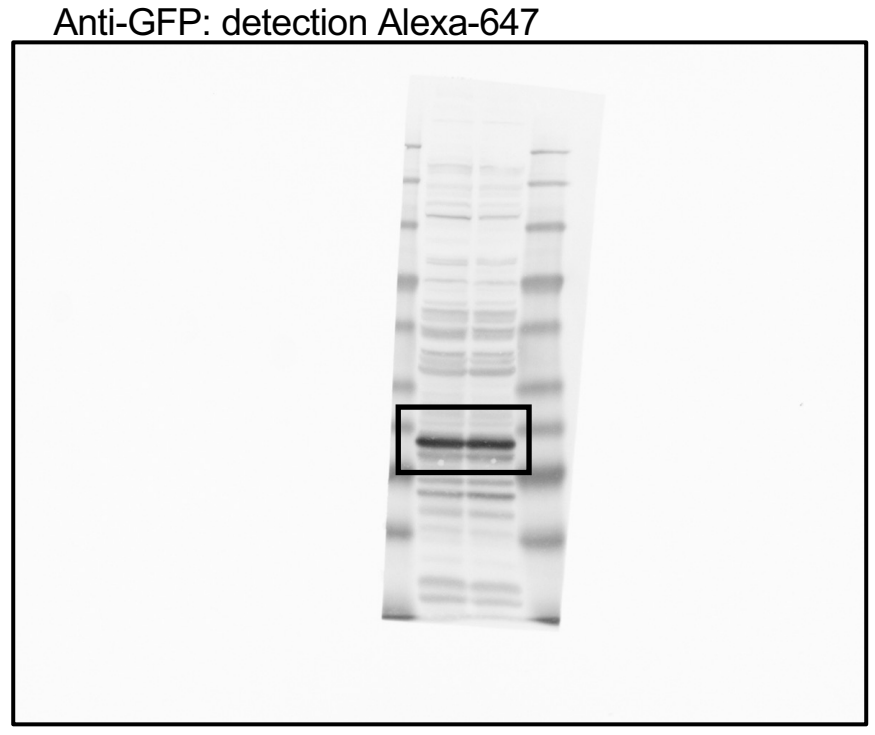

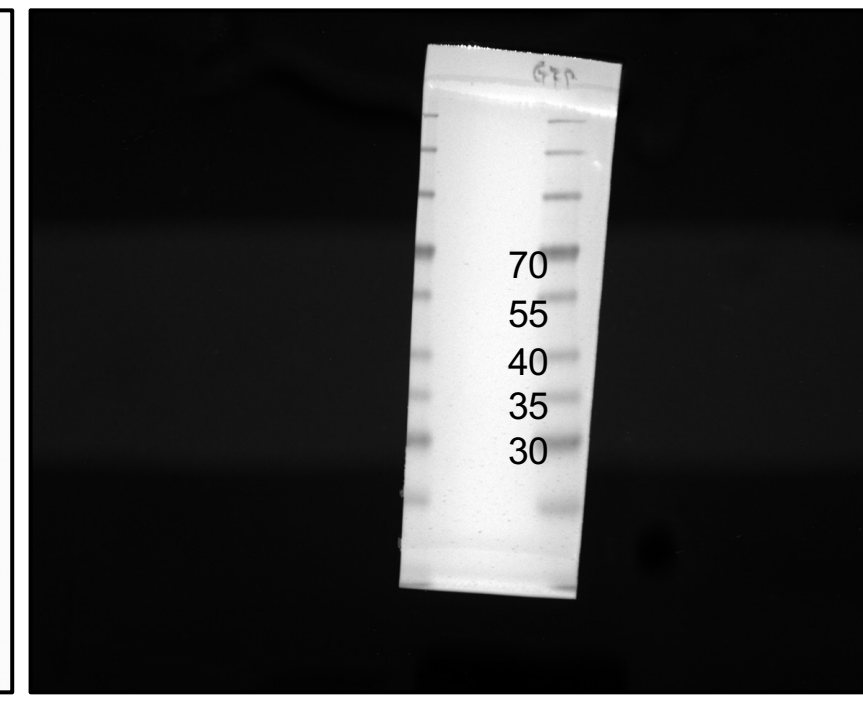

Supplementary Figure 29

Uncropped images S. Fig. 4E

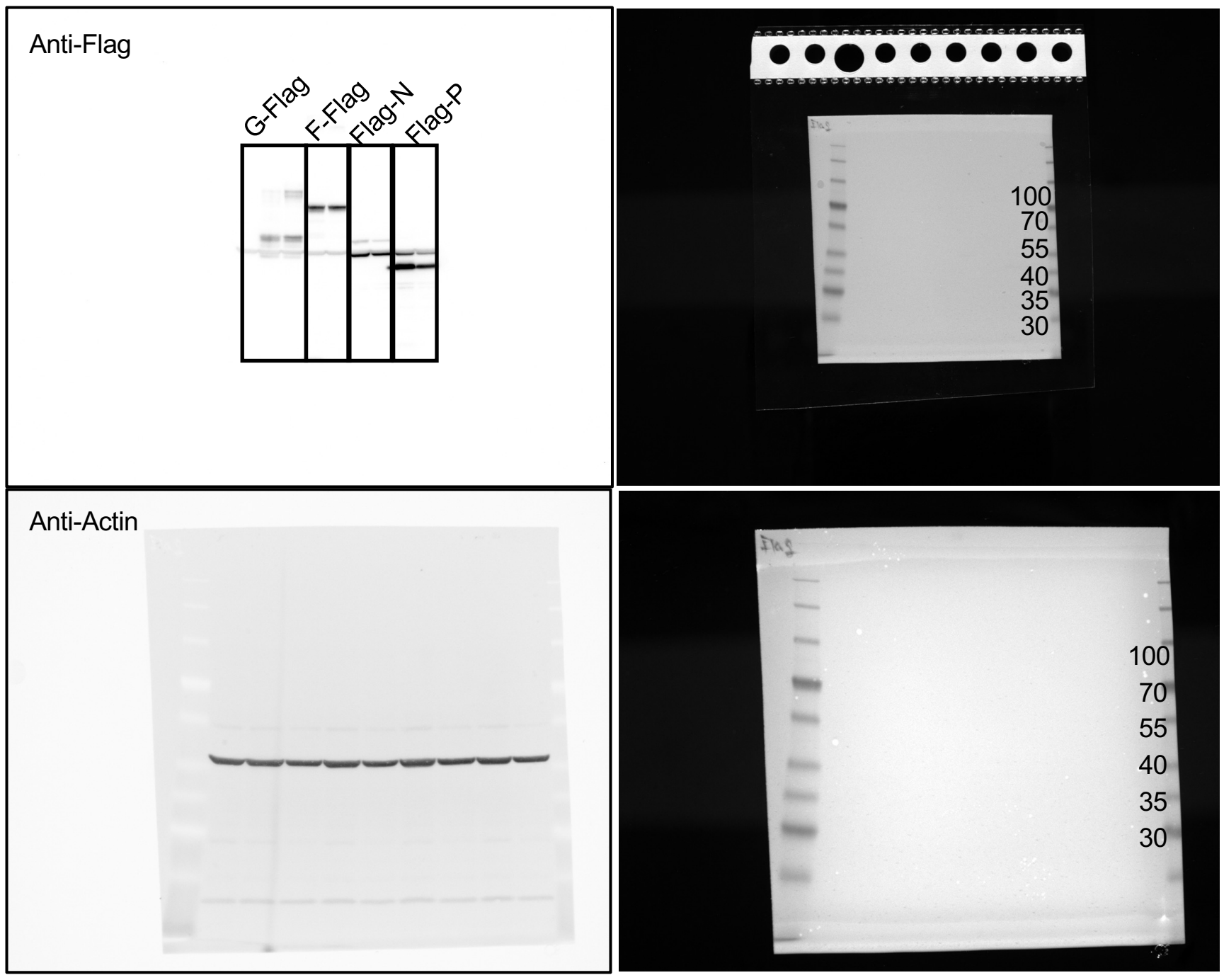

Supplementary Figure 30

Uncropped images S. Fig. 9A

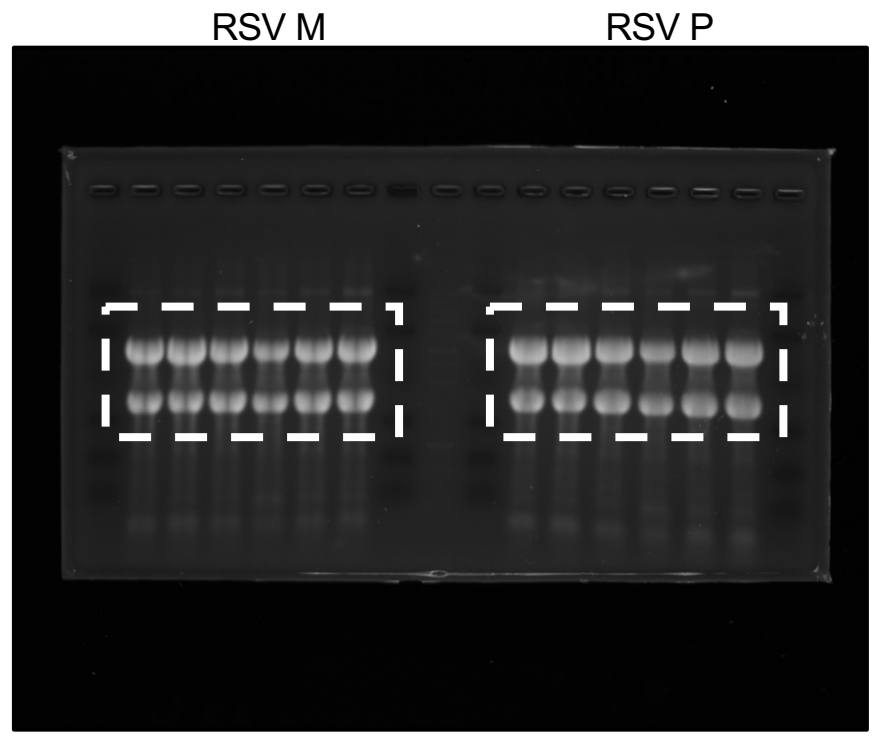

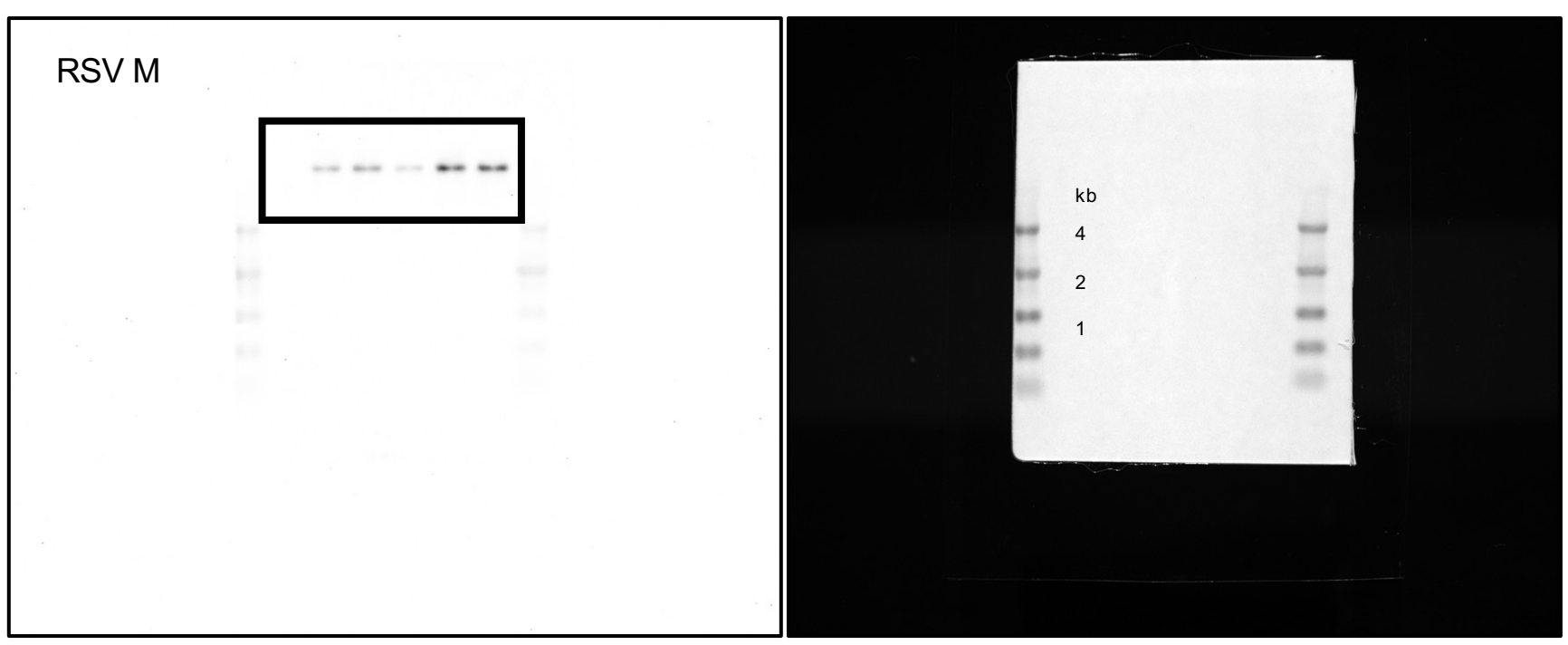

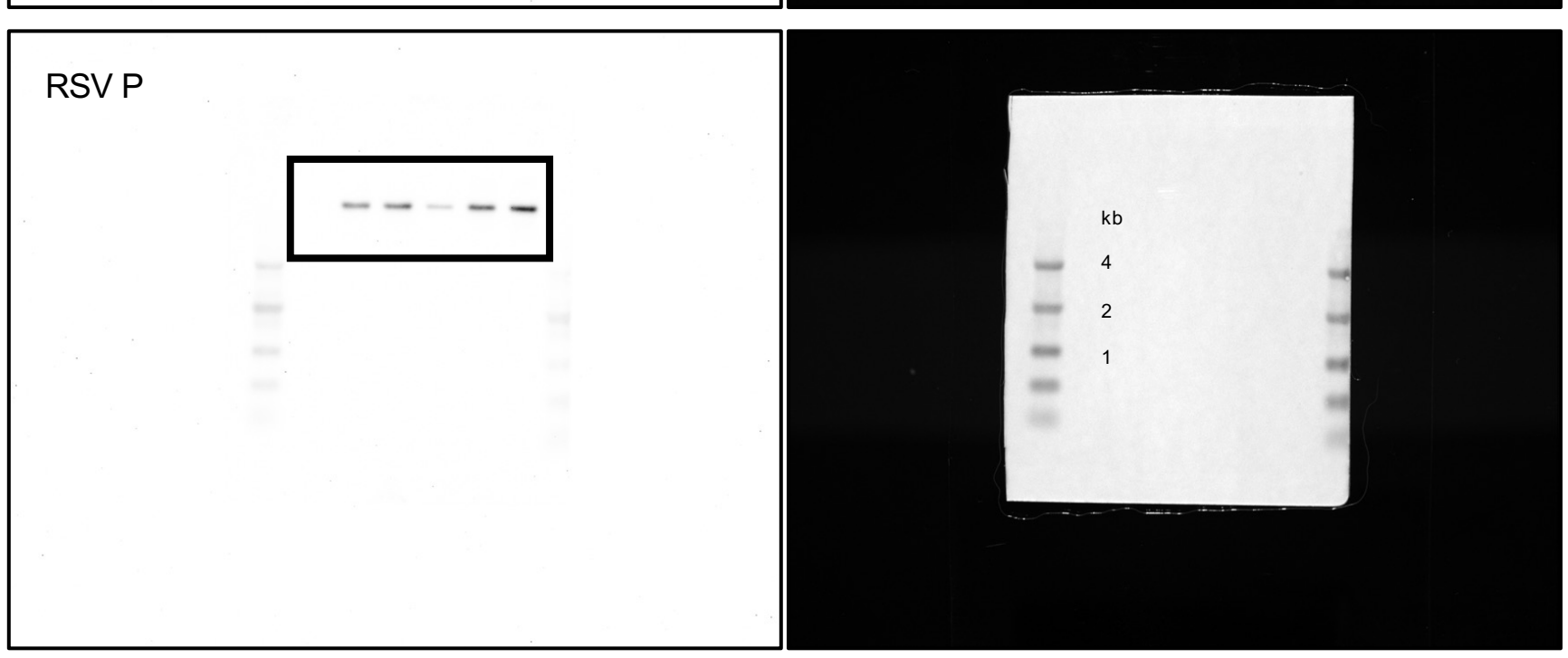

Supplementary Figure 31

Uncropped images S. Fig. 10A

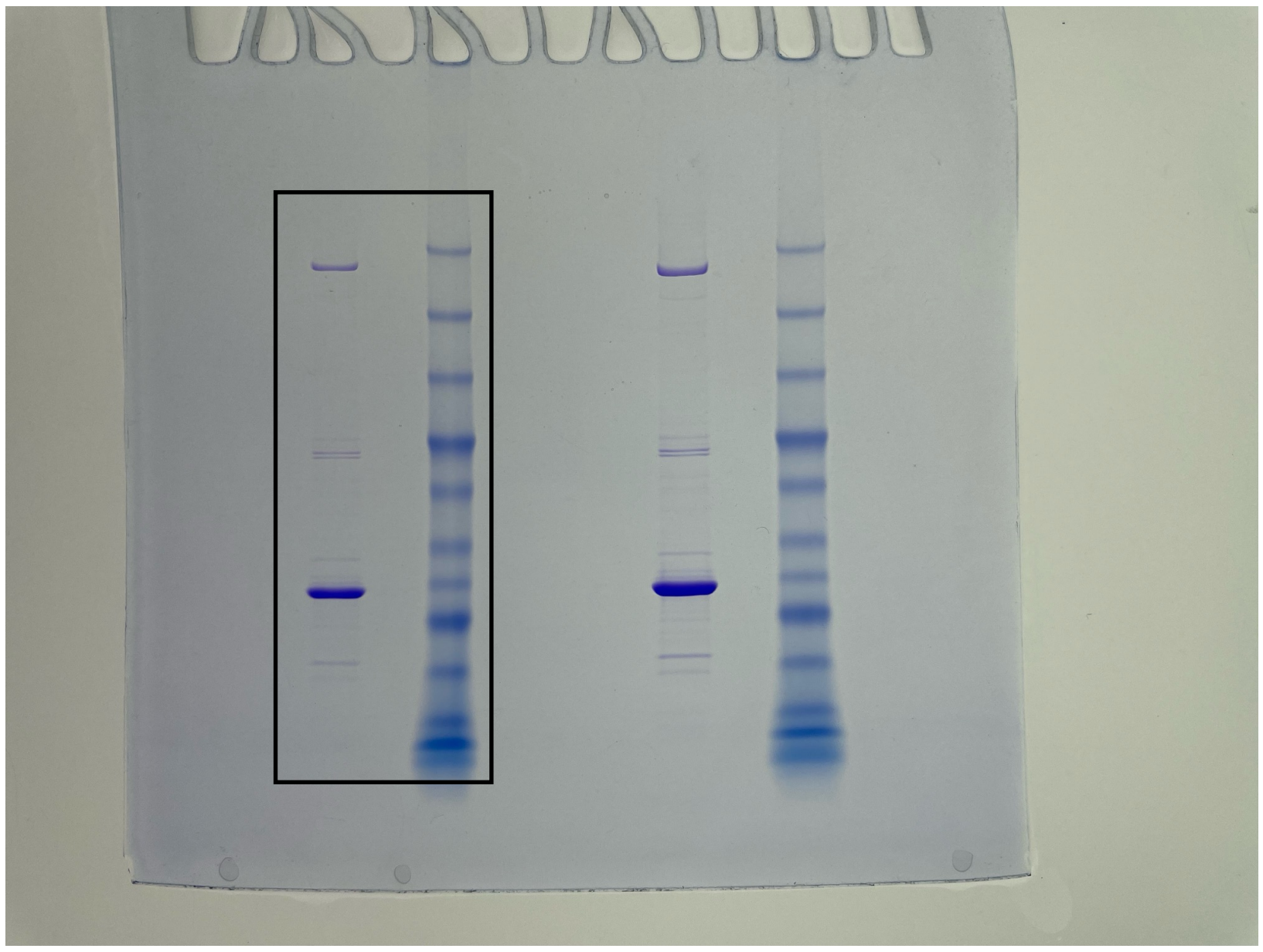

Supplementary Figure 32

Uncropped images S. Fig. 10B

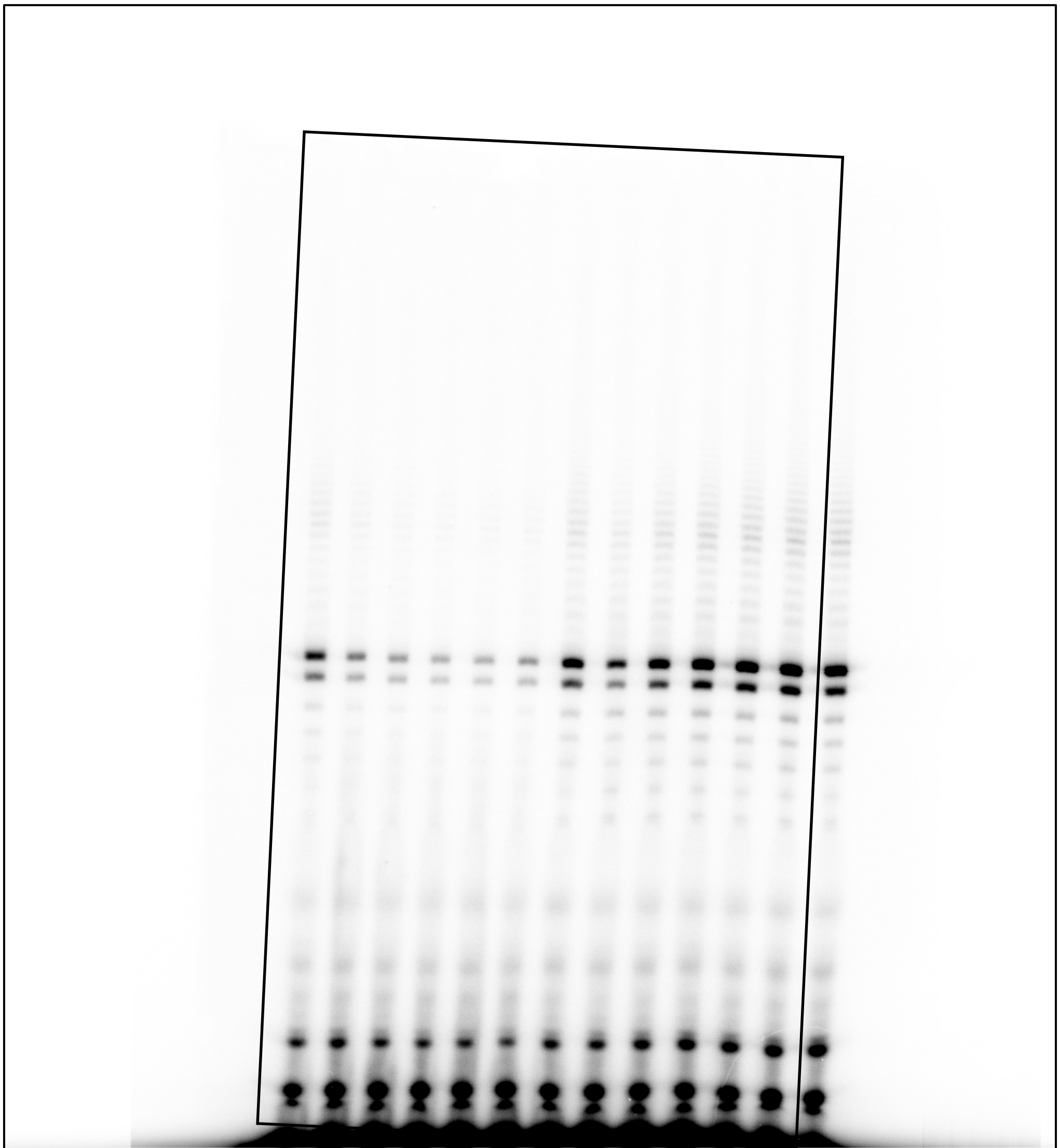

Supplementary Figure 33

Uncropped images S. Fig. 14C

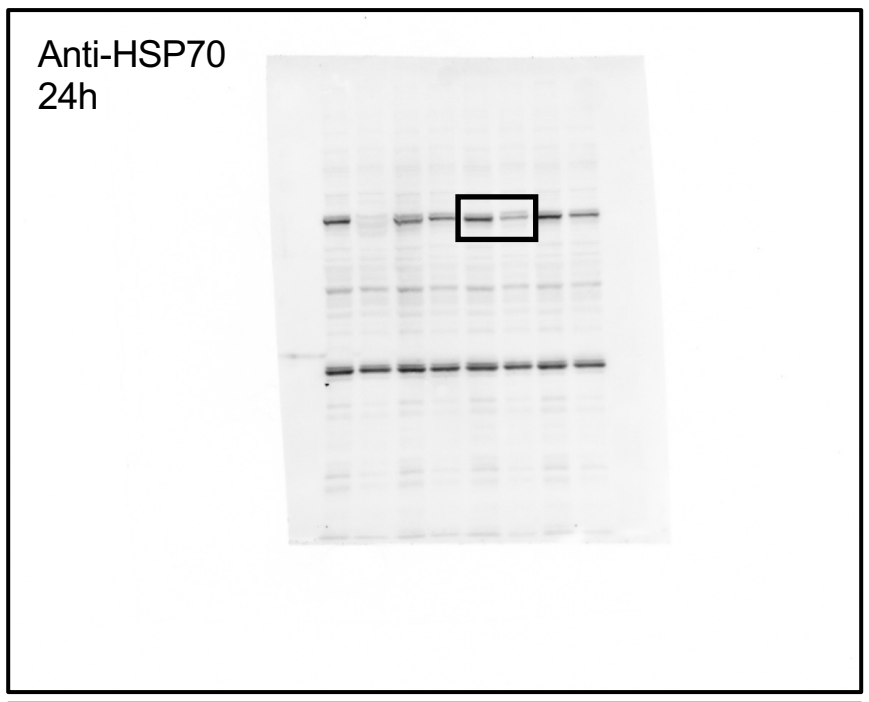

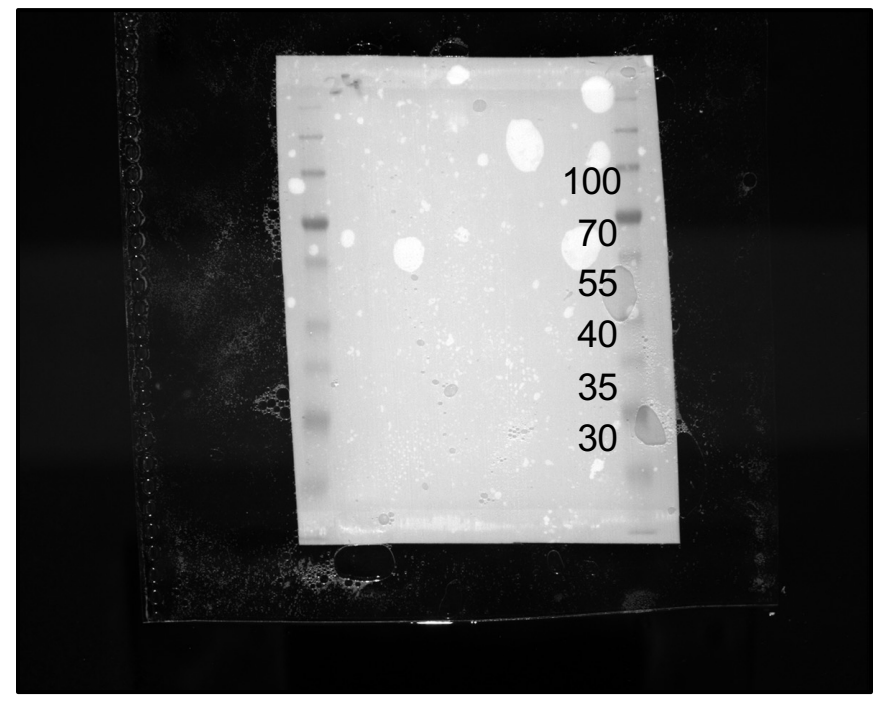

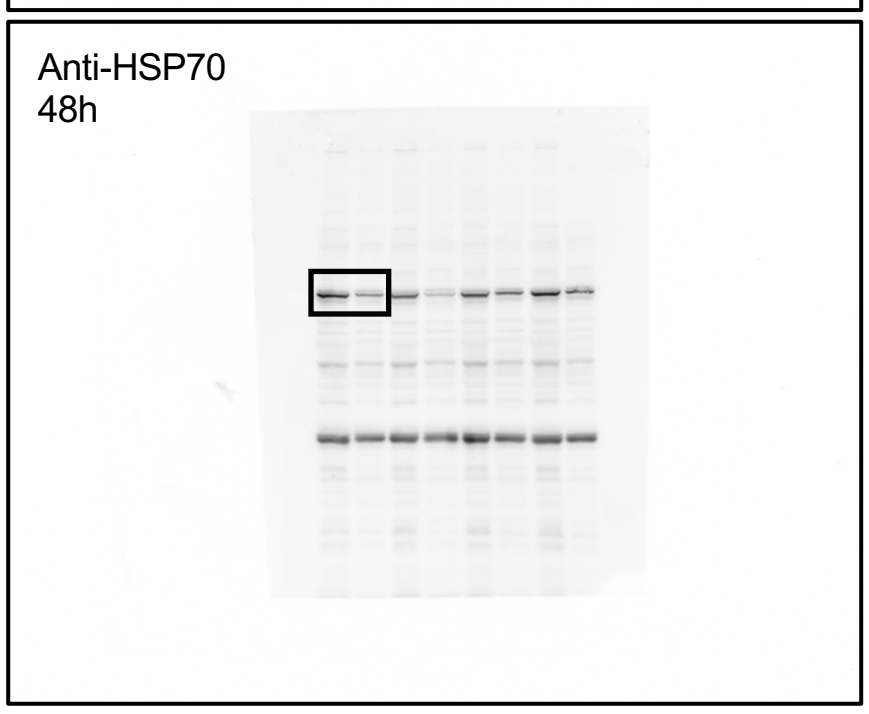

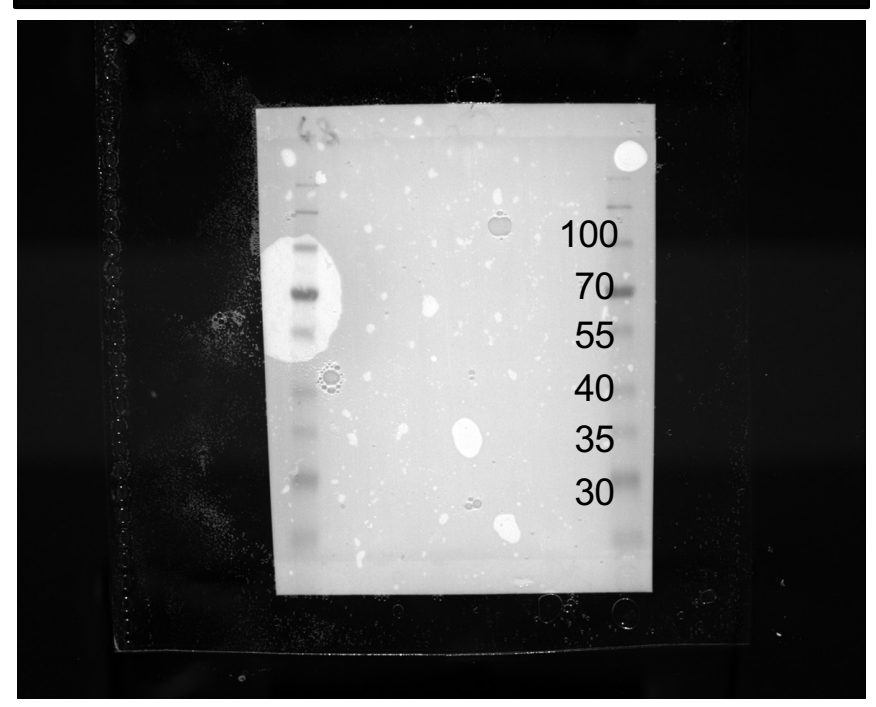

Anti-Actin: detection Alexa-680

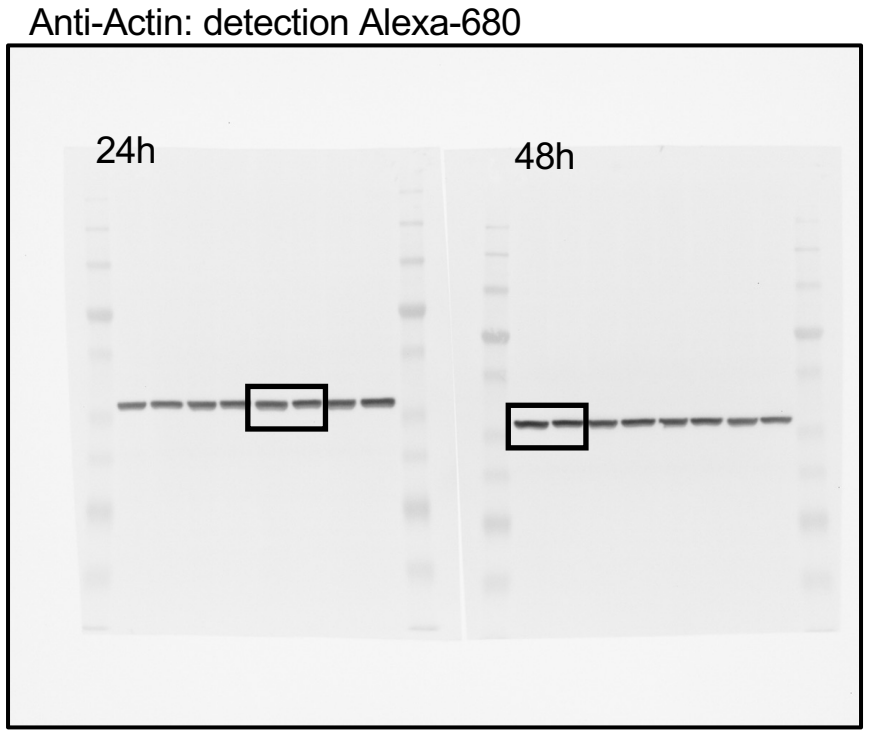

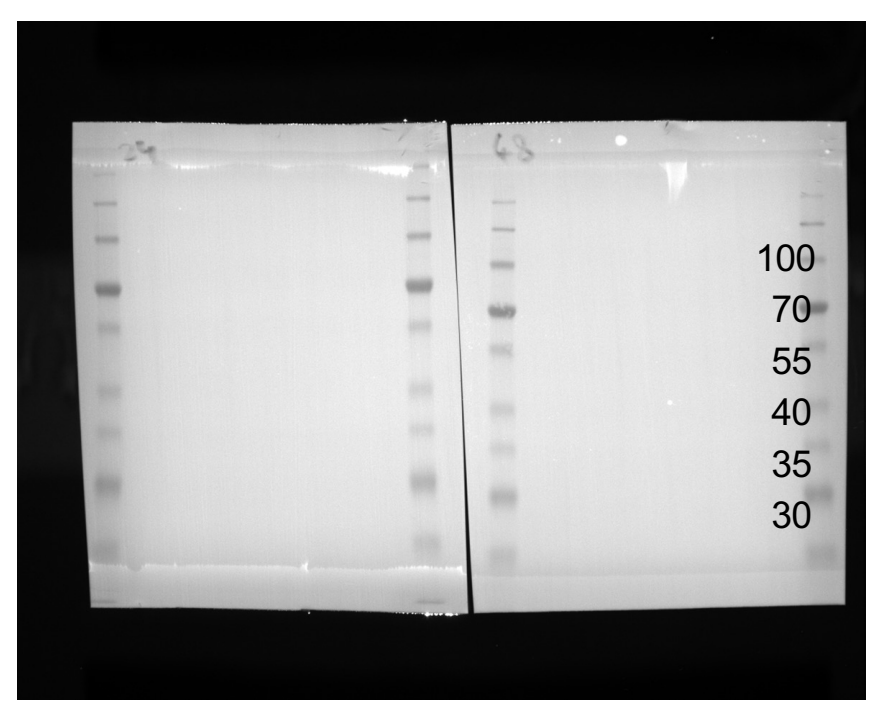

Supplementary Figure 34

Uncropped images S. Fig. 14D

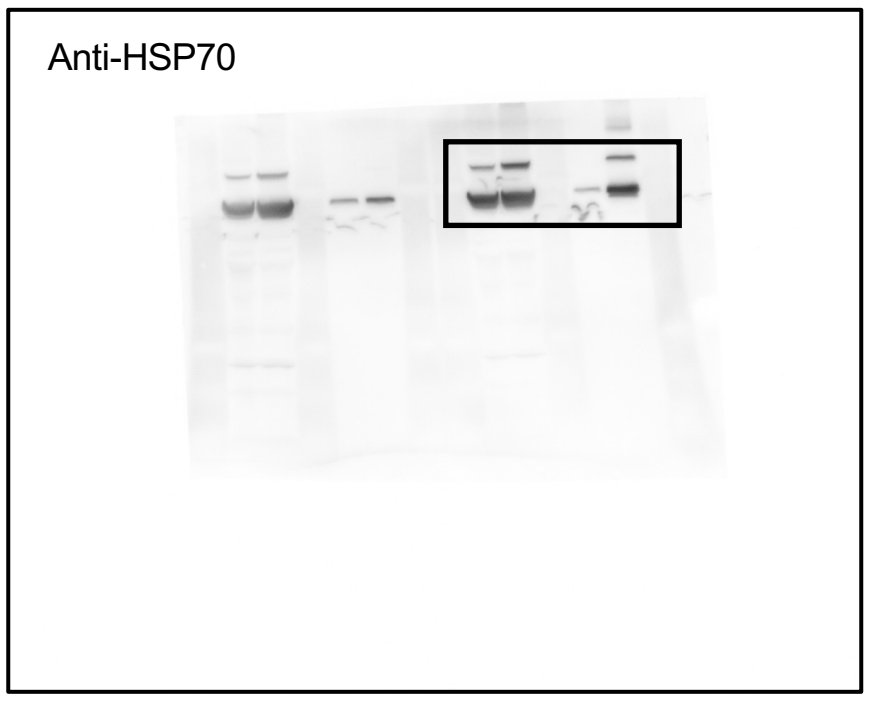

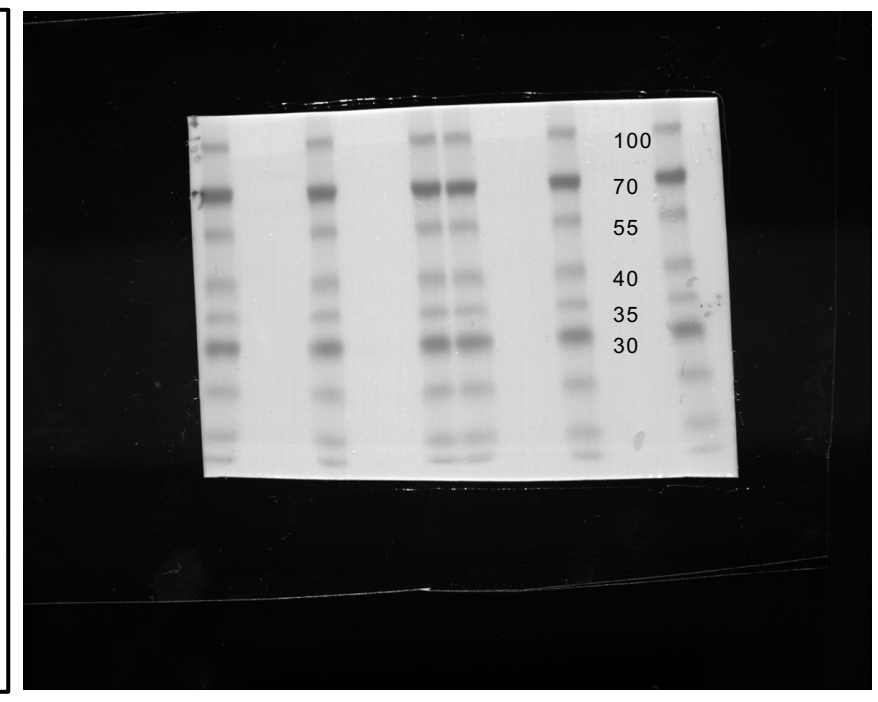

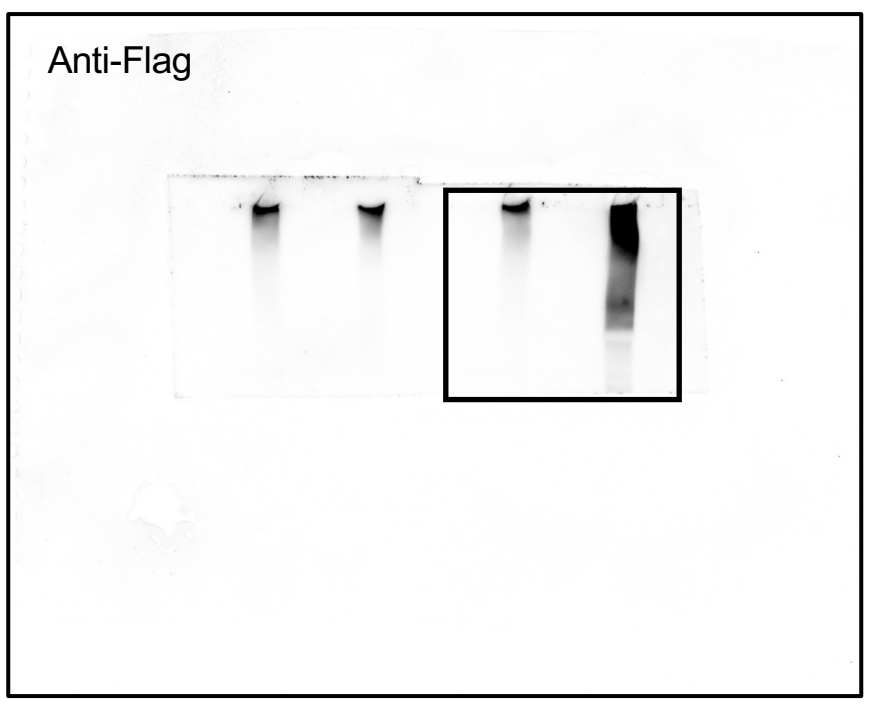

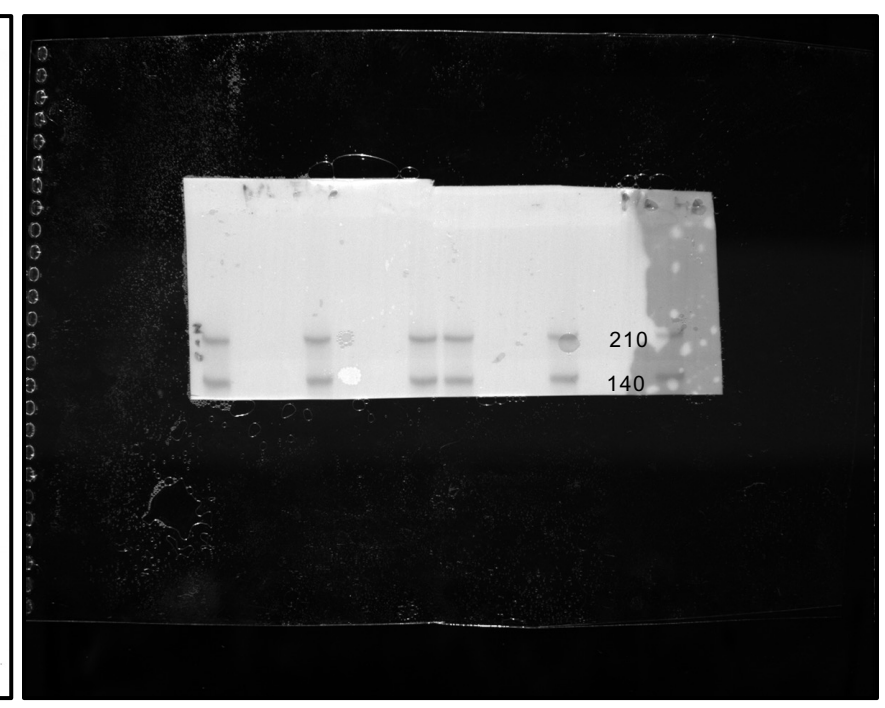

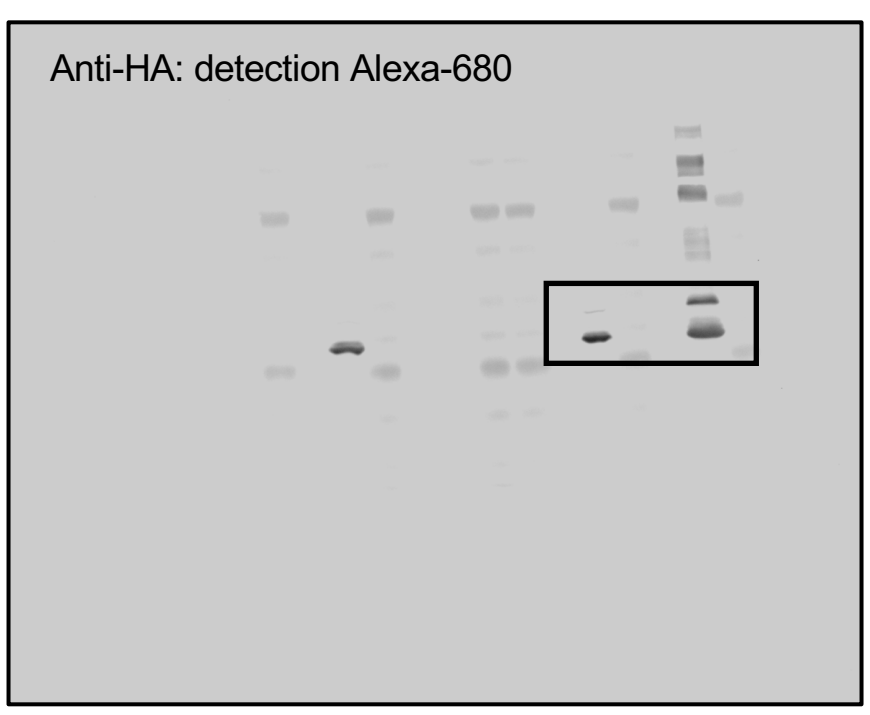

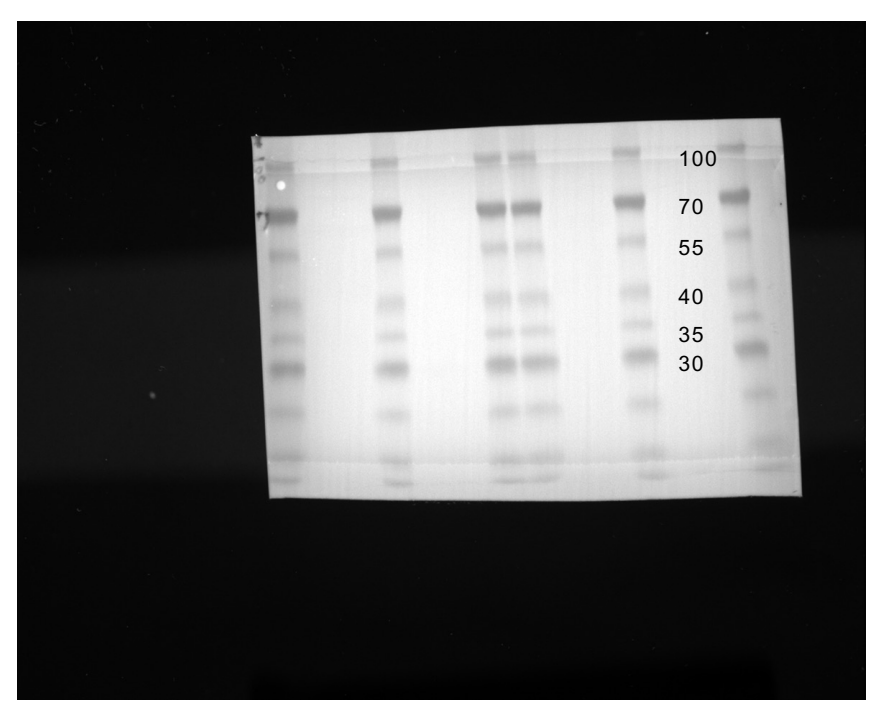

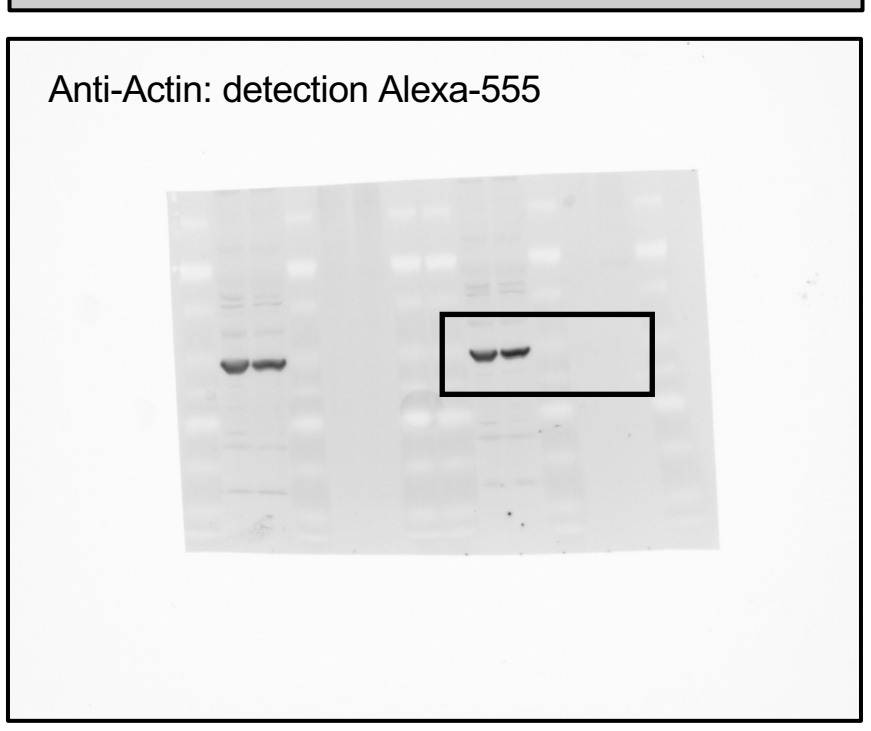

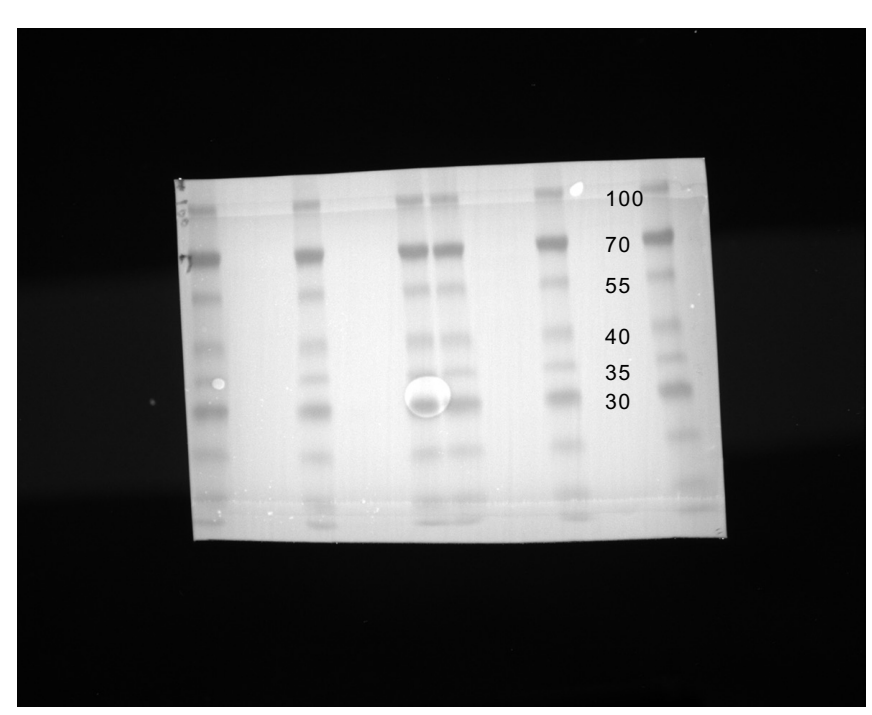

2. Supplementary Tables

Table S1. Antiviral potency (EC_{50}), cytotoxicity (CC_{50}), and selectivity index (SI) of screened compounds and fluoroquinolone derivatives against RSV

	CC_{50}	EC_{50}	Selectivity Index	Reference
Cyclopamin	171.2	1.99	86.0	
Ciprofloxacin	1185	24.99	47.4	
Promethazine	51.53	28.56	1.8	
Lansoprazol	114.6	24.17	4.7	
Montelukast	86.9	25.53	3.4	
Sparfloxacin	373.3	14.99	24.9	
Orbifloxacin	625.1	80.52	7.8	
Amoxicillin	n.d*	N.D**	-	
albuterol	n.d	N.D	-	
clavulanate	n.d	N.D	-	
Cephalexin	n.d	N.D	-	
Nystatin	n.d	N.D	-	
Trimethoprim	n.d	N.D	-	
Sulfamethoxazole	n.d	N.D	-	
Acetaminophen	n.d	N.D	-	
Chlorpheniramine	n.d	N.D	-	
Ibuprofen	n.d	N.D	-	
Cefdinir	n.d	N.D	-	
Antipyrine	n.d	N.D	-	
Ranitidine	n.d	N.D	-	
Ribavirin	200	6.9	29	Ref18, Liuzzi et al., 2005
RSV L-IN-5	10.7	0.1	107	Ref18, Liuzzi et al., 2005

* n.d: not determined
** N.D: Not Detected

2. Supplementary Tables

Table S2. Primers used for PCR of P and M gene fragments.

Primer names	*Sequences (5'-3')*
RSV-M-forward	*TAATACGACTCACTATAGGGTAGAAAAAGACGATGACCCTGCATCAC*
RSV-M-reverse	*GCAAATCGTGTAGCTGTGTGCTTCC*
RSV-P-forward	*TAATACGACTCACTATAGGGTCCATGGAGAAGATGCAAACAACAGG*
RSV-P-reverse	*TCACTATCATTCCCTTCCAATAGGTTGTTC*

The underlined sequence is a T7 promoter for RNA transcription.

3. Supplementary Methods

In vitro RNA synthesis assays (de novo synthesis)

The reactions contained 1 mg RSV-L/P, 0.2 μM RNA template (3’-UGCUCUUUUUUCA-5’), were incubated 30 °C for 10 min in reaction buffer, and then 500 μM ATP, 500 μM UTP, 10 μM GTP, and 100 nM of [α-32P]-GTP, and AVG-233 or SPFX were added and incubated at 30 °C for 30 min. After incubation, an equal volume of 0.01% bromophenol blue and 0.01% xylene cyanol dissolved in formamide was added and incubated at 95 °C for 5 min. Elongated RNA was separated according to the same protocol of primer extension assay described above.

Northern blot analysis

Cells were infected with RSV (A2) at MOI of 3. At 24 hpi, total RNA was extracted using Rneasy plus mini kit (QIAGEN). Ten micrograms of total RNA were fractioned using 1.2% agaroseformaldehyde gels and visualized with ethidium bromide. Digoxigenin (DIG)-labeled RNA probes against P and M genes using a labeling kit (Roche, Basel, Switzerland), detailed shown in Table S1. Isolated RNA was transferred to a nylon membrane, then these were hybridized the probes at 68 °C for 16 h. Finally, each mRNA was detected using an anti-DIG antibody conjugated with peroxidase (Roche). Table S1. Primers used for PCR of P and M gene fragments.
