# Supplementary material for: Sparfloxacin reveals a host–virus dual-target antiviral scaffold against respiratory syncytial virus
Source: Commun Biol. 2026 Aug 8;9:1434. doi: 10.1038/s42003-026-10744-5 (PMC13722688; doi:10.1038/s42003-026-10744-5)
Supplement: Supplementary file 3 [file 42003_2026_10744_MOESM3_ESM.pdf]

Description of Additional Supplementary Files:

File: Supplementary Data 1

Description: Source data for the figures.
