# Supplementary material for: Sparfloxacin reveals a host–virus dual-target antiviral scaffold against respiratory syncytial virus
Source: Commun Biol. 2026 Aug 8;9:1434. doi: 10.1038/s42003-026-10744-5 (PMC13722688; doi:10.1038/s42003-026-10744-5)
Supplement: Supplementary file 4 [file 42003_2026_10744_MOESM5_ESM.pdf]

nature portfolio

Corresponding author(s): DAPR NCOMMS-25-45148-T

Last updated by author(s): May 7, 2026

Reporting Summary

Nature Portfolio wishes to improve the reproducibility of the work that we publish. This form provides structure for consistency and transparency in reporting. For further information on Nature Portfolio policies, see our Editorial Policies and the Editorial Policy Checklist.

Statistics

For all statistical analyses, confirm that the following items are present in the figure legend, table legend, main text, or Methods section.

n/a	Confirmed	
☐	☒	The exact sample size (n) for each experimental group/condition, given as a discrete number and unit of measurement
☐	☒	A statement on whether measurements were taken from distinct samples or whether the same sample was measured repeatedly
☐	☒	The statistical test(s) used AND whether they are one- or two-sided<br>*Only common tests should be described solely by name; describe more complex techniques in the Methods section.*
☒	☐	A description of all covariates tested
☐	☒	A description of any assumptions or corrections, such as tests of normality and adjustment for multiple comparisons
☐	☒	A full description of the statistical parameters including central tendency (e.g. means) or other basic estimates (e.g. regression coefficient) AND variation (e.g. standard deviation) or associated estimates of uncertainty (e.g. confidence intervals)
☐	☒	For null hypothesis testing, the test statistic (e.g. F, t, r) with confidence intervals, effect sizes, degrees of freedom and P value noted<br>*Give P values as exact values whenever suitable.*
☒	☐	For Bayesian analysis, information on the choice of priors and Markov chain Monte Carlo settings
☐	☒	For hierarchical and complex designs, identification of the appropriate level for tests and full reporting of outcomes
☒	☐	Estimates of effect sizes (e.g. Cohen's d, Pearson's r), indicating how they were calculated

Our web collection on statistics for biologists contains articles on many of the points above.

Software and code

Policy information about availability of computer code

Data collection	Living Image software (v4.8.2, Perkin Elmer) was used for IVIS bioluminescence image acquisition and quantification. QuantStudio Design and Analysis software (Thermo Fisher Scientific) was used for qPCR data acquisition. No custom computer code was used for data collection.
Data analysis	GraphPad Prism 10 (GraphPad Software) and R version 4.4.2 were used for statistical analyses and data visualization. RNA-seq analyses were performed using standard bioinformatics pipelines as described in the Methods section. Molecular docking analyses were performed using AutoDock Vina and visualized using PyMOL. No custom algorithms central to the study were developed.

For manuscripts utilizing custom algorithms or software that are central to the research but not yet described in published literature, software must be made available to editors and reviewers. We strongly encourage code deposition in a community repository (e.g. GitHub). See the Nature Portfolio guidelines for submitting code & software for further information.

Data

Policy information about availability of data

All manuscripts must include a data availability statement. This statement should provide the following information, where applicable:

- Accession codes, unique identifiers, or web links for publicly available datasets
- A description of any restrictions on data availability
- For clinical datasets or third party data, please ensure that the statement adheres to our policy

The data supporting the findings of this study are available within the paper and its Supplementary Information files. RNA-seq data have been deposited in the NCBI BioProject database under accession number PRJNA1282552 and will be publicly released upon publication. Source data are provided with this paper.

Research involving human participants, their data, or biological material

Policy information about studies with human participants or human data. See also policy information about sex, gender (identity/presentation), and sexual orientation and race, ethnicity and racism.

Reporting on sex and gender	Sex and gender were not considered as variables in this study because the primary objective was mechanistic evaluation of RSV infection and antiviral responses in experimental cell and animal models. Human tissue samples were obtained from pediatric donors undergoing clinically indicated surgery.
Reporting on race, ethnicity, or other socially relevant groupings	Race, ethnicity, and other socially relevant groupings were not collected or analyzed in this study.
Population characteristics	Human adenoid tissues were obtained from pediatric patients undergoing routine adenoidectomy procedures at Sapporo Medical University Hospital. Samples were anonymized prior to experimental use.
Recruitment	Human tissue samples were obtained from pediatric patients undergoing clinically indicated adenoidectomy procedures. Samples were collected after informed consent from guardians under institutional ethical approval.
Ethics oversight	The study protocol involving human-derived materials was approved by the Institutional Review Board of Sapporo Medical University. Written informed consent was obtained from the guardians of all participants prior to tissue collection.

Note that full information on the approval of the study protocol must also be provided in the manuscript.

Field-specific reporting

Please select the one below that is the best fit for your research. If you are not sure, read the appropriate sections before making your selection.

☒ Life sciences ☐ Behavioural & social sciences ☐ Ecological, evolutionary & environmental sciences

For a reference copy of the document with all sections, see nature.com/documents/nr-reporting-summary-flat.pdf

Life sciences study design

All studies must disclose on these points even when the disclosure is negative.

Sample size	Sample sizes were determined based on prior RSV infection and antiviral studies in the field, pilot experiments, and feasibility considerations. No formal statistical method was used to predetermine sample size.
Data exclusions	No data were excluded from the analyses unless technical failure occurred prior to predefined endpoint acquisition. Repeated longitudinal measurements obtained from the same animal were not treated as independent biological replicates.
Replication	All in vitro experiments were independently repeated at least three times unless otherwise stated. Animal experiments were performed using biologically independent animals, and representative findings were reproduced in independent experiments.
Randomization	Mice were allocated to experimental groups based on age- and sex-matched distribution. No additional randomization method was used.
Blinding	Investigators were not blinded during animal experiments or outcome assessment because treatment allocation and in vivo imaging procedures required direct handling of experimental groups.

Reporting for specific materials, systems and methods

We require information from authors about some types of materials, experimental systems and methods used in many studies. Here, indicate whether each material, system or method listed is relevant to your study. If you are not sure if a list item applies to your research, read the appropriate section before selecting a response.

Materials & experimental systems

n/a	Involved in the study
☐	☒ Antibodies
☐	☒ Eukaryotic cell lines
☒	☐ Palaeontology and archaeology
☐	☒ Animals and other organisms
☒	☐ Clinical data
☒	☐ Dual use research of concern
☒	☐ Plants

Methods

n/a	Involved in the study
☒	☐ ChIP-seq
☒	☐ Flow cytometry
☒	☐ MRI-based neuroimaging

Antibodies

Antibodies used	Primary antibodies used in this study are described in the Methods section and Supplementary Table X, including supplier information, catalog numbers, clone names, and dilution conditions.
Validation	Antibodies were validated by the manufacturers for the indicated applications and species. Additional validation was supported by expected molecular weights, staining patterns, positive and negative controls, and consistency with previously published studies.

Eukaryotic cell lines

Policy information about cell lines and Sex and Gender in Research

Cell line source(s)	A549 and HEp-2 cells were obtained from ATCC. BEAS-2B cells were obtained from the RIKEN BioResource Center. Primary pediatric adenoid epithelial cells were established from surgical specimens obtained at Sapporo Medical University Hospital under institutional ethical approval. Sex information for primary donor-derived cells was not collected or analyzed in this study.
Authentication	Cell line authentication was not additionally performed after acquisition from the indicated repositories.
Mycoplasma contamination	All cell lines used in this study were routinely tested and confirmed negative for mycoplasma contamination.
Commonly misidentified lines (See ICLAC register)	HEp-2 cells are listed in the ICLAC register of commonly misidentified cell lines due to HeLa contamination; however, they were used because they remain a standard experimental system for RSV propagation and antiviral assays.

Animals and other research organisms

Policy information about studies involving animals; ARRIVE guidelines recommended for reporting animal research, and Sex and Gender in Research

Laboratory animals	The study used 8-week-old female BALB/c mice obtained from Sankyo Labo Service Co. (Tokyo, Japan).
Wild animals	This study did not involve wild animals.
Reporting on sex	All animal experiments were performed using female BALB/c mice. Sex was not evaluated as an experimental variable because the primary objective of the study was mechanistic evaluation of antiviral activity in a standardized RSV infection model.
Field-collected samples	This study did not involve field-collected samples.
Ethics oversight	All animal experiments were approved by the Animal Care Committee of Sapporo Medical University (approval numbers: H2204:22-034_23-092) and conducted in accordance with institutional guidelines and ARRIVE 2.0 recommendations.

Note that full information on the approval of the study protocol must also be provided in the manuscript.

Plants

Seed stocks	n/a
Novel plant genotypes	n/a
Authentication	n/a
